# Synthesis of New 3-Arylcoumarins Bearing *N*-Benzyl Triazole Moiety: Dual Lipoxygenase and Butyrylcholinesterase Inhibitors With Anti-Amyloid Aggregation and Neuroprotective Properties Against Alzheimer’s Disease

**DOI:** 10.3389/fchem.2021.810233

**Published:** 2022-01-20

**Authors:** Ladan Pourabdi, Tuba Tüylü Küçükkılınç, Fatemeh Khoshtale, Beyza Ayazgök, Hamid Nadri, Farid Farokhi Alashti, Hamid Forootanfar, Tayebeh Akbari, Mohammad Shafiei, Alireza Foroumadi, Mohammad Sharifzadeh, Mehdi Shafiee Ardestani, M. Saeed Abaee, Loghman Firoozpour, Mehdi Khoobi, Mohammad M. Mojtahedi

**Affiliations:** ^1^ Department of Organic Chemistry and Natural Products, Chemistry and Chemical Engineering Research Center of Iran, Tehran, Iran; ^2^ Faculty of Pharmacy, Department of Biochemistry, Hacettepe University, Ankara, Turkey; ^3^ Department of Medicinal Chemistry, Faculty of Pharmacy and Pharmaceutical Sciences Research Center, Shahid Sadoughi University of Medical Sciences, Yazd, Iran; ^4^ Pharmaceutical Sciences and Cosmetic Products Research Center, Kerman University of Medical Sciences, Kerman, Iran; ^5^ Department of Microbiology, Islamic Azad University, North Tehran Branch, Tehran, Iran; ^6^ Department of Medicinal Chemistry, Faculty of Pharmacy, Tehran University of Medical Sciences, Tehran, Iran; ^7^ Pharmaceutical Sciences Research Center, The institute of Pharmaceutical Sciences (TIPS), Tehran University of Medical Sciences, Tehran, Iran; ^8^ Department of Pharmacology and Toxicology, Faculty of Pharmacy, Tehran University of Medical Sciences, Tehran, Iran; ^9^ Department of Radiopharmacy, Faculty of Pharmacy, Tehran University of Medical Sciences, Tehran, Iran

**Keywords:** lipoxygenase inhibition, 3-arylcoumarins, neuroprotective agents, Alzeihmer’s disease, anti-amyloid aggregation

## Abstract

A novel series of coumarin derivatives linked to the *N*-benzyl triazole group were synthesized and evaluated against 15-lipoxygenase (15-LOX), and acetyl- and butyrylcholinesterase (AChE and BuChE) to find the most potent derivative against Alzheimer’s disease (AD). Most of the compounds showed weak to moderate activity against ChEs. Among the most active BuChE and 15-LOX inhibitors, **8l** and **8n** exhibited an excellent neuroprotective effect, higher than the standard drug (quercetin) on the PC12 cell model injured by H_2_O_2_ and significantly reduced aggregation of amyloid Aβ_1-42_, with potencies of 1.44 and 1.79 times higher than donepezil, respectively. Compound **8l** also showed more activity than butylated hydroxytoluene (BHT) as the reference antioxidant agent in reducing the levels of H_2_O_2_ activated by amyloid β in BV2 microglial cells. Kinetic and ligand–enzyme docking studies were also performed for better understanding of the mode of interaction between the best BuChE inhibitor and the enzyme. Considering the acceptable BuChE and 15-LOX inhibition activities as well as significant neuroprotection, and anti-amyloid aggregation activities, **8l** and **8n** could be considered as potential MTDLs for further modification and studies against AD.

## 1 Introduction

Alzheimer’s disease (AD) is a type of age-related and progressive neurodegenerative disorder leading to severe cognitive and psychiatric impairment in elderly individuals. It is considered as the most common cause of age-dependent behavioral decline in older humans (Zhou et al., 2020; Pradeep et al., 2021). The number of AD patients in the United States has been reported to be about 5.7 million in 2018 and is being expected to rise to 13.8 million by 2050. The cost of the treatment is also estimated about 2 trillion USD by 2030 (Dong et al., 2019; Kazmi et al., 2019; Oukoloff et al., 2019; Zhao et al., 2020). During the last 40 years, tacrine, donepezil, rivastigmine, and galantamine were the only four cholinesterase inhibitors launched in the market (De Simone et al., 2020; Miranda et al., 2020) for AD treatment. The high failure rate (99.6%) of the anti-AD drugs entered in clinical trials to meet the prefixed clinical endpoints from 2002 to 2012 clearly demonstrates the urgent and unmet need for the development of new anti-AD drugs with different mechanisms of action (Das Neves et al., 2019).

AD has a multifactorial nature and from the pathophysiologic point of view is associated with amyloid-β (Aβ) plaques and neurofibrillary tangle formation, oxidative stress, neuro-inflammation, and cerebrovascular dysregulation (Royea et al., 2019; Shaimardanova et al., 2020; Shityakov et al., 2021). The decline in the cholinergic system function in the hippocampus is a well-known mechanism in AD pathophysiology (Arshad et al., 2020; Zhang et al., 2020). The memory impairment and behavioral abnormalities in the patients are resulted from the low level of acetylcholine (ACh) (Kazmi et al., 2019). Acetylcholinesterase (AChE) and butyrylcholinesterase (BuChE) belong to α/β-fold protein family, playing an impressive role in early stages of the disease by ACh hydrolysis (Ibrar et al., 2018; Najafi et al., 2019). The level of AChE decreases in the brain to 55–67%, and the level of BuChE increases to 165% of the normal value, as the disease progresses (Chalupova et al., 2019; Xu et al., 2021). Some studies have shown that BuChE inhibitors are able to restore the level of ACh in the brain with much reduced peripheral side effects, especially in advanced stages of the disease (Mamedova et al., 2019; Zhou et al., 2019).

The Aβ peptide is also one of the most studied therapeutic targets in AD. The proteolytic cleavage of the transmembrane glycoprotein amyloid precursor protein results in 37- to 49-amino acid-long Aß fragments, which can be further aggregated and form plaques (Aβ_1-40_ and Aβ_1-42_ peptides) (Rodríguez–Soacha et al., 2019; Coimbra et al., 2018; Fronza et al., 2019; Kohelová et al., 2019; Cai et al., 2020; Li C. et al., 2020; Li L. H. et al., 2020). Therefore, decreasing the Aβ production rate or enhancing the Aβ clearance can be exploited to develop appropriate treatment strategies (Kaur et al., 2020). The ChEs also have a crucial role in increased accumulation of Aβ_1-42_ (Podoly, et al., 2010; Darvesh et al., 2012). The interaction of Aβ with the peripheral anionic binding site (PAS) of AChE promotes amyloid fibril formation (Castro and Martinez, 2001). Recently, Biogen developed aducanumab as a human monoclonal antibody, which selectively targets Aβ fibrils and is approved by the FDA to manage AD (Di Meco and Vassar, 2021).

Besides, several pieces of evidence suggest the role of oxidative stress in abnormal deposition of Aβ peptides (Schewe, 2002; Sestito et al., 2019). Lipoxygenases (LOXs) are important enzymes that catalyze the peroxidation of specific atoms in polyunsaturated fatty acids (PUFAs). The 15-LOX has been implicated in neurodegenerative diseases including AD (Cabezas and Mascayano, 2019; Prismawan et al., 2019; Koh et al., 2003; Mphahlele et al., 2019a).

In modern medicinal chemistry, multi-target–directed ligand (MTDL) strategy is a well-established approach for the design and discovery of the multifunctional compounds modulating various receptors or targeting diverse enzymes to employ the potential treatment of complex and multifunctional disorders like AD (Oset–Gasque and Marco–Contelles, 2018; Costanzo et al., 2021). Multifactorial cause of AD requires a multi-target approach for the treatment. Such an approach can be achieved through the combination of effective pharmacophoric groups in a unique small molecule. This paradigm is more effective than one-target, one-drug concept (Schewe, 2002; Oset–Gasque and Marco–Contelles, 2018; Jończyk et al., 2019; Mphahlele et al., 2019b). Drug combination therapies for the treatment of AD lead to more beneficial therapeutic effects and resulted in the superior *in vivo* outcomes compared to the one-target compounds having high affinity, even if the multi-target small molecules have mild activity against one or several targets (Morphy and Rankovic, 2007). The synergy between inhibiting an enzyme and activating or blocking a receptor is the advantage achieved with the use of MTDLs (Cai et al., 2020; Mphahlele et al., 2019b; Montanari et al., 2019; Kashyap et al., 2020; Tripathi et al., 2019).

**FIGURE 1:**
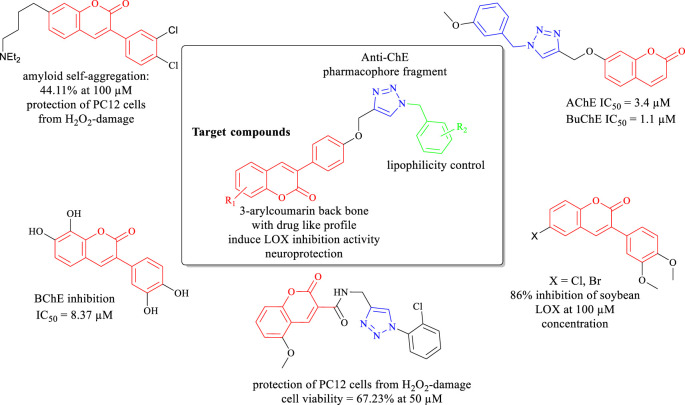
Design strategy for the target compounds based on 3-arylcoumarin derivatives bearing *N*-benzyl triazole moiety.

Due to the pharmacological and physicochemical properties of the coumarin scaffold with the oxa-heterocyclic ring, various hybrid structures of coumarin with significant biological activities have been studied so far. These hybrids have shown great effect on the central nervous system and attracted great interest in neurodegenerative disorder studies (Jalili–Baleh et al., 2018c; Seong et al., 2019; Tripathi and Ayyannan, 2019). Till now, several research groups have developed multifunctional ligands having interesting results as MTDLs against AD based on coumarin derivatives bearing an *N*-benzyl moiety (Rahmani–Nezhad et al., 2015; Piazzi et al., 2008; Abdshahzadeh et al., 2019; Kumari et al., 2017; Saeedi et al., 2017; Jalili–Baleh et al., 2018b) or cross-linked to appropriate pharmacophores *via* 1,2,3-triazoles (Figure 1) (Bozorov et al., 2019; Arafa and Nayl, 2019; Lipeeva, et al., 2019; Asgari et al., 2019; Xu, et al., 2019).

In this work and for further progressing in our research program on the discovery of new coumarin derivatives as MTDLs against AD (Jalili-Baleh et al., 2018a; Salehi et al., 2019), we prepared new 3-arylcoumarin derivatives bearing *N*-benzyl triazole substructures (Figure 1) to target 15-LOX and ChEs, with neuroprotection activity and anti-Aβ aggregation activity, simultaneously. Most of the previously published studies tried to improve the ChE inhibition and neuroprotection/antioxidant activities of the coumarin backbone by conjugation of the appropriate pharmacophores. To the best of our knowledge, there is no attempt to introduce coumarin derivatives having remarkable LOX inhibition activity together with appropriate ChE/neuroprotection/antioxidant activity. 3-Arylcoumarins bearing appropriate hydrophobic aryl groups had the chance to be well connected to the active site of the LOX through hydrophobic interactions (Roussaki et al., 2010). Preliminary docking studies encouraged us to conjugate the 3-arylcoumarin scaffold to *N*-benzyl triazole fragments as the well-known pharmacophoric groups playing crucial role in anti-ChE activity (Figure 1) (Kiani et al., 2019). The main aim of this study was to endow the 3-arylcoumarin backbone with more inhibition activities through conjugation with *N*-benzyl triazoles to find the optimum multi-target small molecules against AD.

## 2 Results and Discussion

### 2.1. Chemistry

**SCHEME 1:**
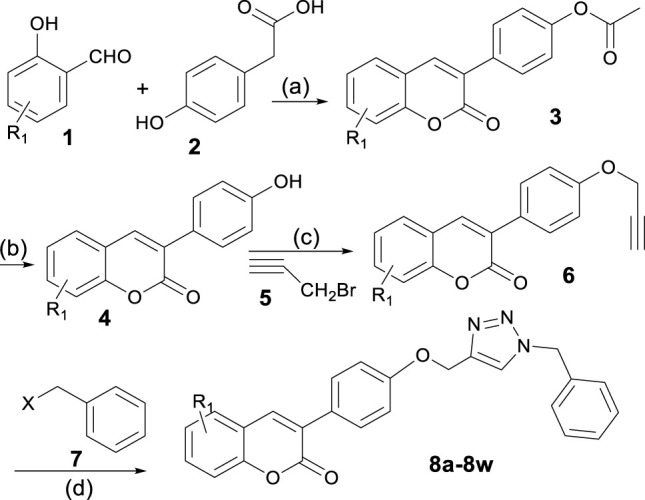
Synthesis of compounds **8a**-**8w**. **(A)** KOAc, Ac_2_O, reflux, 4 h; **(B)** HCl (2N), MeOH, reflux, 3 h; **(C)** K_2_CO_3_, DMF, 60–65°C, 4 h; and **(D)** sodium ascorbate (NaAs), CuSO_4_, *tert*-butanol:H_2_O (1:1), r. t., 4 h.

A four-step process was developed for the controlled synthesis of the target compounds **8a**-**8w**, as shown in scheme 1. Intermediate **4** was synthesized *via* hydrolysis of compound **3**, which was in turn prepared through Perkin condensation of different commercially available 2-hydroxybenzaldehydes **1** with 2-(4-hydroxyphenyl)acetic acid **2** (Kabeya et al., 2007). 2*H*-chromen-2-one derivatives **6** were synthesized *via* the reaction between 3-(4-hydroxyphenyl)-2*H*-chromen-2-one derivatives **4** and propargyl bromide **5**. Finally, regioselective formation of 1,2,3-triazoles linked to the coumarin structure (**8**) was carried out by a copper-catalyzed azide–alkyne cycloaddition (CuAAC) *via* a one-pot three-component click reaction. The azides were *in situ* generated from the corresponding benzyl halides **7**.

### 2.2 Biological Screenings

#### 2.2.1 LOX Inhibitory Activity

All the title compounds **8a**-**8w** were tested against 15-LOX enzyme (Table 1). Compounds **8a**-**8d** and **8l**-**8q** exhibited good activities against 15-LOX (IC_50_ = 14.2–45.2 µM), compared to the standard drug, quercetin (IC_50_ = 21.7 µM). Among them, compounds **8b** and **8o** having the 2F-substituted phenyl group showed the highest inhibitory activity against the enzyme (IC_50_ = 16.5 and 14.2 µM, respectively), more active than quercetin as the standard drug (IC_50_ = 21.7 µM). Comparison of the compounds having an unsubstituted benzyl triazole group showed a decrease in activity in the order of coumarin > 8-methoxycoumarin > 6-nitrocoumarin. Also, the order of LOX inhibition activity for the compounds bearing fluorine substitution was 2-F > 3-F > 4-F (compare compounds **8b** with **8c** and **8d** or **8o** with **8p** and **8q**). The results showed that the size and polarity of the halogen group on the benzyl triazole part had a significant effect on the LOX inhibitory effect of the target compounds (F > Br > Cl).

**TABLE 1:** AChE, BuChE, and 15-LOX inhibitory activity of the synthesized compound **8a**-**8w**.

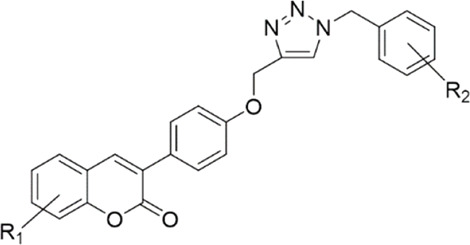				
Comp.	R_1_	R_2_	15-LOX	eq BuChE	
			IC_50_ µM	%inhib.^a^	IC_50_ µM^b^
**8a**	H	H	36.2 ± 1.5	—	27.6 ± 1.3
**8b**	H	2-F	16.5 ± 0.7	—	36.5 ± 1.1
**8c**	H	3-F	28.3 ± 1.4	48.9	—
**8d**	H	4-F	45.2 ± 1.5	12.7	—
**8e**	H	2-Cl	>100	—	—
**8f**	H	4-Cl	>100	23.6	—
**8g**	H	4-Br	>100	46.7	—
**8h**	H	2-NO_2_	>100	—	NT
**8i**	H	3-NO_2_	>100	—	NT
**8j**	H	4-NO_2_	>100	—	NT
**8k**	H	3-Me	53.5 ± 1.5	37.1	—
**8l**	H	3-OMe	39.1 ± 1.1	—	19.5 ± 0.9
**8m**	H	4-OMe	41.0 ± 1.4	40.2	-
**8n**	8-OMe	H	42.5 ± 1.7	-	45.1 ± 1.3
**8o**	8-OMe	2-F	14.2 ± 0.4	8.0	—
**8p**	8-OMe	3-F	22.6 ± 0.8	41.7	—
**8q**	8-OMe	4-F	35.0 ± 1.3	—	18.9 ± 0.9
**8r**	8-OMe	4-Cl	>100	—	6.7 ± 0.4
**8s**	8-OMe	2- ${\text{NO}}_{2}$	>100	42.5	—
**8t**	6-Br	4-Br	>100	—	NT
**8u**	6-Br	2-F	61.5 ± 1.9	46.6	—
**8v**	6-NO_2_	H	>100	—	6.3 ± 0.4
**8w**	6-NO_2_	2-F	>100	30.4	—
Tacrine	—	—	—	—	0.073 ± 0.009
Quercetin	—	—	21.7 ± 0.7	—	—

a Inhibitor concentration required for 50% inactivation (mean ± SEM, of three experiments). BuChE (from equine serum) was applied in this study.

b Not tested.

#### 2.2.2 Cholinesterase Inhibition

All the target compounds **8a**-**8w** were also assessed for their *in vitro* AChE and BChE inhibitory activities using Ellman’s spectrophotometric method, while tacrine was used as the standard drug. All data were presented as the mean ± SD of three independent experiments. The results are listed in Table 1 as IC_50_ values.

The compounds exhibited weak or no activity against AChE, and the type/position of the substituents had no great effect on the activity. The compounds also showed weak to moderate inhibitory effect against BuChE. Among unsubstituted coumarin derivatives, **8a**, **8b**, and **8l** exhibited mild inhibitory activity against BuChE (IC_50_ values of 27.6, 36.5, and 19.5 µM, respectively). Among compounds bearing the 8-methoxy group on the coumarin ring (**8n**-**s**), **8q** and particularly **8r** exhibited highest inhibitory effects (IC_50_ values = 18.9 and 6.7 µM, respectively). 6-Bromocoumarin derivatives (**8t** and **8u**) showed no activity against BuChE. Among all the prepared compounds, **8v** bearing the 6-nitrocoumarin moiety showed the best inhibitory activity against BuChE (IC_50_ = 6.3 µM). Comparison of the IC_50_ values of the compounds having the same substituted phenyl group (**8a**, **8n**, and **8v**) revealed that the order of the activity was 6-nitrocoumarin > simple coumarin > 8-methoxycoumarin for BuChE inhibition. Changing the type/position of the substituent at the benzyl part had no significant role on the BuChE inhibitory effect of the target compounds. The results showed that the compounds were more potent for BuChE inhibition than AChE.

#### 2.2.3 Neuroprotection Potency Against H_2_O_2_-Induced Cell Death

The most potent compounds with the highest anti-BuChE activity and LOX inhibitory activity were evaluated for neuroprotective activity against the H_2_O_2_-induced PC12 cell death using MTT assay at different concentrations of 0.1, 1.0, 5.0, 10.0, 20.0, and 50.0 µM. All of the compounds could remarkably improve the PC12 cell viability in the presence of H_2_O_2_ in a dose-dependent manner in comparison with the reference drug, except for compounds **8c** and **8v** (Table 2). Most of the compounds significantly increased the cell viability even at low concentration (*p* < 0.001). In particular, **8n** bearing the 8-methoxy group on the coumarin ring was the best neuroprotective agent showing even more activity than quercetin as the reference drug at all concentrations. Notably, the neuroprotective effect of compounds **8l** and **8n** was higher than that of quercetin at a high concentration of 50.0 µM.

**TABLE 2:** Protective effect of the selected compounds against H_2_O_2_-induced PC12 cell death at different concentrations.^a^

Code	R_1_	R_2_	PC12 cell viability (% of control)						
			H_2_O_2_	0.1 µM	1 µM	5 µM	10 µM	20 µM	50 µM
**8a**	H	H	21.6 ± 0.4	29.3 ± 0.3***	31.0 ± 0.5***	37.3 ± 0.5***	40.8 ± 0.3***	43.3 ± 0.4***	44.7 ± 0.4***
**8b**	H	2-F	21.8 ± 0.3	22.7 ± 0.5^ns^	25.0 ± 0.3***	28.3 ± 0.2***	30.4 ± 0.3***	32.2 ± 0.2***	33.7 ± 0.4***
**8c**	H	3-F	25.7 ± 0.4	25.3 ± 0.5^ns^	27.6 ± 0.4**	29.8 ± 0.3***	31.5 ± 0.4***	32.8 ± 0.5***	33.8 ± 0.2***
**8d**	H	4-F	26.8 ± 0.1	29.5 ± 0.3***	32.6 ± 0.3***	36.4 ± 0.2***	40.6 ± 0.4***	43.6 ± 0.3***	46.3 ± 0.2***
**8l**	H	3-MeO	21.6 ± 0.7	31.5 ± 0.7***	38.1 ± 0.7***	41.8 ± 0.4***	47.8 ± 0.4***	51.7 ± 0.8***	57.0 ± 0.6***
**8m**	H	4-MeO	22.2 ± 0.4	29.5 ± 0.3***	37.5 ± 0.5***	41.0 ± 0.2***	46.3 ± 0.1***	53.5 ± 0.6***	63.3 ± 0.3***
**8n**	8-MeO	H	24.0 ± 0.6	32.9 ± 0.5***	41.4 ± 0.6***	49.5 ± 0.3***	55.2 ± 0.6***	60.0 ± 0.5***	65.4 ± 0.3***
**8o**	8-MeO	2-F	22.1 ± 0.3	29.5 ± 0.3***	34.5 ± 0.4***	40.2 ± 0.4***	43.0 ± 0.3***	47.2 ± 0.5***	51.1 ± 0.6***
**8p**	8-MeO	3-F	22.1 ± 0.2	28.1 ± 0.2***	34.6 ± 0.4***	38.5 ± 0.5***	44.6 ± 0.8***	47.8 ± 0.5***	52.0 ± 0.6***
**8q**	8-MeO	4-F	22.0 ± 0.2	30.5 ± 0.6***	33.4 ± 0.6***	36.1 ± 0.3***	40.4 ± 0.2***	42.8 ± 0.4***	46.2 ± 0.1***
**8r**	8-MeO	4-Cl	23.4 ± 0.6	28.4 ± 0.1***	32.6 ± 0.2***	36.1 ± 0.7***	43.4 ± 0.8***	49.7 ± 0.4***	55.5 ± 0.7***
**8v**	6-NO_2_	H	23.7 ± 0.3	23.3 ± 0.2 ^ns^	24.6 ± 0.3 ^ns^	25.3 ± 0.3***	26.5 ± 0.2***	28.1 ± 0.4***	29.6 ± 0.1***
Quercetin	—	—	25.9 ± 0.6	34.8 ± 0.4***	40.9 ± 0.8***	46.9 ± 0.7***	51.6 ± 0.7***	55.1 ± 0.7***	56.4 ± 0.5***

a MTT assay protocol was used to determine the cell viability. The mean ± SEM, of three independent experiments was used to express data. The significant (***p < 0.001, * **p < 0.01) and not significant (ns) values versus H_2_O_2_-treated group.

#### 2.2.4 Cytotoxic Effect

The attained results of cytotoxic evaluation of **8l** and **8n** against the PC12 cell line are represented in Table 3. Compound **8l** (50 µM) inhibited PC12 by a viability percent of 96.5 ± 0.9%, while **8n** exhibited 95.2 ± 1.1% viability at the same concentration. Tacrine as the standard compound exhibited a viability percent of 67.3 ± 1.3% at 50 µM. Thus, it can be concluded that both selected compounds were non-cytotoxic against PC12 at an applied concentration range.

**TABLE 3:** Inhibition potency of the target compounds (**8l** and **8n**) against self- and AChE-induced Aβ_1-42_ aggregation.

Self-induced^a^	Means ± SEM	
**8l**	61.3 ± 4.8	***
**8n**	76.2 ± 6.4	***
Donepezil	42.5 ± 0.3	***
AChE-induced^b^		
**8l**	36.2 ± 6.1	***
**8n**	25.1 ± 8.7	*
Donepezil	78.3 ± 5.0	***

***p < 0.0001; *p < 0.05; compared to the untreated control. Aβ_1-42_ aggregation assay was performed by ThT assay. The mean ± SEM of three independent experiments was used to express data.

a Inhibition of self-induced Aβ_1-42_ aggregation (10.0 μM) by the tested compounds (100.0 μM).

b Inhibition activity of the compound (100.0 μM) against aggregation of Aβ_1-42_ in the presence of AChE (0.01 u/ml).

#### 2.2.5 Other Biological Assessment for the Selected Compounds (8l and 8n)

The main objective of this study was to find the best compounds having a multi-target profile, rather than finding the most active ChE inhibitors. The MTDL strategy has shed light on the drug discovery process and creation of efficient multifunctional small molecules against multifactorial disorders like AD. MTDLs, even with mild activity against one or several targets, could achieve better *in vivo* results than the one-target compounds with high affinity (Morphy and Rankovic, 2007). It has been suggested that low affinity of the MTDLs could be sufficient to generate significant *in vivo* outcomes due to the presence of week connections mostly controlling the cellular networks (Korcsmaros et al., 2007). Based on the obtained results in this work and the importance of multi-target affinity even in moderate activity rather than high-affinity single-target inhibitors, compounds **8l** and **8n** with significant neuroprotective activities (more active than/as level as quercetin), appropriate BuChE activity (IC_50_ = 19.5 and 45.1 µM, respectively), and significant LOX inhibitory potency (IC_50_ = 39.1 and 42.5 µM, respectively) were selected for subsequent biological assessment to check the probable effectiveness of the compounds against AD.

#### 2.2.6 Kinetic Studies

**FIGURE 2:**
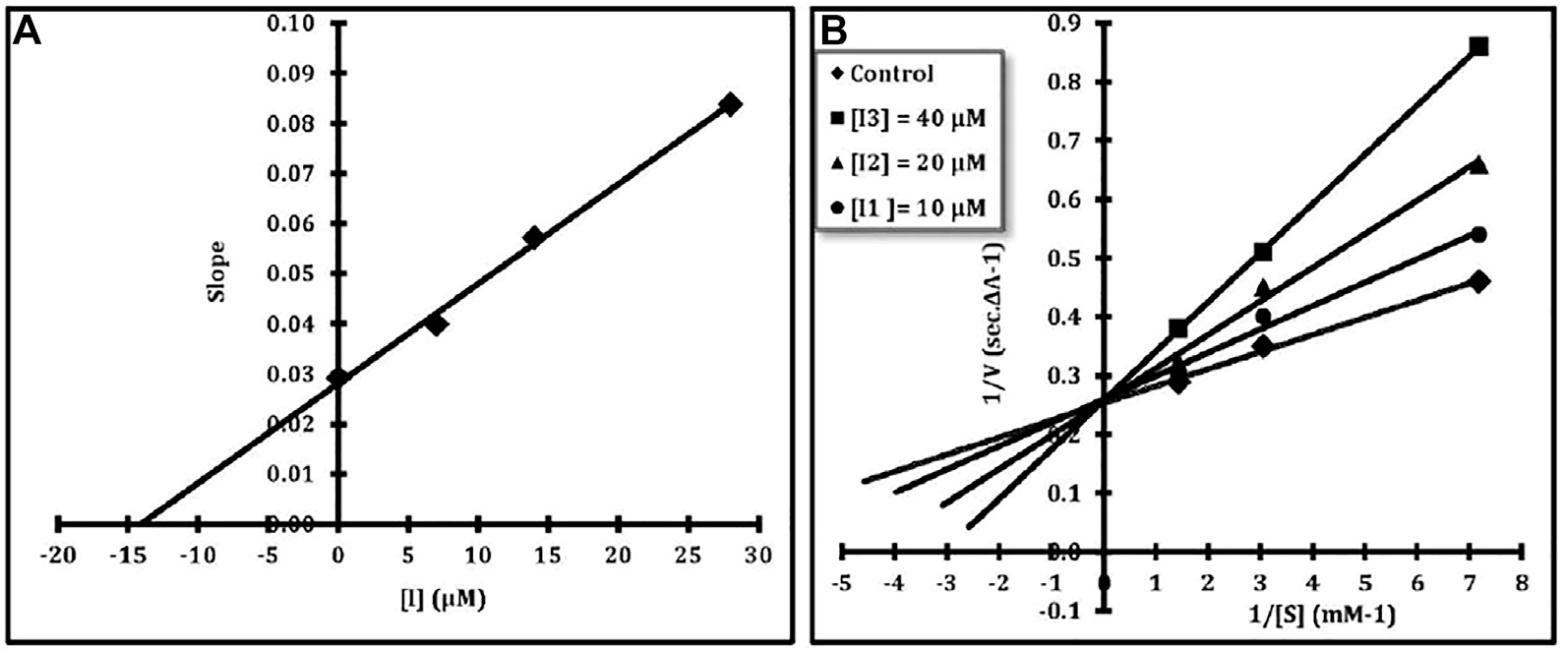
**(A)** Lineweaver–Burk plot for the inhibition of BuChE by compound **8l** in the presence of various concentrations of the substrate (BuTCh). **(B)** Secondary plot for calculation of steady-state inhibition constant of compound **8l** (Ki = 14.1 μM).

Kinetics of the BuChE inhibition activity was studied for compound **8l** at different inhibitor concentrations (0, 10.0, 20.0, and 40.0 µM) as mentioned in the ChE inhibition assay section. Different concentrations of the substrate (S) and butyrylthiocholine iodide (BuChI) were applied to obtain the initial velocity measurements, in each case. The reciprocal of the S (1/S) was plotted against the initial velocity (1/v) (Rampa et al., 2000). The double reciprocal (Lineweaver–Burk) plot revealed that the slopes and intercepts are increased by an increase in the inhibitor concentration. However, compound **8l** showed competitive inhibition. The inhibitory constant (*K*i) of compound **8l** was calculated using the secondary plot, as shown in Figure 2 (*K*i = 14.1 μM).

#### 2.2.7 Molecular Docking Studies

**FIGURE 3:**
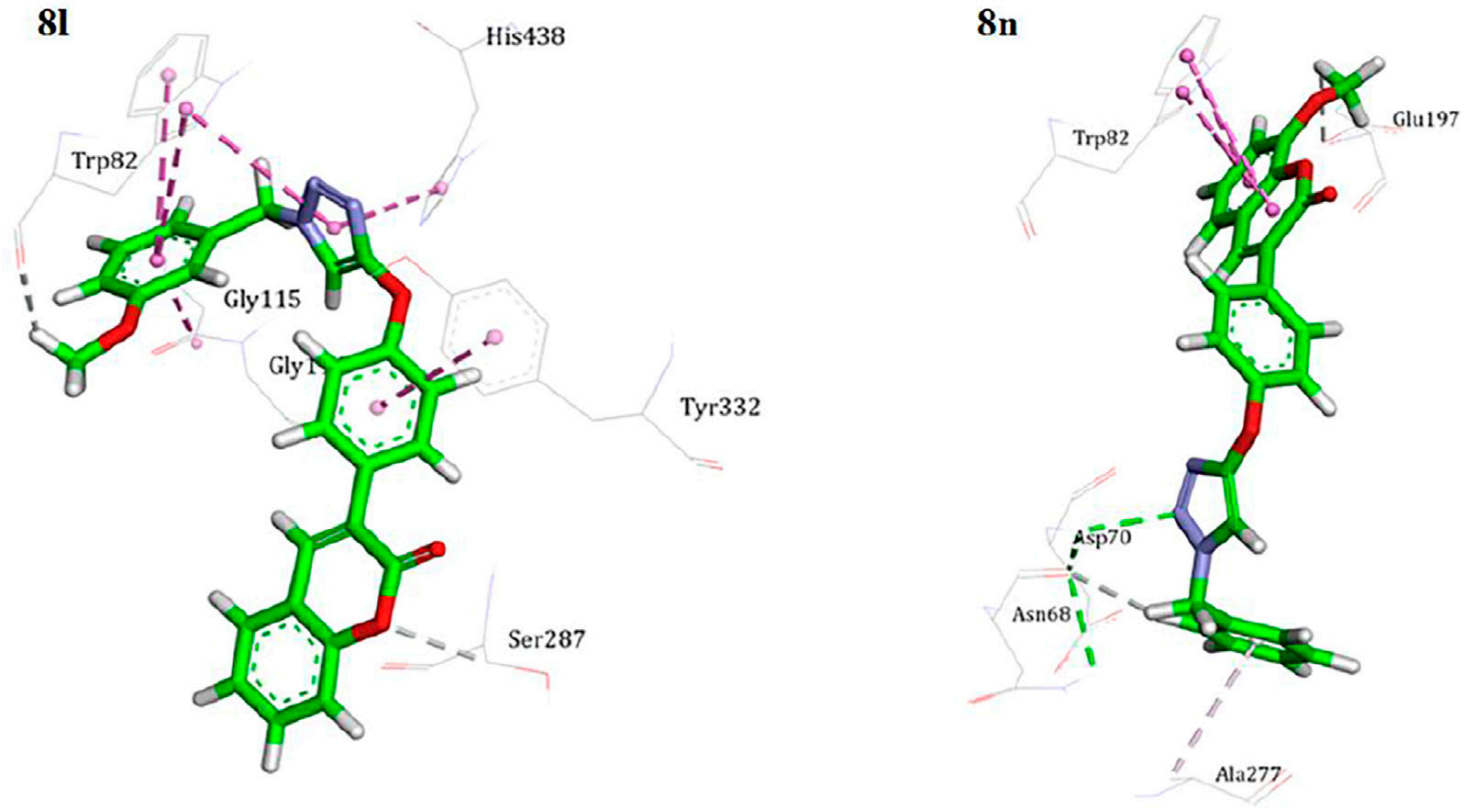
Binding mode of compounds **8l** and **8n** into the active site of BuChE.

Docking studies were carried out to study the binding mode of the target compound within BuChE. To validate the method, tacrine was re-docked into the active site of BuChE (4BDS). The RMSD value was less than 1. The best-docked poses of the best BuChE inhibitor having appropriate neuroprotective effect (**8l**) in the binding site of BuChE are shown in Figure 3.

Hydrophobic and π–π interactions were dominant in the attachment of the ligand to the active site of the enzyme. Compound **8l** was bound to the catalytic triad *via* an *N*-benzyl triazole fragment. The benzyl moiety stacked with Trp82 and the triazole ring made two T-shaped interactions with His438 and Trp82. The coumarin core leaned toward the rim of the enzyme, and the structure was stabilized through π stacking with Tyr332. In the case of **8n**, the interaction profile was quite different. The coumarin moiety lay at the bottom of the active site *via* π stacking interaction with Trp82. The *N*-benzyl triazole tail formed a hydrogen bond with Asp70 at the gate of the active site. All the results revealed the important role of the coumarin and especially triazole parts in BuChE inhibition.

**FIGURE 4:**
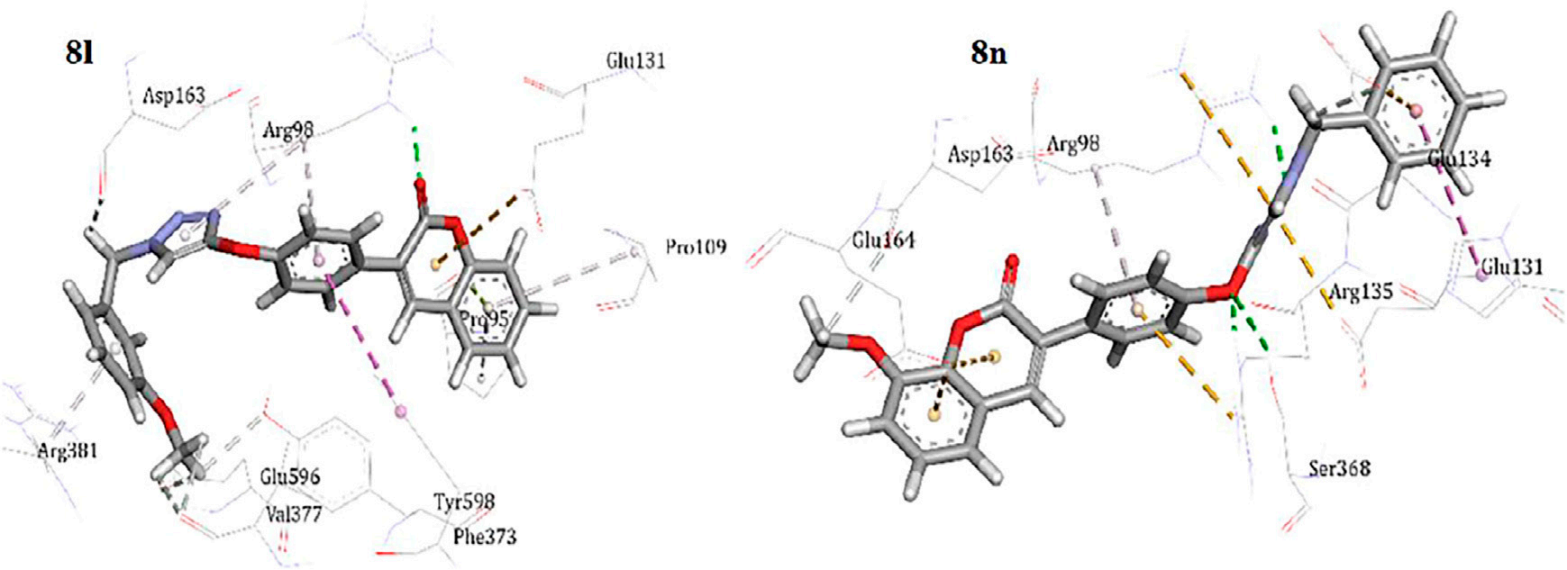
Binding mode of compounds **8l** and **8n** into the active site of LOX.

As shown in Figure 4, compounds **8l** and **8n** well fitted in the active site of LOX, making hydrophobic and non-hydrophobic interactions. Compound **8l** binds to the active site through the hydrophobic interaction with residues Pro95, Pro109, and Arg381 side chains. The carbonyl group of the coumarin rings also hydrogen-bonded to Ser368. Compound **8n** formed hydrophobic interactions with various amino acid side chains such as Glu164, Glu131, Glu134, and Val377. A hydrogen–pi interaction between Arg98 and the phenoxy ring, and a dipole interaction of Arg98 with the carbonyl group of the coumarin ring can control the compound accommodation in the active site of LOX (Nishio 2011; Du et al., 2013). The studied compounds **8l** and **8n** did not show any interaction with the Fe^2+^ ion of the LOX active site.

#### 2.2.8 Anti-Amyloid Aggregation

The inhibition activity of the selected compounds (**8l** and **8n**) against self-induced and AChE-induced Aβ aggregation was determined using thioflavin T (ThT) fluorescence analysis (Table 3). The selected compounds revealed significant inhibitory activity against self-induced Aβ aggregation (61.3 and 76.2% inhibition at 100.0 μM concentration, respectively), which were 1.44 and 1.79 times more than that of the reference drug, donepezil (42.5% at 100.0 μM concentration). The higher inhibitory activity of **8n**, compared to **8l**, suggests the great effect of 8-methoxycoumarin backbone on the interaction with Aβ. The potential of compounds **8l** and **8n** to inhibit Aβ-aggregation induced by AChE were also evaluated. Although the compounds exhibited significant anti-Aβ self-aggregation activity, they had no activity to inhibit AChE-induced Aβ aggregation, which could be due to the week AChE inhibition activity of the target compounds.

#### 2.2.9 Hydrogen Peroxide Cell-Based Assay

**FIGURE 5:**
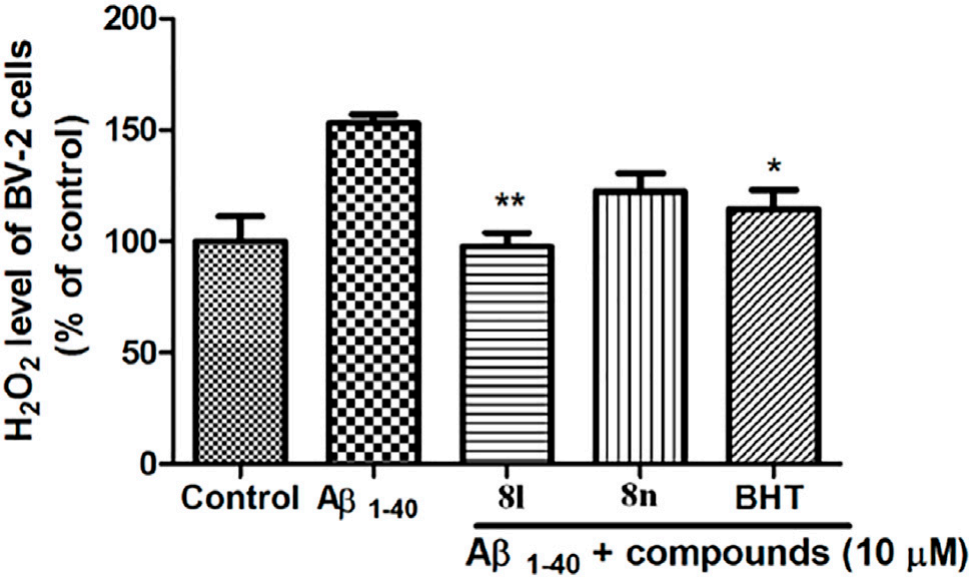
Extracellular H_2_O_2_ produced by the BV-2 cells treated by compounds **8l** and **8n**.

Toxic Aβ peptide fibrils interact with microglial cells and monocytes to stimulate ROS (reactive oxygen species) generation and neuroinflammation, which plays pivotal roles in the pathogenesis of neurodegeneration in AD. Compounds **8l** and **8n** were tested for the reduction of the extracellular H_2_O_2_ produced by the BV-2 cells. As shown in Table 4 and Figure 5, **8l** could significantly reduce the levels of H_2_O_2_ induced by amyloid β in BV2 microglial cells in comparison with butylated hydroxytoluene (BHT, Cell Biolabs) as the reference antioxidant agent.

**TABLE 4:** Extracellular H_2_O_2_ produced by the BV-2 cells treated by compounds **8l** and **8n**.

	H_2_O_2_ level of BV-2 cells (% ± SEM of control)	
Control	100.1 ± 11.2	
Αβ	153.1 ± 3.7	
**8l** + Αβ	97.6 ± 6.1	**
**8n** + Αβ	122.4 ± 8.3	
BHT + Αβ	114.4 ± 8.5	*

**p < 0.01; *p < 0.05; compared to amyloid β_1-40_-induced BV-2, cells.

## 3 Experimental

### 3.1 Chemistry

2-Hydroxybenzaldehyde (**1**), 2-(4-hydroxyphenyl)acetic acid (**2**), propargyl bromide (**5**), benzyl halide derivative (**7**), butyrylcholinesterase (BuChE, E.C. 3.1.1.8), acetylcholinesterase (AChE, E.C. 3.1.1.7), 5,5-dithiobis-(2-nitrobenzoic acid) (DTNB), butyrylthiocholine iodide, dipotassium hydrogen phosphate, potassium dihydrogen phosphate, potassium hydroxide, acetylthiocholine iodide, sodium hydrogen carbonate, soybean lipoxygenase (type 1-B lyophilized powder >50,000 units/mg solid), tacrine, donepezil, quercetin, amyloid β_1-40_, and all other starting materials, reagents, and solvents were purchased from Merck or Aldrich and used as received without more purification. FT–IR spectra (KBr disks) were taken on a Bruker Vector-22 infrared spectrometer, and absorptions were reported as wave numbers (cm^−1^). Nuclear magnetic resonance spectra were recorded on an FT–NMR Bruker Ultra Shield™ (500 MHz for ^1^H and 125 MHz for ^13^C) or Bruker DRX-400 AVANCE (400 MHz for ^1^H and 100 MHz for ^13^C) instruments using TMS as a standard. A CHN-Rapid Heraeus elemental analyzer (within ±0.4% of the calculated values) was used for elemental analysis. Mass spectra were obtained on an Agilent Technology (HP), 5973 Network Mass Selective Detector and Agilent Technology (HP), and 5973 Network Mass Selective Detector at an ionization potential of 70 eV.

#### 3.1.1 General Synthesis of Intermediates 6

A mixture of hydroxylated coumarins (**4**, 2.0 mmol), KI (20.0 mg, 5.0 mol%), and K_2_CO_3_ (0.6 g, 4.3 mmol) in dry DMF (2.0 ml) was heated for 30 min in a 25-ml round-bottom flask, under an argon atmosphere. Propargyl bromide **5** (750.0 µl) was added dropwise within 15 min to the reaction mixture, and the mixture was allowed to stir for 4 h at 60–65°C. The reaction was controlled by TLC. The precipitated solid was filtered and washed with water, after completion of the reaction. Intermediate **6** was obtained by recrystallization from ethanol.

#### 3.1.2 General Synthesis of Compounds 8a-8w

A mixture of NaN_3_ (100 mg, 1.5 mmol), benzyl halide derivative **7** (1.5 mmol), TEA (210.0 µl, 1.5 mmol), and 2.0 ml of *tert*-butanol/H_2_O (1:1) was stirred for 1 h. Compound **6** (0.5 mmol), sodium ascorbate (NaAs, 20.0 mg), and CuSO_4_.5H_2_O (10.0 mg) were then added. The mixture was stirred for another 3 h (monitored by TLC), and *tert*-butanol/H_2_O (3.0 ml, 1:1 v/v) was added to the mixture. After gentle stirring for about 30 min, the obtained solid was filtrated and recrystallized from EtOH to give pure compound **8**.

##### 3.1.2.1 3-(4-((1-Benzyl-1*H*-1,2,3-triazol-4-yl)methoxy)phenyl)-2*H*-chromen-2-one (8a)

Yield 87%; white solid; mp 138–140°C; IR (KBr) 3065 (CH aromatic), 1721 (C=O), 1609 (C=C), 1453 (N=N) cm^−1^; ^1^H NMR (500 MHz, CDCl_3_) *δ* = 7.75 (s, 1H, H_4_ coumarin), 7.65 (d, *J* = 8.0 Hz, 2H), 7.55 (s, 1H, triazole), 7.51 (d, *J* = 7.0 Hz, 1H), 7.49 (dd, *J* = 7.0, 7.0 Hz, 1H), 7.38 (d, *J* = 7.0 Hz, 1H), 7.36 (d, *J* = 9.0 Hz, 2H), 7.29-7.26 (m, 4H), 7.03 (d, *J* = 8.0 Hz, 2H), 5.53 (s, 2H, OCH_2_), 5.23 (s, 2H, NCH_2_). ^13^C NMR (125 MHz, CDCl_3_) *δ* = 160.7 (C=O), 158.7 (C-O), 153.3 (C-O), 144.4 (C-N), 138.7 (CH, Ar), 138.6 (C, Ar), 134.4 (C, Ar), 131.1 (CH, Ar), 129.9 (CH, Ar), 129.2 (CH, Ar), 128.8 (CH, Ar), 128.1 (CH, Ar), 127.7 (CH, Ar), 127.6 (CH, Ar), 124.4 (CHN), 122.7 (C, Ar), 119.8 (C, Ar), 116.4 (CH, Ar), 114.8 (CH, Ar), 62.1 (CH_2_O), 54.3 (CH_2_N). EI-MS m/z (%): 409 (M^+^), 328, 290, 238, 210, 181, 144. Anal. Calcd for C_25_H_19_N_3_O_3_: C, 73.34; H, 4.68; N, 10.26. Found: C, 73.31; H, 4.71; N, 10.28.

##### 3.1.2.2 3-(4-((1-(2-Fluorobenzyl)-1*H*-1,2,3-triazol-4-yl)methoxy)phenyl)-2*H*-chromen-2-one *(8b)*

Yield 79%; white solid; mp 124–126°C; IR (KBr) 3024 (CH aromatic), 1719 (C=O), 1608 (C=C), 1453 (N=N) cm^−1^; ^1^H NMR (500 MHz, CDCl_3_) *δ* = 7.753 (s, 1H, H_4_ coumarin), 7.67 (s, 1H, triazole), 7.65-7.63 (m, 2H), 7.53-7.49 (m, 2H), 7.38-7.34 (m, 2H), 7.29-7.26 (m, 2H), 7.17-7.10 (m, 2H), 7.04 (d, *J* = 8.0 Hz, 2H), 5.60 (s, 2H, OCH_2_), 5.23 (s, 2H, NCH_2_). ^13^C NMR (125 MHz, CDCl_3_) *δ* = 160.8 (d, *J* = 250.0 Hz, CF), 160.7 (C=O), 158.8 (C-O), 153.3 (C-O), 144.4 (C-N), 138.7 (CH, Ar), 138.6 (C, Ar), 131.1 (CH, Ar), 131.0 (CH, Ar), 130.9 (CH, Ar), 130.7 (CH, Ar), 130.6 (CH, Ar), 129.9 (CH, Ar), 127.7 (C, Ar), 124.9 (CH, Ar), 124.4 (CH, Ar), 122.8 (C, Ar), 119.8 (C, Ar), 116.4 (CH, Ar), 115.8 (d, *J* = 21.0 Hz, CH, Ar), 114.8 (CH, Ar), 62.1 (CH_2_O), 47.7 (CH_2_N). EI-MS m/z (%): 427 (M^+^), 290, 238, 210, 162, 109. Anal. Calcd for C_25_H_18_FN_3_O_3_: C, 70.25; H, 4.24; N, 9.83. Found: C, 70.22; H, 4.26; N, 9.85.

##### 3.1.2.3 3-(4-((1-(3-Fluorobenzyl)-1*H*-1,2,3-triazol-4-yl)methoxy)phenyl)-2*H*-chromen-2-one (8c)

Yield 81%; white solid; mp 149–150°C; IR (KBr) 3030 (CH aromatic), 1715 (C=O), 1610 (C=C), 1453 (N=N) cm^−1^; ^1^H NMR (500 MHz, CDCl_3_) *δ* = 7.76 (s, 1H, H_4_ coumarin), 7.66 (d, *J* = 8.0 Hz, 2H), 7.58 (s, 1H, triazole), 7.53-7.49 (m, 2H), 7.37-7.33 (m, 2H), 7.30-7.26 (m, 2H), 7.06-7.03 (m, 3H), 6.97 (d, *J* = 9.0 Hz, 1H), 5.53 (s, 2H, OCH_2_), 5.25 (s, 2H, NCH_2_). ^13^C NMR (125 MHz, CDCl_3_) *δ* = 163.0 (d, *J* = 250 Hz, CF), 160.7 (C=O), 158.7 (C-O), 153.3 (C-O), 144.6 (C-N), 138.8 (CH, Ar), 138.7 (C, Ar), 131.1 (CH, Ar), 130.9 (CH, Ar), 130.8 (CH, Ar), 129.9 (CH, Ar), 127.7 (CH, Ar), 124.5 (CH, Ar), 123.6 (CH, Ar), 122.8 (C, Ar), 122.7 (C, Ar), 119.8 (C, Ar), 116.4 (CH, Ar), 115.8 (d, *J* = 21.0 Hz, CH, Ar), 115.0 (d, *J* = 21.0 Hz, CH, Ar), 114.8 (CH, Ar), 62.1 (CH_2_O), 53.6 (CH_2_N). EI-MS m/z (%): 427 (M^+^), 290, 238, 210, 162, 109. Anal. Calcd for C_25_H_18_FN_3_O_3_: C, 70.25; H, 4.24; N, 9.83. Found: C, 70.22; H, 4.27; N, 9.86.

##### 3.1.2.4 3-(4-((1-(4-Fluorobenzyl)-1*H*-1,2,3-triazol-4-yl)methoxy)phenyl)-2*H*-chromen-2-one (8d)

Yield 83%; white solid; mp 144–145°C; IR (KBr) 3071 (CH aromatic), 1714 (C=O), 1608 (C=C), 1453 (N=N) cm^−1^; ^1^H NMR (500 MHz, CDCl_3_) *δ* = 7.76 (s, 1H, H_4_ coumarin), 7.66 (d, *J* = 8.0 Hz, 2H), 7.55 (s, 1H, triazole), 7.53-7.51 (m, *2*H), 7.35 (d, *J* = 8.5 Hz, 1H), 7.30-7.27 (m, 3H), 7.08-7.05 (m, 2H), 7.03 (d, *J* = 8.0 Hz, 2H), 5.50 (s, 2H, OCH_2_), 5.24 (s, 2H, NCH_2_). ^13^C NMR (125 MHz, CDCl_3_) *δ* = 162.2 (d, *J* = 250.0 Hz, CF), 160.8 (C=O), 158.7 (C-O), 153.4 (C-O), 144.5 (C-N), 138.8 (CH, Ar), 138.7 (C, Ar), 131.1 (CH, Ar), 130.1 (CH, Ar), 130.0 (C, Ar), 129.9 (CH, Ar), 127.7 (CH, Ar), 124.5 (CH, Ar), 122.5 (C, Ar), 122.4 (CH, Ar), 119.8 (C, Ar), 116.4 (CH, Ar), 116.2 (d, *J* = 21.0 Hz, CH, Ar), 114.8 (CH, Ar), 62.1 (CH_2_O), 53.5 (CH_2_N). Anal. Calcd for C_25_H_18_FN_3_O_3_: C, 70.25; H, 4.24; N, 9.83. Found: C, 70.29; H, 4.26; N, 9.85.

##### 3.1.2.5 3-(4-((1-(2-Chlorobenzyl)-1*H*-1,2,3-triazol-4-yl)methoxy)phenyl)-2*H*-chromen-2-one (8e)

Yield 86%; white solid; mp 169–170°C; IR (KBr) 3071 (CH aromatic), 1720 (C=O), 1609 (C=C), 1454 (N=N) cm^−1^; ^1^H NMR (500 MHz, CDCl_3_) *δ* = 7.76 (s, 1H, H_4_ coumarin), 7.65-7.63 (m, 3H), 7.52-7.51 (m, 2H), 7.43 (d, *J* = 7.5 Hz, 1H), 7.34 (d, *J* = 8.5 Hz, 1H), 7.31-7.27 (m, 3H), 7.21-7.20 (m, 1H), 7.04 (d, *J* = 7.5 Hz, 2H), 5.68 (s, 2H, OCH_2_), 5.25 (s, 2H, NCH_2_). ^13^C NMR (125 MHz, CDCl3) *δ* = 160.7 (C=O), 158.8 (C-O), 153.3 (C-O), 144.3 (C-N), 138.7 (CH, Ar), 138.6 (C, Ar), 132.3 (C, Ar), 131.1 (CH, Ar), 130.4 (CH, Ar), 130.3 (CH, Ar), 130.0 (CH, Ar), 129.9 (CH, Ar), 129.8 (CH, Ar), 127.8 (CH, Ar), 127.7 (C, Ar), 127.6 (CH, Ar), 124.4 (CH, Ar), 123.1 (C, Ar), 123.0 (C, Ar), 119.8 (C, Ar), 116.4 (CH, Ar), 114.8 (CH, Ar), 62.1 (CH_2_O), 51.5 (CH_2_N). EI-MS m/z (%): 443 (M^+^), 352, 290, 238, 210, 178, 125. Anal. Calcd for C_25_H_18_ClN_3_O_3_: C, 67.65; H, 4.09; N, 9.47. Found: C, 67.62; H, 4.12; N, 9.49.

##### 3.1.2.6 3-(4-((1-(4-Chlorobenzyl)-1*H*-1,2,3-triazol-4-yl)methoxy)phenyl)-2*H*-chromen-2-one (8f)

Yield 84%; white solid; mp 150–152°C; IR (KBr) 3071 (CH aromatic), 1714 (C=O), 1609 (C=C), 1454 (N=N) cm^−1^; ^1^H NMR (500 MHz, CDCl_3_) *δ* = 7.76 (s, 1H, H_4_ coumarin), 7.66 (d, *J* = 8.0 Hz, 2H), 7.55 (s, 1H, triazole), 7.53- 7.50 (m, 2H), 7.36 (d, *J* = 7.5 Hz, 2H), 7.29 (d, *J* = 7.5 Hz, 1H), 7.27 (dd, *J* = 7.5, 7.5 Hz, 1H), 7.22 (d, *J* = 8.0 Hz, 2H), 7.03 (d, *J* = 8.5 Hz, 2H), 5.51 (s, 2H, OCH_2_), 5.24 (s, 2H, NCH_2_). ^13^C NMR (100 MHz, DMSO-*d*_*6*_) *δ* = 160.3 (C=O), 158.8 (C-O), 153.2 (C-O), 143.4 (C-N), 139.8 (CH, Ar), 135.5 (CH, Ar), 133.4 (C, Ar), 131.9 (C, Ar), 130.4 (CH, Ar), 130.3 (CH, Ar), 129.3 (CH, Ar), 128.9 (CH, Ar), 127.7 (CH, Ar), 126.8 (C, Ar), 125.3 (C, Ar), 125.1 (C, Ar), 120.1 (CH, Ar), 116.3 (CH, Ar), 115.0 (CH, Ar), 61.6 (CH_2_O), 52.5 (CH_2_N). EI-MS m/z (%): 443 (M^+^), 362, 290, 238, 210, 181, 125. Anal. Calcd for C_25_H_18_ClN_3_O_3_: C, 67.65; H, 4.09; N, 9.47. Found: C, 67.63; H, 4.11; N, 9.45.

##### 3.1.2.7 3-(4-((1-(4-Bromobenzyl)-1*H*-1,2,3-triazol-4-yl)methoxy)phenyl)-2*H*-chromen-2-one (8g)

Yield 85%; white solid; mp 161–163°C; IR (KBr) 3065 (CH aromatic), 1708 (C=O), 1604 (C=C), 1451 (N=N) cm^−1^; ^1^H NMR (500 MHz, CDCl_3_) *δ* = 7.76 (s, 1H, H_4_ coumarin), 7.66 (d, *J* = 8.0 Hz, 2H), 7.55 (s, 1H, triazole), 7.53-7.50 (m, 4H), 7.35 (d, *J* = 8.5 Hz, 1H), 7.27 (dd, *J* = 7.5, 7.0 Hz, 1H), 7.15 (d, *J* = 7.5 Hz, 2H), 7.03 (d, *J* = 8.0 Hz, 2H), 5.49 (s, 2H, OCH_2_), 5.24 (s, 2H, NCH_2_). ^13^C NMR (125 MHz, CDCl_3_) *δ* = 160.7 (C=O), 158.7 (C-O), 153.3 (C-O), 144.6 (C-N), 138.7 (CH, Ar), 138.6 (C, Ar), 133.4 (C, Ar), 132.3 (CH, Ar), 131.1 (CH, Ar), 129.9 (CH, Ar), 129.7 (CH, Ar), 127.7 (CH, Ar), 124.5 (CH, Ar), 123.0 (C, Ar), 122.6 (CH, Ar), 122.5 (C, Ar), 119.8 (C, Ar), 116.4 (CH, Ar), 114.8 (CH, Ar), 62.1 (CH_2_O), 53.6 (CH_2_N). EI-MS m/z (%): 487 (M^+^), 406, 290, 238, 210, 169, 143. Anal. Calcd for C_25_H_18_BrN_3_O_3_: C, 61.49; H, 3.72; N, 8.60. Found: C, 61.46; H, 3.75; N, 8.63.

##### 3.1.2.8 3-(4-((1-(2-Nitrobenzyl)-1*H*-1,2,3-triazol-4-yl)methoxy)phenyl)-2*H*-chromen-2-one (8h)

Yield 87%; white solid; mp 176–178°C; IR (KBr) 3024 (CH aromatic), 1717 (C=O), 1609 (C=C), 1527 (NO_2_), 1453 (N=N), 1330 (NO_2_) cm^−1^; ^1^H NMR (500 MHz, CDCl_3_) *δ* = 8.15 (d, *J* = 8.0 Hz, 1H), 7.80 (s, 1H, H_4_ coumarin), 7.77 (s, 1H, triazole), 7.67 (d, *J* = 8.0 Hz, 2H), 7.63 (dd, *J* = 7.5, 7.0 Hz, 1H), 7.55-7.50 (m, 3H, benzyl), 7.36 (d, *J* = 8.0 Hz, 1H), 7.29 (dd, *J* = 8.0, 7.0 Hz, 1H), 7.12 (d, *J* = 8.0 Hz, 1H), 7.06 (d, *J* = 8.0 Hz, 2H), 5.96 (s, 2H, OCH_2_), 5.29 (s, 2H, NCH_2_). ^13^C NMR (125 MHz, CDCl_3_) *δ* = 160.7 (C=O), 158.7 (C-O), 153.4 (C-O), 150.8 (C, Ar), 144.6 (C-N), 138.8 (CH, Ar), 138.7 (C, Ar), 134.5 (CH, Ar), 131.1 (CH, Ar), 130.6 (CH, Ar), 130.0 (CH, Ar), 127.8 (CH, Ar), 127.7 (CH, Ar), 125.5 (CH, Ar), 125.4 (C, Ar), 124.5 (CH, Ar), 123.9 (C, Ar), 123.8 (C, Ar), 119.8 (CH, Ar), 116.4 (CH, Ar), 114.9 (CH, Ar), 62.1 (CH_2_O), 50.9 (CH_2_N). EI-MS m/z (%): 454 (M^+^), 424, 290, 238, 210, 181, 152. Anal. Calcd for C_25_H_18_N_4_O_5_: C, 66.08; H, 3.99; N, 12.33. Found: C, 66.11; H, 4.02; N, 12.38.

##### 3.1.2.9 3-(4-((1-(3-Nitrobenzyl)-1*H*-1,2,3-triazol-4-yl)methoxy)phenyl)-2*H*-chromen-2-one (8i)

Yield 88%; white solid; mp 168–170°C; IR (KBr) 3046 (CH aromatic), 1713 (C=O), 1609 (C=C), 1532 (NO_2_), 1453 (N=N), 1330 (NO_2_) cm^−1^; ^1^H NMR (500 MHz, DMSO-*d*_*6*_) *δ* = 8.41 (s, 1H, H_4_ coumarin), 8.25 (s, 1H, triazole), 8.24-8.20 (m, 2H), 7.79-7.77 (m, 2H), 7.72 (s, 1H), 7.71-7,70- (m, 2H), 7.61 (dd, *J* = 7.5, 7.0 Hz, 1H), 7.43 (d, *J* = 7.5 Hz, 1H), 7.38 (dd, *J* = 7.0, 7.0 Hz, 1H), 7.13 (d, *J* = 7.5 Hz, 2H), 5.81 (s, 2H, OCH_2_), 5.23 (s, 2H, NCH_2_). ^13^C NMR (125 MHz, DMSO-*d*_*6*_) *δ* = 159.3 (C=O), 157.8 (C-O), 152.2 (C-O), 147.4 (C-N), 142.5 (C, Ar), 138.7 (CH, Ar), 137.5 (C, Ar), 134.2 (CH, Ar), 130.8 (CH, Ar), 129.9 (CH, Ar), 129.3 (CH, Ar), 127.9 (CH, Ar), 126.7 (C, Ar), 125.8 (C, Ar), 124.5 (CH, Ar), 124.0 (CH, Ar), 122.6 (CH, Ar), 122.3 (CH, Ar), 119.1 (C, Ar), 115.3 (CH, Ar), 114.0 (CH, Ar), 60.7 (CH_2_O), 51.3 (CH_2_N). Anal. Calcd for C_25_H_18_N_4_O_5_: C, 66.08; H, 3.99; N, 12.33. Found: C, 66.10; H, 3.97; N, 12.36.

##### 3.1.2.10 3-(4-((1-(4-Nitrobenzyl)-1*H*-1,2,3-triazol-4-yl)methoxy)phenyl)-2*H*-chromen-2-one (8j)

Yield 90%; white solid; mp 203–205°C; IR (KBr) 3042 (CH aromatic), 1691 (C=O), 1608 (C=C), 1513 (NO_2_), 1456 (N=N), 1346 (NO_2_) cm^−1^; ^1^H NMR (500 MHz, DMSO-*d*_*6*_) *δ* = 8.39 (s, 1H, H_4_ coumarin), 8.23 (d, *J* = 8.0 Hz, 2H), 8.20 (s, 1H, triazole), 7.76 (d, *J* = 8.0 Hz, 1H), 7.70 (d, *J* = 8.5 Hz, 2H), 7.60 (dd, *J* = 8.0, 7.5 Hz, 1H), 7.53 (d, *J* = 8.5 Hz, 2H), 7.42 (d, *J* = 8.0 Hz, 1H), 7.36 (dd, *J* = 7.5, 7.5 Hz, 1H), 7.13 (d, *J* = 8.5 Hz, 2H), 5.81 (s, 2H, OCH_2_), 5.23 (s, 2H, NCH_2_). ^13^C NMR (125 MHz, DMSO-*d*_*6*_) *δ* = 160.3 (C=O), 158.8 (C-O), 153.2 (C-O), 147.7 (C-N), 143.9 (CH, Ar), 143.5 (C, Ar), 139.7 (CH, Ar), 131.8 (C, Ar), 130.3 (CH, Ar), 129.5 (CH, Ar), 128.9, (CH, Ar) 127.7 (C, Ar), 126.8 (C, Ar), 125.6 (CH, Ar), 125.0 (CH, Ar), 124.4 (CH, Ar), 120.1 (C, Ar), 116.3 (CH, Ar), 115.0 (CH, Ar), 61.6 (CH_2_O), 52.4 (CH_2_N). Anal. Calcd for C_25_H_18_N_4_O_5_: C, 66.08; H, 3.99; N, 12.33. Found: C, 66.06; H, 3.97; N, 12.36.

##### 3.1.2.11 3-(4-((1-(3-Methylbenzyl)-1*H*-1,2,3-triazol-4-yl)methoxy)phenyl)-2*H*-chromen-2-one (8k)

Yield 91%; white solid; mp 128–130°C; IR (KBr) 3030 (CH aromatic), 1719 (C=O), 1607 (C=C), 1453 (N=N) cm^−1^; ^1^H NMR (500 MHz, CDCl3) *δ* = 7.76 (s, 1H, H_4_ coumarin), 7.66 (d, *J* = 8.0 Hz, 2H), 7.54 (s, 1H, triazole), 7.51-7.49 (m, 2H), 7.35 (d, *J* = 8.0 Hz, 1H), 7.29 (d, *J* = 8.0 Hz, 1H), 7.26 (d, *J* = 8.0 Hz, 1H), 7.17 (d, *J* = 8.0 Hz, 1H), 7.09-7.08 (m, 2H), 7.03 (d, *J* = 8.5 Hz, 2H), 5.49 (s, 2H, OCH_2_), 5.23 (s, 2H, NCH_2_), 2.34 (s, 3H, CH_3_). ^13^C NMR (125 MHz, DMSO-*d*_*6*_) *δ* = 160.3 (C=O), 158.8 (C-O), 153.2 (C-O), 143.3 (C-N), 139.7 (CH, Ar), 138.5 (C, Ar), 136.4 (CH, Ar), 131.8 (C, Ar), 130.3 (CH, Ar), 129.3 (CH, Ar), 129.2 (CH, Ar), 129.0 (CH, Ar), 128.9 (CH, Ar), 127.7 (C, Ar), 126.8 (C, Ar), 125.6 (CH, Ar), 125.2 (CH, Ar), 125.0 (CH, Ar), 120.1 (C, Ar), 116.3 (CH, Ar), 115.0 (CH, Ar), 61.6 (CH_2_O), 53.3 (CH_2_N), 21.4 (CH_3_). EI-MS m/z (%): 423 (M^+^), 342, 290, 238, 210, 181, 158. Anal. Calcd for C_26_H_21_N_3_O_3_: C, 73.74; H, 5.00; N, 9.92. Found: C, 73.77; H, 5.03; N, 9.95.

##### 3.1.2.12 3-(4-((1-(3-Methoxybenzyl)-1*H*-1,2,3-triazol-4-yl)methoxy)phenyl)-2*H*-chromen-2-one (8l)

Yield 83%; white solid; mp 123–125°C; IR (KBr) 3022 (CH aromatic), 1710 (C=O), 1608 (C=C), 1452 (N=N) cm^−1^; ^1^H NMR (500 MHz, CDCl_3_) *δ* = 7.76 (s, 1H, H_4_ coumarin), 7.66 (d, *J* = 7.5 Hz, 2H), 7.56 (s, 1H, triazole), 7.52-7.50 (m, 2H), 7.35 (d, *J* = 7.5 Hz, 1H), 7.29-7.27 (m, 2H), 7.03 (d, *J* = 7.5 Hz, 2H), 6.90-6.87 (m, 2H), 6.80 (s, 1H), 5.50 (s, 2H, OCH_2_), 5.23 (s, 2H, NCH_2_), 3.78 (s, 3H, OCH_3_). ^13^C NMR (125 MHz, CDCl_3_) *δ* = 160.7 (C=O), 160.2 (C-OMe), 158.8 (C-O), 153.3 (C-O), 144.4 (C-N), 138.7 (CH, Ar), 138.6 (C, Ar), 135.9 (CH, Ar), 131.1 (CH, Ar), 130.2 (CH, Ar), 129.9 (CH, Ar), 127.7 (CH, Ar), 124.5 (C, Ar), 122.7 (CH, Ar), 122.6 (C, Ar), 120.3 (CH, Ar), 119.8 (C, Ar), 116.4 (CH, Ar), 114.8 (CH, Ar), 114.3 (CH, Ar), 113.7 (CH, Ar), 62.1 (CH_2_O), 55.3 (CH_3_O), 54.2 (CH_2_N). EI-MS m/z (%): 439 (M^+^), 290, 238, 210, 181, 121. Anal. Calcd for C_26_H_21_N_3_O_4_: C, 71.06; H, 4.82; N, 9.56. Found: C, 71.09; H, 4.80; N, 9.58.

##### 3.1.2.13 3-(4-((1-(4-Methoxybenzyl)-1*H*-1,2,3-triazol-4-yl)methoxy)phenyl)-2*H*-chromen-2-one (8m)

Yield 80%; white solid; mp 132–133°C; IR (KBr) 3065 (CH aromatic), 1714 (C=O), 1610 (C=C), 1452 (N=N) cm^−1^; ^1^H NMR (500 MHz, CDCl_3_) *δ* = 7.74 (s, 1H, H_4_ coumarin), 7.64 (d, *J* = 6.5 Hz, 2H), 7.51-7.49 (m, 3H), 7.33 (d, *J* = 7.0 Hz, 1H), 7.24-7.21 (m, 3H), 7.01 (d, *J* = 6.5 Hz, 2H), 6.89-6.88 (m, 2H), 5.45 (s, 2H, OCH_2_), 5.20 (s, 2H, NCH_2_), 3.79 (s, 3H, OCH_3_). ^13^C NMR (125 MHz, CDCl_3_) *δ* = 160.7 (C=O), 160.0 (C-OMe), 158.7 (C-O), 153.3 (C-O), 144.2 (C-N), 138.7 (CH, Ar), 138.6 (C, Ar), 131.0 (CH, Ar), 129.9 (CH, Ar), 129.7 (CH, Ar), 127.7 (CH, Ar), 127.6 (C, Ar), 126.4 (C, Ar), 124.4 (CH, Ar), 122.4 (CH, Ar), 119.8 (C, Ar), 116.4 (CH, Ar), 114.7 (CH, Ar), 114.5 (CH, Ar), 62.1 (CH_2_O), 55.3 (CH_3_O), 53.8 (CH_2_N). EI-MS m/z (%): 439 (M^+^), 290, 238, 210, 181, 121. Anal. Calcd for C_26_H_21_N_3_O_4_: C, 71.06; H, 4.82; N, 9.56. Found: C, 71.09; H, 4.85; N, 9.53.

##### 3.1.2.14 3-(4-((1-Benzyl-1*H*-1,2,3-triazol-4-yl)methoxy)phenyl)-8-methoxy-2*H*-chromen-2-one (8n)

Yield 82%; white solid; mp 154–155°C; IR (KBr) 3028 (CH aromatic), 1719 (C=O), 1606 (C=C), 1464 (N=N) cm^−1^; ^1^H NMR (500 MHz, CDCl_3_) *δ* = 7.72 (s, 1H, H_4_ coumarin), 7.66 (d, *J* = 8.0 Hz, 2H), 7.55 (s, 1H, triazole), 7.38-7.35 (m, 3H), 7.29-7.27 (m, 2H), 7.20 (dd, *J* = 7.5, 7.5 Hz, 1H), 7.08 (d, *J* = 7.5 Hz, 1H), 7.04 (d, *J* = 7.5 Hz, 1H), 7.01 (d, *J* = 8.0 Hz, 2H), 5.53 (s, 2H, OCH_2_), 5.22 (s, 2H, NCH_2_), 3.97 (s, 3H, OCH_3_). ^13^C NMR (125 MHz, CDCl_3_) *δ* = 160.1 (C=O), 158.7 (C-O), 147.0 (C-O), 144.4 (C-O), 143.0 (C-N), 138.8 (CH, Ar), 138.6 (C, Ar), 134.4 (C, Ar), 129.9 (CH, Ar), 129.1 (CH, Ar), 128.8 (CH, Ar), 128.1 (CH, Ar), 127.9 (C, Ar), 127.6 (C, Ar), 124.3 (CH, Ar), 122.7 (CH, Ar), 120.4 (C, Ar), 119.2 (CH, Ar), 114.7 (CH, Ar), 113.0 (CH, Ar), 62.1 (CH_2_O), 56.2 (CH_3_O), 54.2 (CH_2_N). EI-MS m/z (%): 439 (M^+^), 320, 268, 240, 144, 91. Anal. Calcd for C_26_H_21_N_3_O_4_: C, 71.06; H, 4.82; N, 9.56. Found: C, 71.10; H, 4.80; N, 9.59.

##### 3.1.2.15 3-(4-((1-(2-Fluorobenzyl)-1*H*-1,2,3-triazol-4-yl)methoxy)phenyl)-8-methoxy-2*H*-chromen-2-one (8o)

Yield 82%; white solid; mp 163–165°C; IR (KBr) 3094 (CH aromatic), 1719 (C=O), 1605 (C=C), 1475 (N=N) cm^−1^; ^1^H NMR (500 MHz, DMSO-*d*_*6*_) *δ* = 8.30 (s, 1H, H_4_ coumarin), 8.18 (s, 1H, triazole), 7.71 (d, *J* = 8.5 Hz, 2H), 7.42 (dd, *J* = 14.0, 7.0 Hz, 1H), 7.35 (dd, *J* = 8.0, 7.5 Hz, 1H), 7.31-7.28 (m, 3H), 7.25 (d, *J* = 9.0 Hz, 1H), 7.22 (dd, *J* = 8.0, 7.5 Hz, 1H), 7.12 (d, *J* = 8.5 Hz, 2H), 5.69 (s, 2H, OCH_2_), 5.21 (s, 2H, NCH_2_), 3.92 (s, 3H, OCH_3_).^13^C NMR (125 MHz, DMSO-*d*_*6*_) *δ* = 160.1 (d, *J* = 245.0 Hz, CF), 159.6 (C=O), 158.4 (C-O), 146.2 (C-O), 142.8 (C-O), 142.0 (C-N), 139.5 (CH, Ar), 130.8 (CH, Ar), 130.7 (CH, Ar), 129.8 (CH, Ar), 127.1 (C, Ar), 126.4 (CH, Ar), 125.0 (C, Ar), 124.9 (CH, Ar), 124.5 (CH, Ar), 122.8 (d, *J* = 14.5 Hz, C, Ar), 120.2 (CH, Ar), 119.7 (CH, Ar), 115.6 (d, *J* = 21.0 Hz, CH, Ar), 114.4 (CH, Ar), 113.6 (C, Ar), 61.1 (CH_2_O), 56.4 (CH_3_O), 47.0 (CH_2_N). EI-MS m/z (%): 457 (M^+^), 320, 268, 240, 162, 109. Anal. Calcd for C_26_H_20_FN_3_O_4_: C, 68.26; H, 4.41; N, 9.19. Found: C, 68.23; H, 4.44; N, 9.21.

##### 3.1.2.16 3-(4-((1-(3-Fluorobenzyl)-1*H*-1,2,3-triazol-4-yl)methoxy)phenyl)-8-methoxy-2*H*-chromen-2-one (8p)

Yield 85%; white solid; mp 162–163°C; IR (KBr) 3032 (CH aromatic), 1719 (C=O), 1605 (C=C), 1475 (N=N) cm^−1^; ^1^H NMR (500 MHz, CDCl_3_) *δ* = 7.73 (s, 1H, H_4_ coumarin), 7.66 (d, *J* = 8.0 Hz, 2H), 7.58 (s, 1H, triazole), 7.35 (dd, *J* = 14.0, 7.5 Hz, 1H), 7.21 (dd, *J* = 8.0, 7.5 Hz, 1H), 7.09 (d, *J* = 8.0 Hz, 1H), 7.06-7.04 (m, 3H), 7.01 (d, *J* = 8.0 Hz, 2H), 6.97 (d, *J* = 9.0 Hz, 1H), 5.53 (s, 2H, OCH_2_), 5.24 (s, 2H, NCH_2_), 3.97 (s, 3H, OCH_3_). ^13^C NMR (125 MHz, CDCl_3_) *δ* = 163.0 (d, *J* = 250.0 Hz, CF), 160.1 (C=O), 158.7 (C-O), 147.0 (C-O), 144.6 (C-N), 138.8 (CH, Ar), 138.7 (C, Ar), 130.9 (d, *J* = 15.0 Hz, C, Ar), 129.9 (CH, Ar), 127.7 (C, Ar), 127.6 (CH, Ar), 124.3 (CH, Ar), 123.6 (CH, Ar), 122.7 (CH, Ar), 122.6 (C, Ar), 120.4 (C, Ar), 119.2 (CH, Ar), 115.8 (d, *J* = 22.0 Hz, CH, Ar), 115.1 (d, *J* = 22.0 Hz, CH, Ar), 114.8 (CH, Ar), 113.0 (CH, Ar), 62.1 (CH_2_O), 56.2 (CH_3_O), 53.6 (CH_2_N). EI-MS m/z (%): 457 (M^+^), 320, 268, 240, 162, 109. Anal. Calcd for C_26_H_20_FN_3_O_4_: C, 68.26; H, 4.41; N, 9.19. Found: C, 68.28; H, 4.43; N, 9.17.

##### 3.1.2.17 3-(4-((1-(4-Fluorobenzyl)-1*H*-1,2,3-triazol-4-yl)methoxy)phenyl)-8-methoxy-2*H*-chromen-2-one (8q)

Yield 80%; white solid; mp 156–158°C; IR (KBr) 3046 (CH aromatic), 1713 (C=O), 1606 (C=C), 1462 (N=N) cm^−1^; ^1^H NMR (500 MHz, CDCl_3_) *δ* = 7.73 (s, 1H, H_4_ coumarin), 7.66 (d, *J* = 7.0 Hz, 2H), 7.54 (s, 1H, triazole), 7.27 (d, *J* = 7.0 Hz, 2H), 7.21-7.19 (m, 1H), 7.10-7.07 (m, 2H), 7.06-7.05 (m, 2H), 7.01 (d, *J* = 7.0 Hz, 2H), 5.51 (s, 2H, OCH_2_), 5.24 (s, 2H, NCH_2_), 3.98 (s, 3H, OCH_3_). ^13^C NMR (125 MHz, CDCl_3_) *δ* = 162.0 (d, *J* = 250.0 Hz, CF), 160.2 (C-OMe), 158.7 (C-O), 147.0 (C-O), 144.6 (C-N), 138.9 (CH, Ar), 138.7 (C, Ar), 130.8 (C, Ar), 130.1 (CH, Ar), 129.9 (CH, Ar), 127.9 (C, Ar), 127.7 (C, Ar), 124.3 (CH, Ar), 122.5 (CH, Ar), 120.4 (C, Ar), 119.2 (CH, Ar), 116.1 (d, *J* = 21.0 Hz, CH, Ar), 114.8 (CH, Ar), 113.0 (CH, Ar), 62.1 (CH_2_O), 56.3 (CH_3_O), 53.5 (CH_2_N). Anal. Calcd for C_26_H_20_FN_3_O_4_: C, 68.26; H, 4.41; N, 9.19. Found: C, 68.24; H, 4.39; N, 9.21.

##### 3.1.2.18 3-(4-((1-(4-Chlorobenzyl)-1*H*-1,2,3-triazol-4-yl)methoxy)phenyl)-8-methoxy-2*H*-chromen-2-one (8r)

Yield 83%; white solid; mp 180–182°C; IR (KBr) 3052 (CH aromatic), 1713 (C=O), 1607 (C=C), 1481 (N=N) cm^−1^; ^1^H NMR (500 MHz, CDCl_3_) *δ* = 7.73 (s, 1H, H_4_ coumarin), 7.66 (d, *J* = 7.5 Hz, 2H), 7.56 (s, 1H, triazole), 7.34 (d, *J* = 8.0 Hz, 2H), 7.23-7.19 (m, 3H), 7.09 (d, *J* = 7.5 Hz, 1H), 7.05 (d, *J* = 7.5 Hz, 1H), 7.01 (d, *J* = 8.0 Hz, 2H), 5.50 (s, 2H, OCH_2_), 5.23 (s, 2H, NCH_2_), 3.97 (s, 3H, OCH_3_). ^13^C NMR (125 MHz, CDCl_3_) *δ* = 160.2 (C=O), 158.7 (C-O), 147.0 (C-O), 144.6 (C-N), 138.8 (CH, Ar), 138.7 (C, Ar), 134.9 (C, Ar), 133.0 (C, Ar), 129.9 (CH, Ar), 129.5 (CH, Ar), 129.4 (CH, Ar), 127.9 (C, Ar), 127.7 (C, Ar), 124.3 (CH, Ar), 122.6 (CH, Ar), 120.4 (C, Ar), 119.2 (CH, Ar), 114.7 (CH, Ar), 113.0 (CH, Ar), 62.1 (CH_2_O), 56.2 (CH_3_O), 53.5 (CH_2_N). EI-MS m/z (%): 473 (M^+^), 320, 268, 168, 139, 125. Anal. Calcd for C_26_H_20_ClN_3_O_4_: C, 65.89; H, 4.25; N, 8.87. Found: C, 65.91; H, 4.27; N, 8.85.

##### 3.1.2.19 8-Methoxy-3-(4-((1-(2-nitrobenzyl)-1*H*-1,2,3-triazol-4-yl)methoxy)phenyl)-2*H*-chromen-2-one (8s)

Yield 78%; white solid; mp 158–159°C; IR (KBr) 3043 (CH aromatic), 1710 (C=O), 1607 (C=C), 1472 (N=N) cm^−1^; ^1^H NMR (500 MHz, CDCl_3_) *δ* = 8.14 (d, *J* = 8.0 Hz, 1H), 7.80 (s, 1H, H_4_ coumarin), 7.74 (s, 1H, triazole), 7.68 (d, *J* = 8.0 Hz, 2H), 7.62 (dd, *J* = 7.5, 7.0 Hz, 1H), 7.3 (dd, *J* = 7.5, 7.0 Hz, 1H), 7.21 (dd, *J* = 7.5, 7.5 Hz, 1H), 7.10 (d, *J* = 8.0 Hz, 2H), 7.07-7.04 (m, 3H), 5.95 (s, 2H, OCH_2_), 5.29 (s, 2H, NCH_2_), 3.98 (s, 3H, OCH_3_). ^13^C NMR (125 MHz, CDCl_3_) *δ* = 160.1 (C=O), 158.6 (C-O), 147.0 (C-O), 144.6 (C-N), 138.8 (C, Ar), 138.7 (CH, Ar), 134.5 (CH, Ar), 130.5 (CH, Ar), 129.9 (CH, Ar), 129.8 (CH, Ar), 127.9 (C, Ar), 127.8 (C, Ar), 125.5 (C, Ar), 125.4 (CH, Ar), 124.3 (CH, Ar), 123.9 (CH, Ar), 123.8 (C, Ar), 120.4 (C, Ar), 119.2 (CH, Ar), 114.8 (CH, Ar), 113.0 (CH, Ar), 62.1 (CH_2_O), 56.2 (CH_3_O), 50.9 (CH_2_N). EI-MS m/z (%): 484 (M^+^), 320, 268, 240, 197, 169, 139. Anal. Calcd for C_26_H_20_N_4_O_6_: C, 64.46; H, 4.16; N, 11.56. Found: C, 64.43; H, 4.18; N, 11.59.

##### 3.1.2.20 6-Bromo-3-(4-((1-(4-bromobenzyl)-1*H*-1,2,3-triazol-4-yl)methoxy)phenyl)-2*H*-chromen-2-one (8t)

Yield 82%; white solid; mp 207–209°C; IR (KBr) 3049 (CH aromatic), 1715 (C=O), 1605 (C=C), 1478 (N=N) cm^−1^; ^1^H NMR (500 MHz, CDCl_3_) *δ* = 7.66-7.65 (m, 3H), 7.64 (s, 1H, H_4_ coumarin), 7.59 (d, *J* = 9.0 Hz, 1H), 7.55 (s, 1H, triazole), 7.51 (d, *J* = 8.0 Hz, 2H), 7.23 (d, *J* = 9.0 Hz, 1H), 7.15 (d, *J* = 7.5 Hz, 2H), 7.03 (d, *J* = 8.0 Hz, 2H), 5.50 (s, 2H, OCH_2_), 5.24 (s, 2H, NCH_2_). ^13^C NMR (125 MHz, CDCl_3_) *δ* = 160.1 (C=O), 159.0 (C-O), 144.6 (C-O), 138.6 (C-N), 137.1 (C, Ar), 137.0 (C, Ar), 133.8 (C, Ar), 133.5 (CH, Ar), 132.4 (CH, Ar), 130.0 (CH, Ar), 129.8 (CH, Ar), 127.2 (CH, Ar), 123.1 (CH, Ar), 122.7 (CH, Ar), 122.6 (C, Ar), 121.4 (C, Ar), 118.1 (CH, Ar), 117.0 (C, Ar), 114.9 (CH, Ar), 62.1 (CH_2_O), 53.6 (CH_2_N). EI-MS m/z (%): 567 (M^+^), 486, 368, 316, 288, 222, 169. Anal. Calcd for C_25_H_17_Br_2_N_3_O_3_: C, 52.94; H, 3.02; N, 7.41. Found: C, 52.91; H, 3.05; N, 7.44.

##### 3.1.2.21 6-Bromo-3-(4-((1-(2-fluorobenzyl)-1*H*-1,2,3-triazol-4-yl)methoxy)phenyl)-2*H*-chromen-2-one (8u)

Yield 80%; white solid; mp 175–177°C; IR (KBr) 3095 (CH aromatic), 1721 (C=O), 1604 (C=C), 1505 (N=N) cm^−1^; ^1^H NMR (500 MHz, DMSO-*d*_*6*_) *δ* = 8.31 (s, 1H), 8.13 (s, 1H, H_4_ coumarin), 7.97 (s, 1H, triazole), 7.72 (d, *J* = 8.5 Hz, 1H), 7.67 (d, *J* = 8.0 Hz, 2H), 7.41 (d, *J* = 7.0 Hz, 1H), 7.38-7.34 (m, 2H), 7.26 (d, *J* = 9.0 Hz, 1H), 7.22 (dd, *J* = 8.0, 7.0 Hz, 1H), 7.12 (d, *J* = 8.0 Hz, 2H), 5.68 (s, 2H, OCH_2_), 5.20 (s, 2H, NCH_2_). ^13^C NMR (125 MHz, DMSO-*d*_*6*_) *δ* = 160.1 (d, *J* = 245.0 Hz, CF), 159.4 (C=O), 158.6 (C-O), 157.9 (C-O), 151.8 (C-N), 137.8 (CH, Ar), 133.6 (C, Ar), 130.9 (CH, Ar), 130.8 (CH, Ar), 130.3 (C, Ar), 129.9 (CH, Ar), 127.4 (CH, Ar), 126.8 (CH, Ar), 124.9 (C, Ar), 124.8 (CH, Ar), 122.9 (C, Ar), 121.6 (CH, Ar), 118.1 (CBr), 116.1 (CH, Ar), 115.7 (d, *J* = 21.0 Hz, CH, Ar), 114.5 (CH, Ar), 61.1 (CH_2_O), 47.0 (CH_2_N). EI-MS m/z (%): 505 (M^+^), 478, 426, 368, 316, 290, 261, 162, 109. Anal. Calcd for C_25_H_17_BrFN_3_O_3_: C, 59.30; H, 3.38; N, 8.30. Found: C, 59.27; H, 3.40; N, 8.33.

##### 3.1.2.22 3-(4-((1-Benzyl-1*H*-1,2,3-triazol-4-yl)methoxy)phenyl)-6-nitro-2*H*-chromen-2-one (8v)

Yield 82%; white solid; mp 191–193°C; IR (KBr) 3041 (CH aromatic), 1729 (C=O), 1605 (C=C), 1505 (NO_2_), 1481 (N=N), 1257 (NO_2_) cm^−1^; ^1^H NMR (500 MHz, DMSO-*d*_*6*_) *δ* = 8.72 (s, 1H), 8.39-8.37 (m, 2H), 8.33 (s, 1H, triazole), 7.70 (d, *J* = 8.5 Hz, 2H), 7.63 (d, *J* = 9.0 Hz, 1H), 7.38 (t, *J* = 7.0 Hz, 2H), 7.34-7.32 (m, 3H), 7.15 (d, *J* = 8.5 Hz, 2H), 5.62 (s, 2H, OCH_2_), 5.21 (s, 2H, NCH_2_). ^13^C NMR (125 MHz, DMSO-*d*_*6*_) *δ* = 159.0 (C=O), 158.8 (C-O), 156.4 (C-O), 143.6 (C-NO_2_), 142.8 (C-N), 137.9 (C, Ar), 136.0 (CH, Ar), 129.9 (CH, Ar), 128.8 (CH, Ar), 128.2 (CH, Ar), 128.1 (CH, Ar), 128.0 (CH, Ar), 126.5 (CH, Ar), 125.8 (C, Ar), 124.8 (C, Ar), 124.2 (C, Ar), 120.0 (CH, Ar), 117.4 (CH, Ar), 114.6 (CH, Ar), 61.2 (CH_2_O), 52.9 (CH_2_N). EI-MS m/z (%): 454 (M^+^), 373, 335, 283, 209, 144, 91. Anal. Calcd for C_25_H_18_N_4_O_5_: C, 66.08; H, 3.99; N, 12.33. Found: C, 66.11; H, 4.01; N, 12.31.

##### 3.1.2.23 3-(4-((1-(2-Fluorobenzyl)-1*H*-1,2,3-triazol-4-yl)methoxy)phenyl)-6-nitro-2*H*-chromen-2-one (8w)

Yield 83%; white solid; mp 179–181°C; 3052 (CH aromatic), 1724 (C=O), 1607 (C=C), 1520 (NO_2_), 1447 (N=N), 1348 (NO_2_) cm^−1^; ^1^H NMR (500 MHz, DMSO-*d*_*6*_) *δ* = 8.71 (d, *J* = 2.5 Hz, 1H), 8.39 (d, *J* = 2.5 Hz, 1H), 8.37 (s, 1H, H_4_ coumarin), 8.31 (s, 1H, triazole), 7.70 (d, *J* = 8.5 Hz, 2H), 7.62 (d, *J* = 9.0 Hz, 1H), 7.41 (dd, *J* = 14.0, 7.0 Hz, 1H), 7.35 (dd, *J* = 7.5, 7.5 Hz, 1H), 7.28-7.27 (m, 2H), 7.15 (d, *J* = 8.5 Hz, 2H), 5.69 (s, 2H, OCH_2_), 5.21 (s, 2H, NCH_2_). ^13^C NMR (125 MHz, DMSO-*d*_*6*_) *δ* = 160.1 (d, *J* = 245.0 Hz, CF), 159.0 (C=O), 158.8 (C-O), 156.4 (C-O), 143.6 (C-N), 142.7 (CNO_2_), 137.9 (CH, Ar), 130.8 (CH, Ar), 130.7 (CH, Ar), 129.9 (CH, Ar), 128.1 (CH, Ar), 126.5 (C, Ar), 125.8 (C, Ar), 124.9 (CH, Ar), 124.8 (C, Ar), 124.2 (C, Ar), 122.9 (CH, Ar), 120.0 (CH, Ar), 117.4 (CH, Ar), 115.6 (d, *J* = 21.0 Hz, CH, Ar), 114.6 (CH, Ar), 61.1 (CH_2_O), 47.0 (CH_2_N). EI-MS m/z (%): 472 (M^+^), 443, 391, 283, 209, 162, 109. Anal. Calcd for C_25_H_17_FN_4_O_5_: C, 66.56; H, 3.63; N, 11.86. Found: C, 63.53; H, 3.65; N, 11.84.

### 3.2 *In Vitro* Biological Evaluations

#### 3.2.1 Cholinesterase Inhibition Assay

Ellman’s method was utilized to assess anti-AChE/BuChE activity of the synthesized compounds (Ellman et al., 1961). AChE from electrophorus electricus and BuChE from equine serum was employed for Ellman’s assay. Each compound (5.0 mg) was dissolved in 1.0 ml of DMSO. Four different concentrations of the target compounds were tested to find out 20–80% inhibition of AChE and/or BuChE activity. Briefly, a mixture of phosphate buffer (2.0 ml, 0.1 M, pH = 8.0), 60.0 µl of DTNB solution, and 20.0 µl of AChE/BuChE was prepared, and 30.0 µl of the test compounds in different concentrations were then added to the mixture. Next, a solution of the substrate (acetylthiocholine iodide/butyrylthiocholine iodide, 20.0 µl) was added to the mixture after 10 min incubation at 25°C. All experiments were performed in triplicates, and the temperature of all solutions was controlled to be maintained at 25°C. The change in the absorbance wavelength of 412 nm was recorded for 1 min. The blank solution was used to validate the non-enzymatic hydrolysis of the substrate during the assessment. The blank solution was prepared by a mixture of 2.0 ml of buffer, 30.0 μl of DMSO, 60.0 μl of DTNB, and 20.0 μl of substrate. The rate of the enzymatic hydrolysis of the substrate was calculated, and % inhibition of the compounds was determined. The half maximal inhibitory concentration (IC_50_) values were graphically determined from inhibition curves (logarithm of the concentration of the tested compound versus percent of the inhibition).

#### 3.2.2 Soybean 15-LOX Inhibition Assay

Soybean lipoxygenase inhibition assay was conducted according to a previously published report (Abdshahzadeh et al., 2019). A solution of the target compound was prepared in DMSO, and it was diluted with phosphate buffer (0.1 M, pH = 8.0). A solution of linoleic acid in ethanol (134.0 μM) and enzyme solution (167 U/ml at final concentration) were then added, and the final solution was incubated at room temperature. The conversion of linoleic acid to 13-hydroperoxylinoleic acid was monitored by change in the absorbance wavelength of 234 nm using a Unico double-beam spectrophotometer.

#### 3.2.3 Neuroprotection Assay

3-(4,5-Dimethylthiazol-2-yl)-2,5-diphenyl tetrazolium bromide (MTT) assay was considered to determine the cell viability of the rat differentiated PC12 cells (Bozorov et al., 2019). PC12 cell line was obtained from the Iranian Biological Resource Center (IBRC) and seeded in 96-well plates (10,000 cells/well) and incubated at 37 °C in a water-saturated atmosphere of 5% CO_2_. After 24 h, different concentrations of each tested compounds (0.1, 1.0, 5.0, 10.0, 20.0, and 50.0 µM) were exposed to the cells and incubated for 3 h. The cells were then treated with H_2_O_2_ (150.0 μM) for another 2 h. MTT solution (20.0 μl, 5.0 mg/ml) was then added, and the new medium was incubated in a CO_2_ incubator for 4 h. Afterward, the medium was removed, and the produced formazan crystals were solubilized using DMSO (100.0 μl). Finally, the related absorbance was recorded at 570 nm using a Synergy 2 multimode plate reader (Biotek, Winooski, VT, United States). The results were expressed as the percentage of the untreated control cells (PC12 cells in the absence of the tested compound and H_2_O_2_). All the experiments were repeated three times, and mean ± SEM of the obtained results was reported.

#### 3.2.4 Cytotoxicity Investigation

The cytotoxic effect of the selected compounds (**8l** and **8n**) on the PC12 cell line was evaluated by using the MTT-based colorimetric assay. First, the PC12 cell line was cultivated in DMEM culture medium, supplemented with fetal bovine serum (10%). The wells of a 96-well microplate were then seeded withby 10^4^ cells and incubated at 37°C and 5% CO_2_, followed by the insertion of the desired concentration of each tested compound (0.1–50 µM) to the desired wells and further incubation at the mentioned conditions for 24 h. Tacrine was applied as the standard compound at a similar concentration range. Thereafter, the culture medium of each well was replaced by 20 µl MTT solution (5.0 mg/ml) and incubated to form formazan crystals, followed by dissolving them in DMSO (100.0 µl) and recording the related absorbance at 570 nm using a Synergy 2 multimode plate reader (Biotek, Winooski, VT, United States). The viability percent was then calculated according to the control well, where no compound was added to the related well. The mentioned protocol was repeated in triplicates, and the mean ± SEM of the obtained results was reported.

#### 3.2.5. Self-Induced and AChE-Induced Aβ_1-42_ Aggregation

The fluorescence assay based on thioflavin T was used to determine the inhibitory activity of the target compounds against self-induced and AChE-induced aggregation of amyloid β protein 1–40 (Zha et al., 2016). The ThT excitation/emission was measured at 448 nm/490 nm using a multimode plate reader (BMG Labtech Omega FLUOstar). Phosphate-buffered saline (PBS, pH = 7.4) containing 1% ammonium hydroxide was used to dissolve amyloid β_1-40_ (Sigma A1075). Pre-fibrillation was achieved by the incubation of amyloid β protein 1–40 (50.0 μM) for 72 h at 37°C.

Aβ_1-40_ (10.0 µl) ± AChE (0.01 u/ml) was added to 0.05 M of potassium phosphate buffer (pH = 7.4), and the obtained solution was incubated at 37°C for 48 h in the absence and presence of the compounds (10.0 μM). An amount of 50.0 μl of thioflavin T (200.0 μM) in glycine–NaOH buffer (50.0 μM, pH = 8.5) was then added to the latter incubated mixture (100.0 µl). Donepezil (10.0 μM) was selected as the reference compound. Self-/AChE-induced aggregation percentages after addition of the tested compounds were determined by the following calculation:

[(IFi/IFo) × 100], where IFi is the fluorescence intensities of the Aβ ± AChE in the presence of the tested compound and IFo is related to the fluorescence intensities of the Aβ ± AChE in the absence of the inhibitors (Bartolini et al., 2003).

#### 3.2.6. Hydrogen Peroxide Cell–Based Assay

The BV-2 immortalized murine microglial cell line was cultured in a high-glucose Dulbecco’s modified Eagle medium (DMEM) without phenol red supplemented with 10% of FBS, %1 of *l* -glutamine, and 1% of penicillin–streptomycin solution. The cells were kept in a moistened atmosphere at 37°C. Quantitation of the extracellular H_2_O_2_ produced by the BV-2 cells was detected using the Oxiselect Sta-343 hydrogen peroxide assay according to the kit’s protocol. Briefly, the cells were seeded in a 384-well plate (1,000 cells/well) overnight in 50.0 μl/well of serum and phenol red free culture medium, then treated with 10.0 μM of the compounds for 3 h, prior to amyloid β_1-40_ treatment (5.0 μM) and incubated for 24 h. An amount of 25.0 μl of medium was collected and mixed with 250.0 μl aqueous working solution. The absorbance values at 595 nm of each well were measured using a BMG Labtech Omega FLUOstar microplate reader. BHT (butylated hydroxytoluene, Cell biolabs) was also tested as a reference antioxidant agent (Datki et al., 2003).

#### 3.2.7 Molecular Docking Studies

Molecular docking was done using Autodock Vina 1.1.2 to predict the binding mode of the compound with the enzyme (Trott and Olson, 2010). For docking studies, the pdb structure of human BuChE (4BDS) in complex with tacrine and the pdb structure of soybean LOX (1JNQ) in complex with Epigallocatechin were retrieved from RCSB protein databank (http://www.rcsb.org). Afterward, the polar hydrogens were added to the receptor, all water molecules and the ligands were omitted from the pdb structure, and pdbqt format of the protein structure was created using Autodock Tools 4.2.6 (Sanner, 1999). The ligand 2D structures were prepared using Marvine-Sketch 15.12.7, 2015, ChemAxon (http://www.chemaxon.com). Then the compounds were converted to pdbqt format by Open babel 2.3.1 (O’Boyle et al., 2011). The active site was defined as a box with dimensions of 15 ×15, 15 Å. The exhaustiveness parameter was set to 80, and the grid box center was set to the dimensions of x = 133.68, y = 115.23, z = 40.89 for BuChE and x = −5.56, y = 141.77, z = 48.01 for LOX. Finally, the lowest energy conformations between ligand and enzyme complexes were chosen for analyzing the interactions. The results were visualized using Chimera 1.12 (Pettersen et al., 2004).

#### 3.2.8 Statistical Analysis

Mean values ±SEM was used to express the results. Data analysis was done using GraphPad Prism software using one-way ANOVA (Dunnet test), and the results were considered statistically significant, when *p*-values were less than 0.05.

## 4 Conclusion

In this work, we aimed to make a balance between the biological activities of the target compounds to reach to the MTDL, even having mild activity against one or several targets instead of finding one-target compounds with high affinity. In conclusion, a novel series of benzyl triazole–arylcoumarin hybrids were synthesized as multi-target–directed ligands (MTDLs) against AD. *In vitro* ChEs inhibition assay revealed that 3-arylcoumarins are more active against BuChE than AChE, and the type of substituent at the coumarin ring had a great effect on the BuChE inhibition activity. Compounds **8r** and **8v** bearing 8-methoxy and 6-nitro substituents on the coumarin ring were the best BuChE inhibitors. LOX inhibition assay showed that the type and position of the substituent on the *N*-benzyl triazole moiety can control the LOX inhibition activity of the target compounds. 2F-Substituted phenyl derivatives (compounds **8b** and **8o**) were the most potent compounds against LOX, superior to quercetin. Substituents on the coumarin ring diminished the inhibitory activity. Neuroprotection assay confirmed that compounds **8l** and **8n** have a remarkable neuroprotective effect on the PC12 cell model injured by H_2_O_2_, significantly greater than quercetin. Compounds **8l** and **8n** as the best MTDLs showed significant inhibitory effect against self-induced Aβ aggregation, more active than the reference drug. Interestingly, compound **8l** could remarkably reduce amyloid-induced peroxide levels in the BV-2 cells. Overall, new triazole–coumarin conjugates (**8l** and **8n)** were presented in this study, having acceptable LOX inhibition activity, good anti-BuChE potency, valuable neuroprotection, remarkable anti-Aβ aggregation, and significant antioxidant activity as potential MTDLs against AD. *In vivo* studies on the anti-AD capability of the selected compounds would be the objective of the future works.

## Data Availability

The original contributions presented in the study are included in the article/Supplementary Material; further inquiries can be directed to the corresponding authors.
