# Supplementary material for: Synthesis of New 3-Arylcoumarins Bearing N-Benzyl Triazole Moiety: Dual Lipoxygenase and Butyrylcholinesterase Inhibitors With Anti-Amyloid Aggregation and Neuroprotective Properties Against Alzheimer’s Disease
Source: Front Chem. 2022 Jan 20;9:810233. doi: 10.3389/fchem.2021.810233 (PMC8812461; doi:10.3389/fchem.2021.810233)
Supplement: Supplementary file 1 [file DataSheet1.PDF]

Supporting Information

Synthesis of New 3-Arylcoumarins Bearing *N*-benzyl Triazole Moiety: Dual Lipoxygenase and Butyrylcholinesterase Inhibitors with Anti-amyloid Aggregation and Neuroprotective Properties Against Alzheimer's Disease

Ladan Pourabdi[1], Tuba Tüylü Küçükkılınç[2], Fatemeh Khoshtale[1], Beyza Ayazgök[2], Hamid Nadri[3], Farid Farokhi Alashti[1], Hamid Forootanfar[4], Tayebeh Akbari[5], Mohammad Shafiei[6], Alireza Foroumadi[6,7], Mohammad Sharifzadeh[8], Mehdi Shafiee Ardestani[9], M. Saeed Abaee[1], Loghman Firoozpour[6], Mehdi Khoobi[7,9*], and Mohammad M. Mojtahedi[1*]

[1]Department of Organic Chemistry and Natural Products, Chemistry and Chemical Engineering Research Center of Iran, Pajoohesh Blvd., 17th km of Tehran Karaj Highway, Tehran 14335-186, Iran

[2]Hacettepe University, Faculty of Pharmacy, Department of Biochemistry, Ankara 06100, Turkey

[3]Department of Medicinal Chemistry, Faculty of Pharmacy and Pharmaceutical Sciences Research Center, Shahid Sadoughi University of Medical Sciences, Yazd 37240171-035, Iran

[4]Pharmaceutical Sciences and Cosmetic Products Research Center, Kerman University of Medical Sciences, Kerman 7616913555, Iran

[5]Department of Microbiology, Islamic Azad University, North Tehran Branch, Tehran, Iran

[6]Department of Medicinal Chemistry, Faculty of Pharmacy, Tehran University of Medical Sciences, Tehran 14176, Iran

[7]Department of Pharmacology and Toxicology, Faculty of Pharmacy, Toxicology and Poisoning Research Centre, Tehran University of Medical Sciences, Tehran, Iran

[8]Pharmaceutical Sciences Research Center, The institute of Pharmaceutical Sciences (TIPS), Tehran University of Medical Sciences, Tehran 1417614411, Iran

[9]Department of Radiopharmacy, Faculty of Pharmacy, Tehran University of Medical Sciences, Tehran, Iran

---

[*]Corresponding authors: Mehdi Khoobi and Mohammad M. Mojtahedi

Tel/fax.: +98-21-64121510, +98-21-44787720; *E-mail address*: mehdi.khoobi@gmail.com; mojtahedi@ccerci.ac.ir.

Table 1

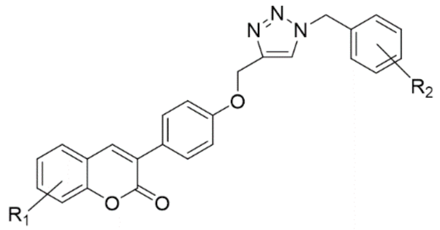

Comp.	R_1	R_2	*ee* AChE[a]	
			%inhib.[b]	IC_{50} μM
**8a**	H	H	23.6	-
**8b**	H	2-F	11.2	-
**8c**	H	3-F	15.3	-
**8d**	H	4-F	32.4	-
**8e**	H	2-Cl	37.3	-
**8f**	H	4-Cl	41.4	-
**8g**	H	4-Br	35.5	-
**8h**	H	2-NO_2	20.8	-
**8i**	H	3-NO_2	49.9	-
**8j**	H	4-NO_2	14.7	-
**8k**	H	3-Me	27.4	-
**8l**	H	3-OMe	16.5	-
**8m**	H	4-OMe	26.6	-
**8n**	8-OMe	H	49.9	-
**8o**	8-OMe	2-F	4.0	-
**8p**	8-OMe	3-F	29.8	-
**8q**	8-OMe	4-F	38.1	-
**8r**	8-OMe	4-Cl	41.5	-
**8s**	8-OMe	2-NO2	27.4	-
**8t**	6-Br	4-Br	15.1	-
**8u**	6-Br	2-F	45.1	-
**8v**	6-NO_2	H	48.3	-
**8w**	6-NO_2	2-F	16.6	-
Tacrine	-	-	-	0.041 ± 0.001

[a]AChE (from electrophorus electricus) was applied in this study

[b]Inhibitor concentration required for 50% inactivation (mean ± SEM of three experiments).

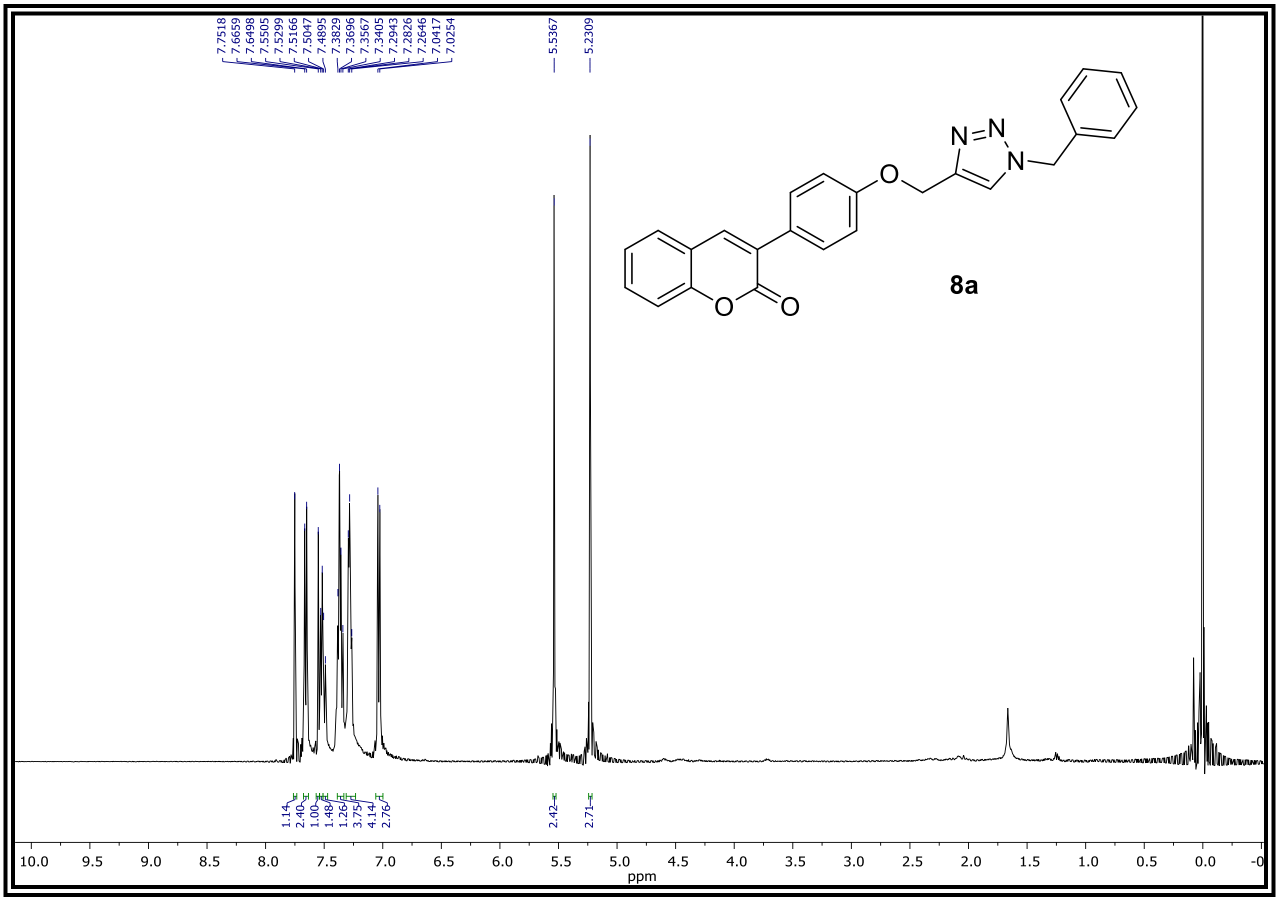

^{1}H NMR (500 MHz, $CDCl_3$) spectrum of 3-(4-((1-Benzyl-1*H*-1,2,3-triazol-4-yl)methoxy)phenyl)-2*H*-chromen-2-one

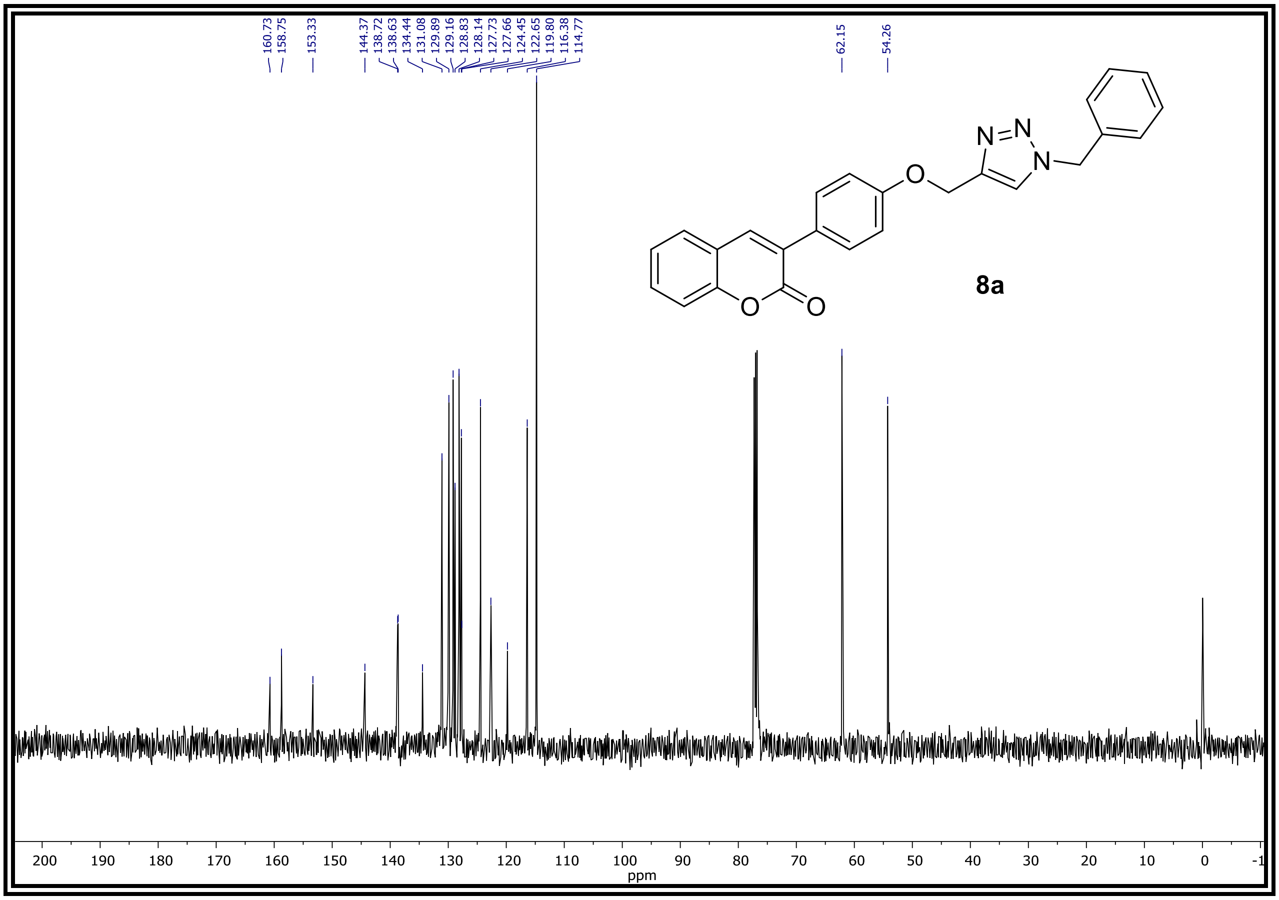

^{13}C NMR (125 MHz, $CDCl_3$) spectrum of 3-(4-((1-Benzyl-1*H*-1,2,3-triazol-4-yl)methoxy)phenyl)-2*H*-chromen-2-one

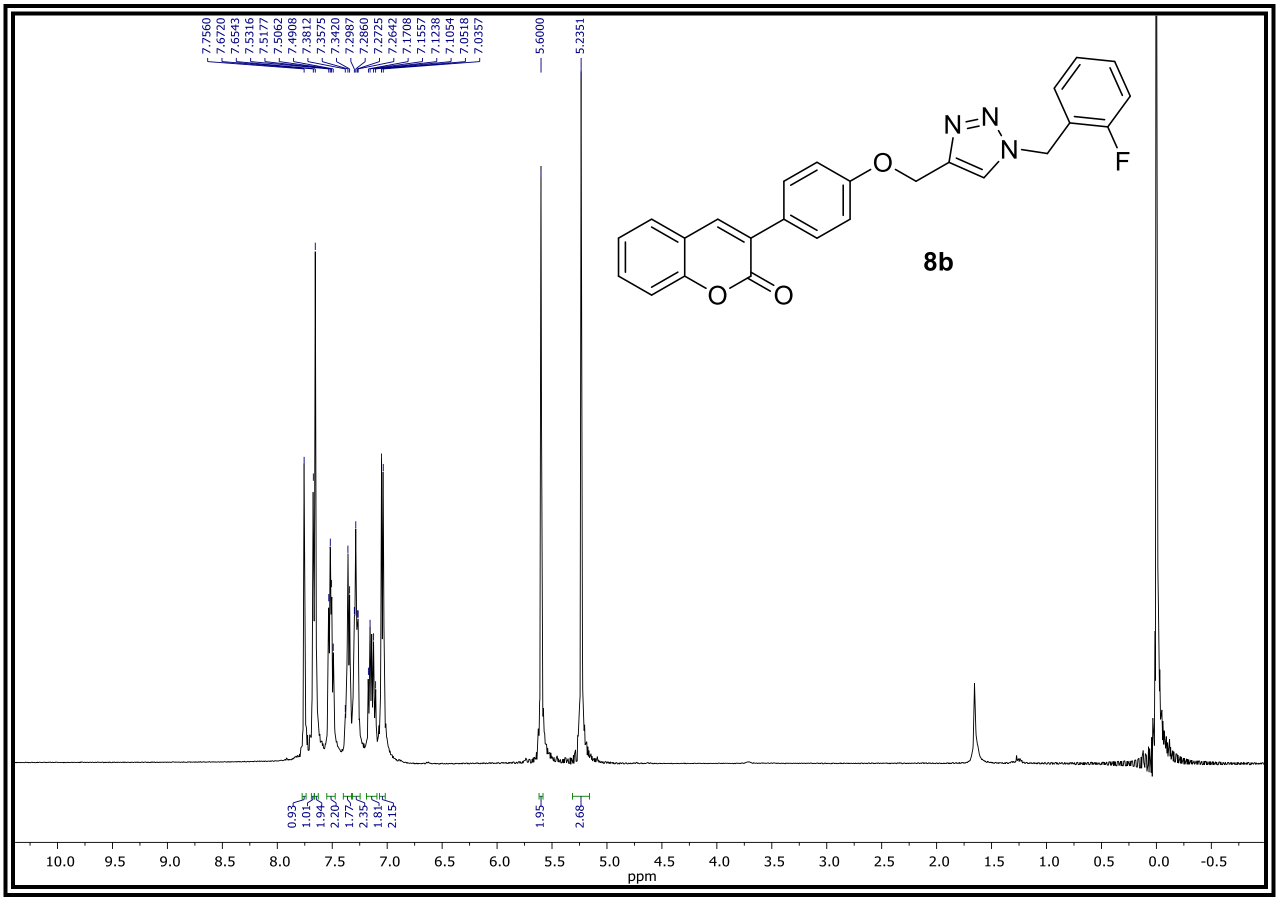

^{1}H NMR (500 MHz, $CDCl_3$) spectrum of 3-(4-((1-(2-Fluorobenzyl)-1*H*-1,2,3-triazol-4-yl)methoxy)phenyl)-2*H*-chromen-2-one

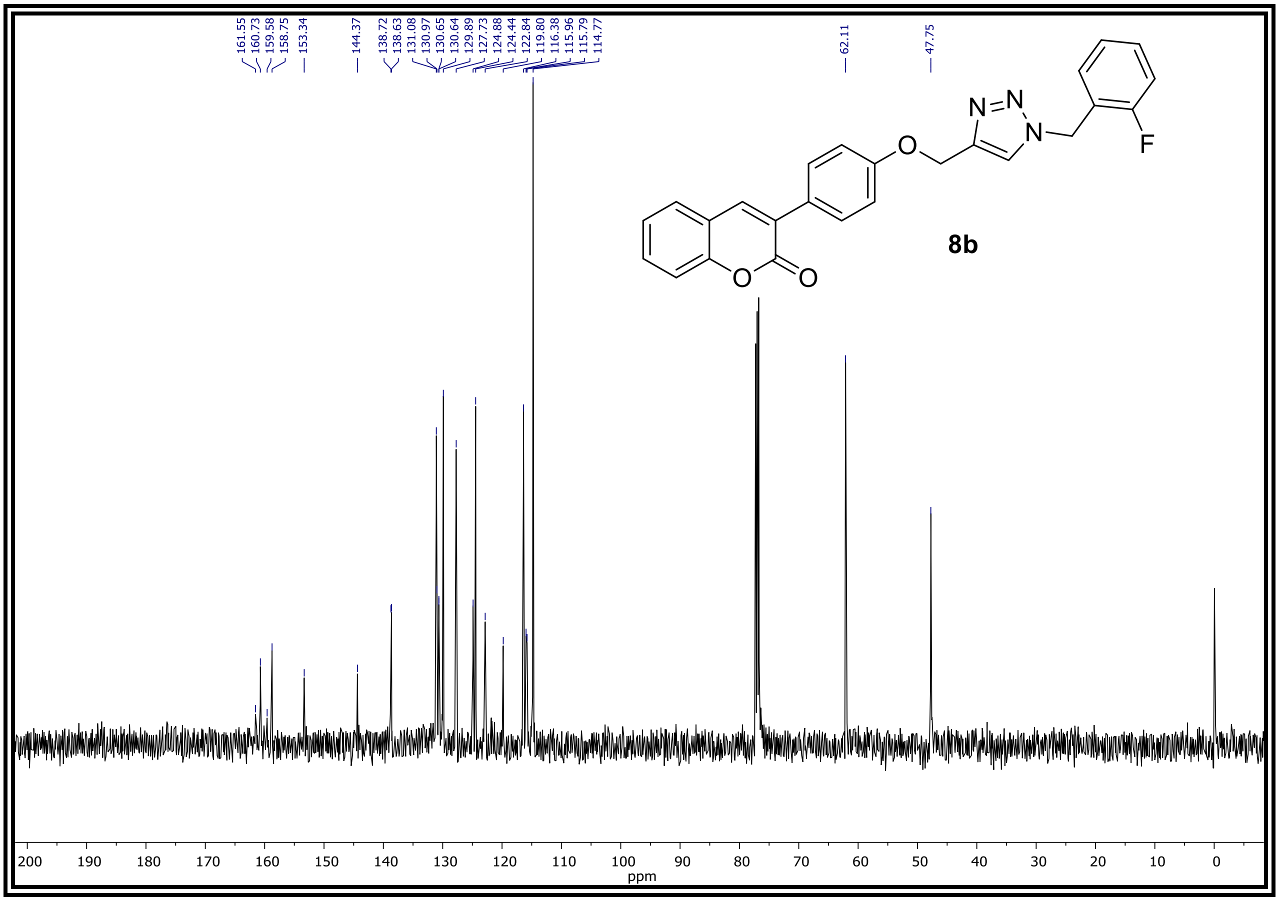

^{13}C NMR (125 MHz, $CDCl_3$) spectrum of 3-(4-((1-(2-Fluorobenzyl)-1*H*-1,2,3-triazol-4-yl)methoxy)phenyl)-2*H*-chromen-2-one

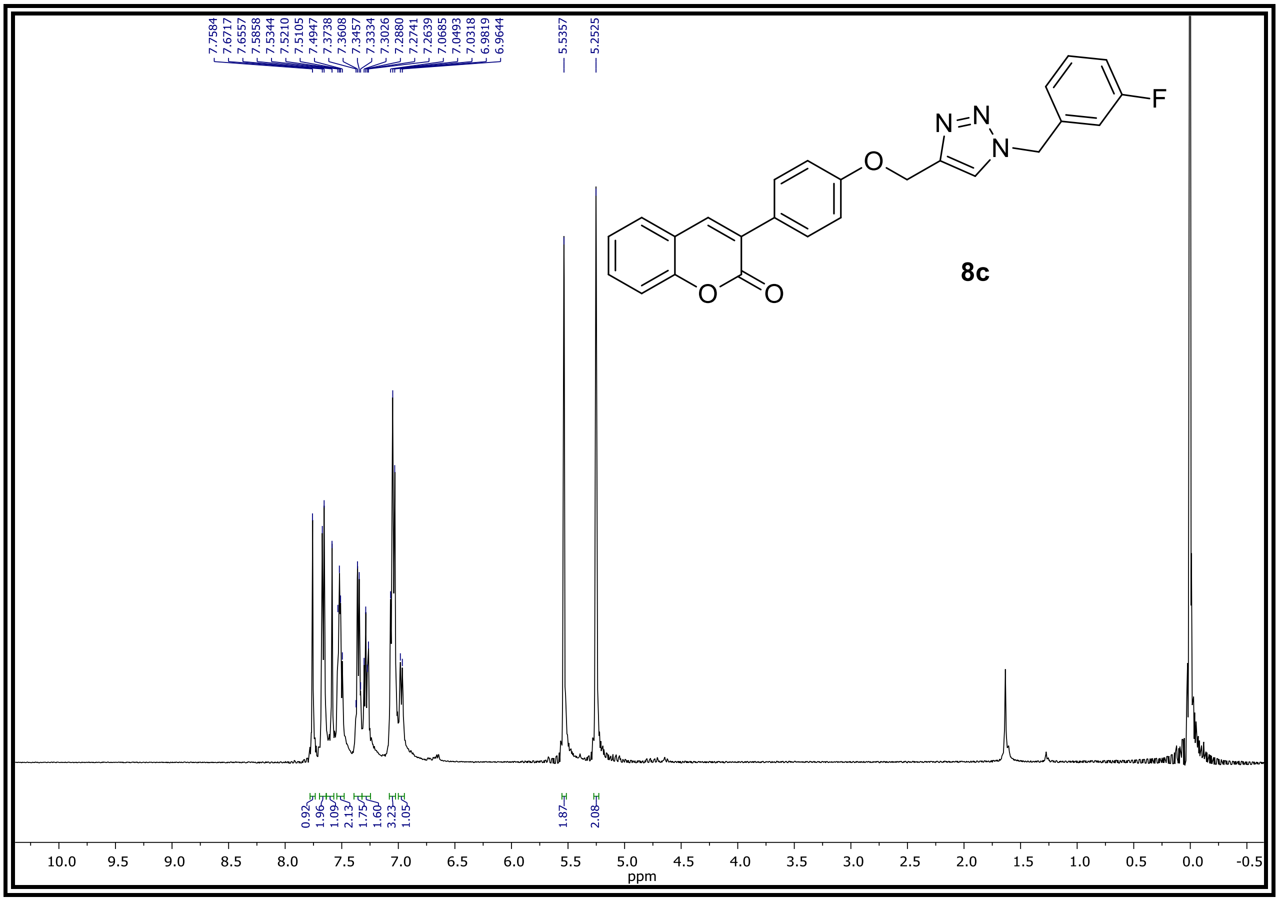

^{1}H NMR (500 MHz, $CDCl_3$) spectrum of 3-(4-((1-(3-Fluorobenzyl)-1*H*-1,2,3-triazol-4-yl)methoxy)phenyl)-2*H*-chromen-2-one

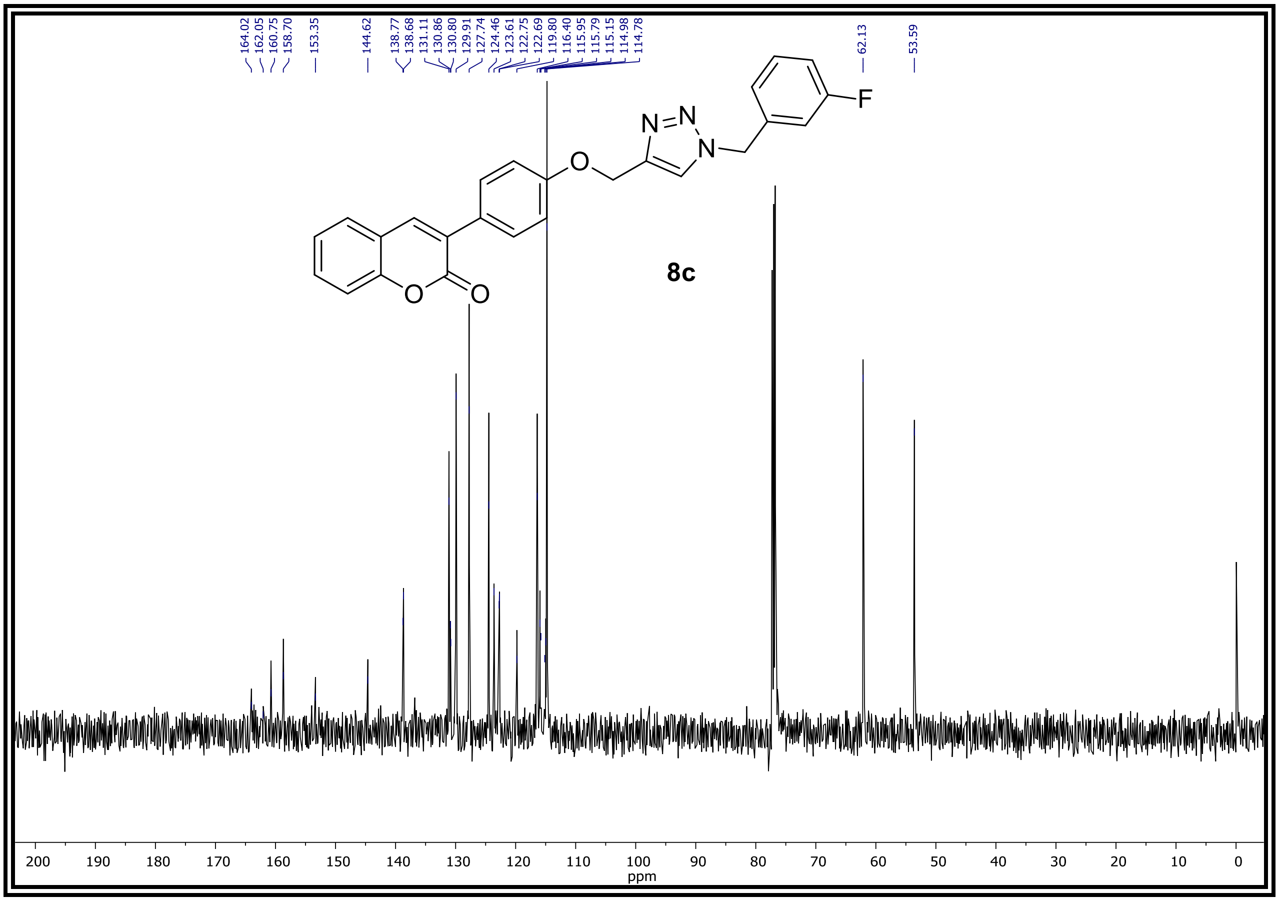

^{13}C NMR (125 MHz, $CDCl_3$) spectrum of 3-(4-((1-(3-Fluorobenzyl)-1*H*-1,2,3-triazol-4-yl)methoxy)phenyl)-2*H*-chromen-2-one

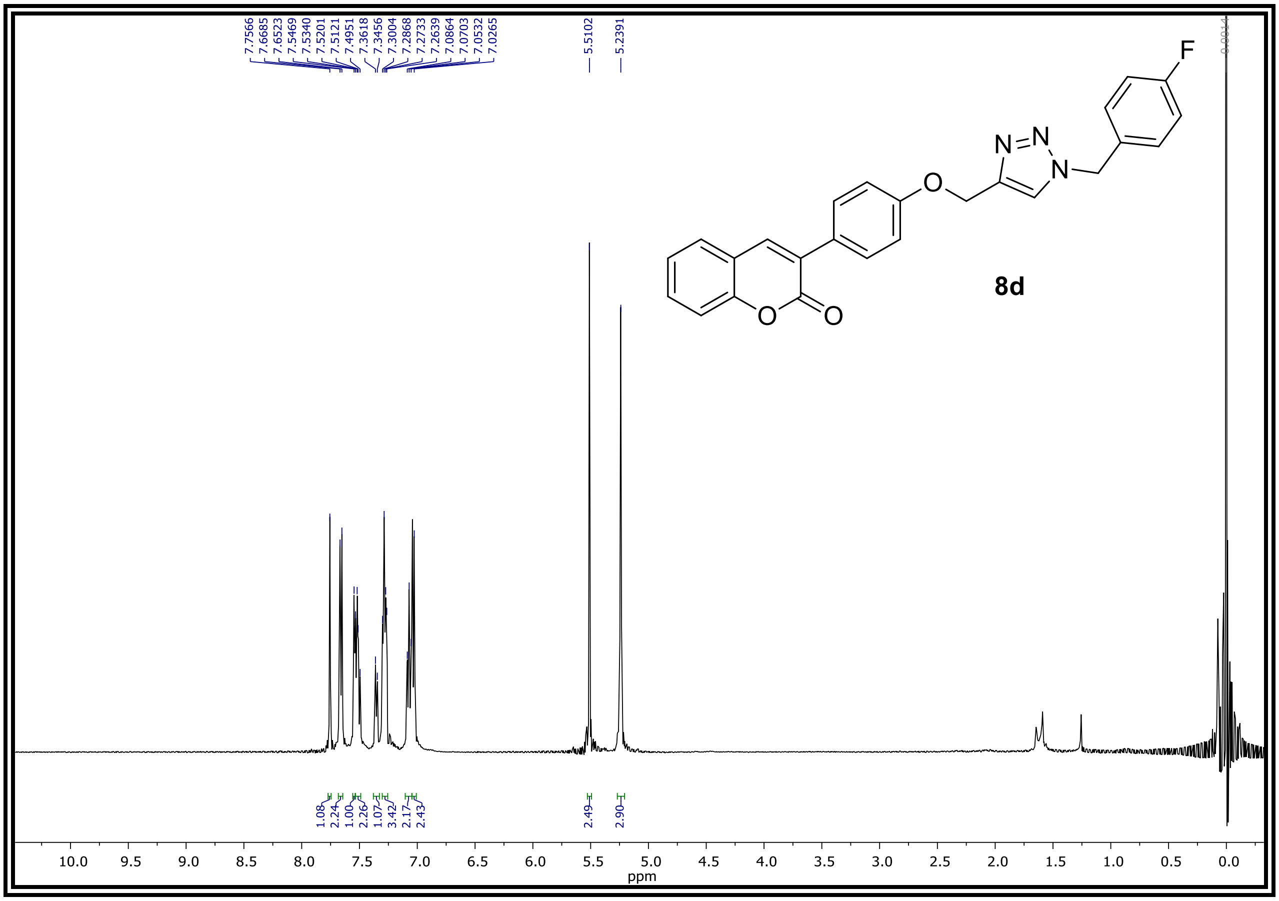

^{1}H NMR (500 MHz, $CDCl_3$) spectrum of 3-(4-((1-(4-Fluorobenzyl)-1*H*-1,2,3-triazol-4-yl)methoxy)phenyl)-2*H*-chromen-2-one

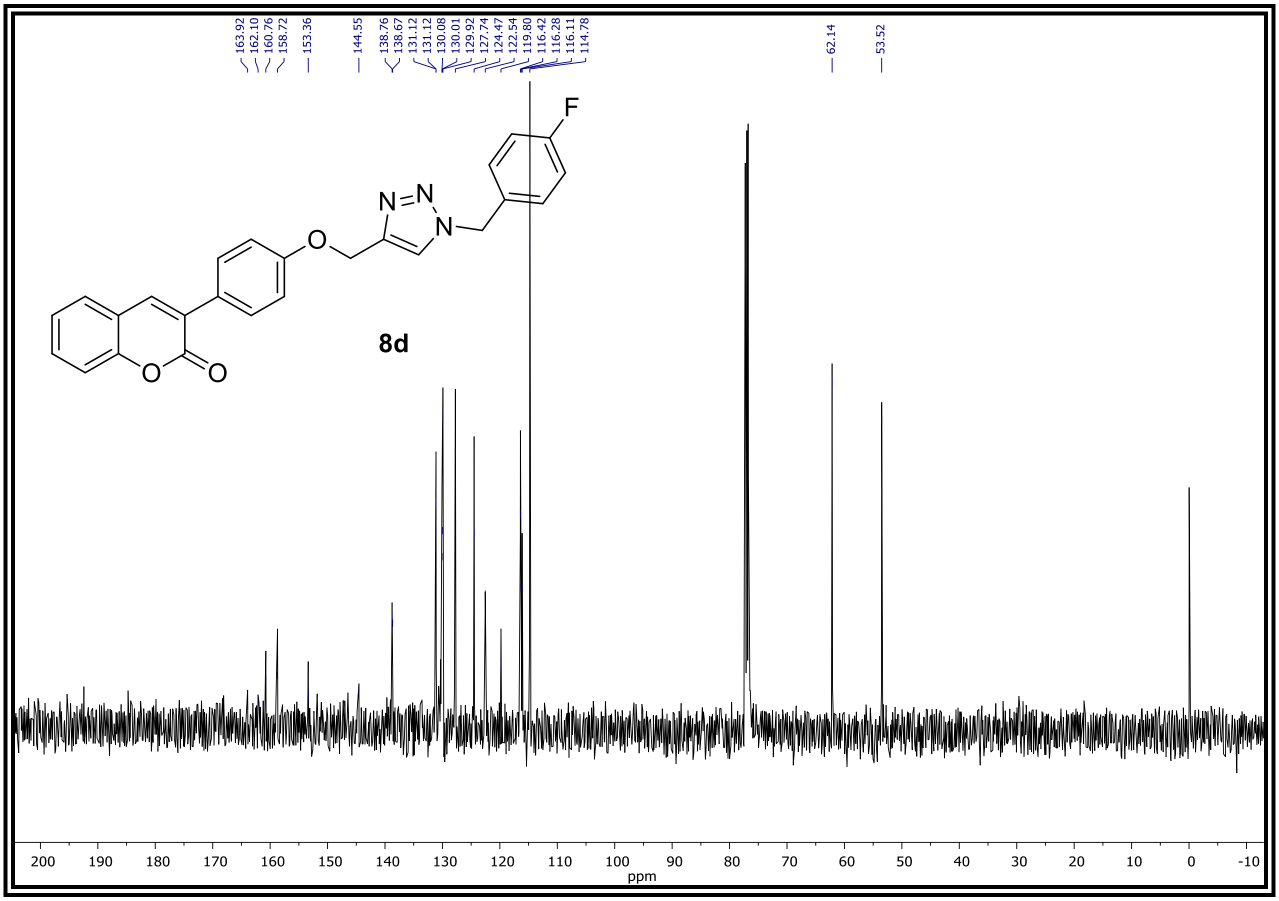

^{13}C NMR (125 MHz, $CDCl_3$) spectrum of 3-(4-((1-(4-Fluorobenzyl)-1*H*-1,2,3-triazol-4-yl)methoxy)phenyl)-2*H*-chromen-2-one

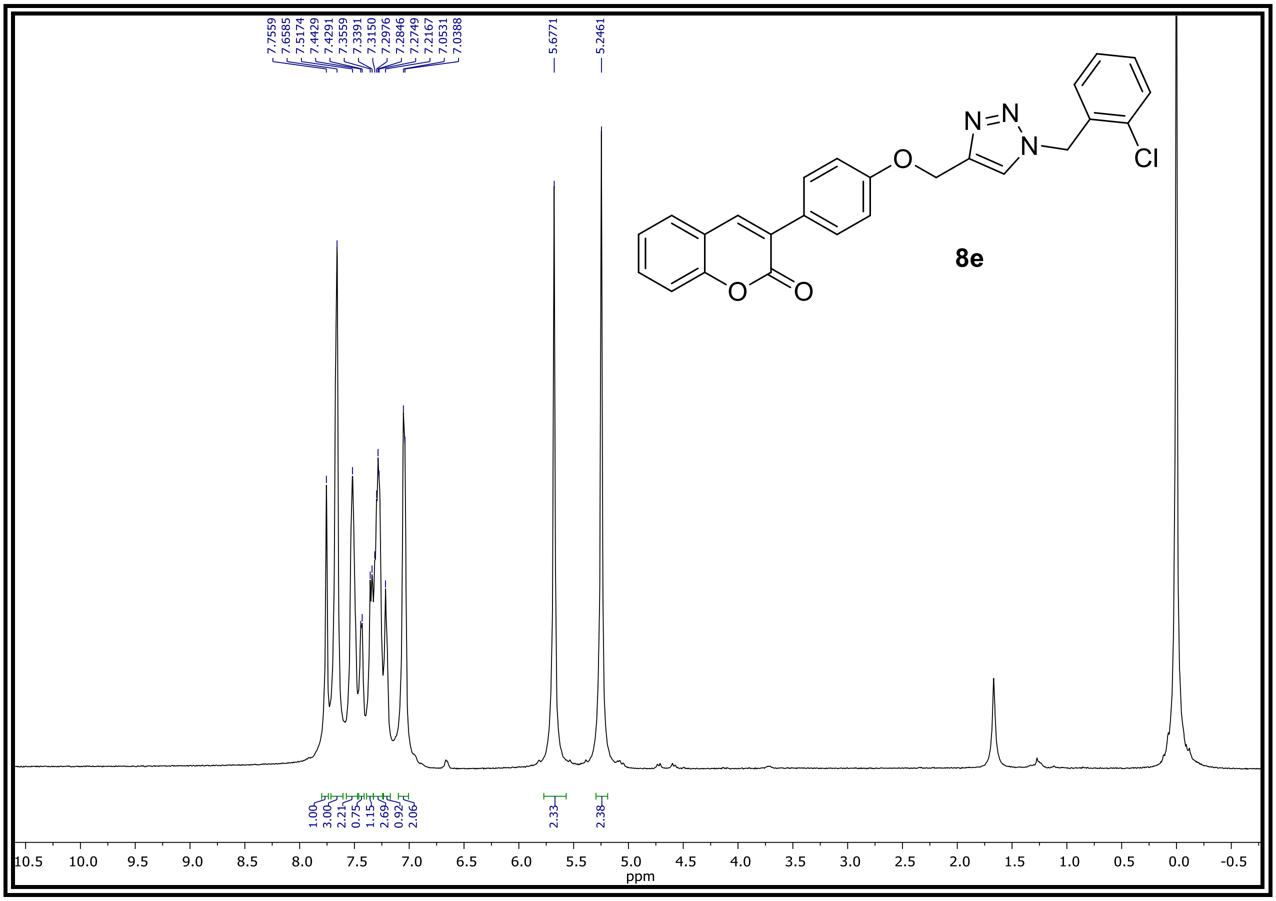

^{1}H NMR (500 MHz, $CDCl_3$) spectrum of 3-(4-((1-(2-Chlorobenzyl)-1*H*-1,2,3-triazol-4-yl)methoxy)phenyl)-2*H*-chromen-2-one

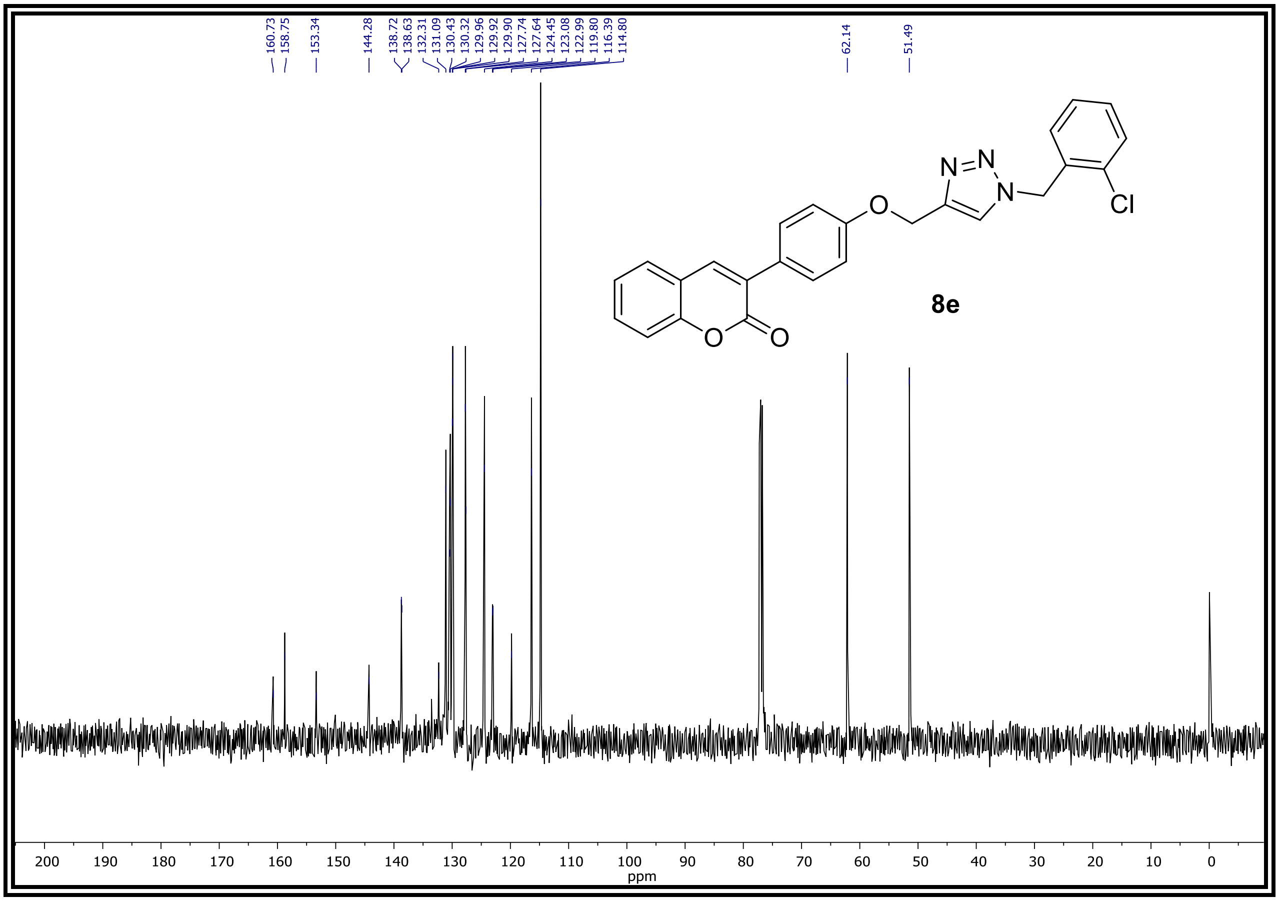

^{13}C NMR (125 MHz, $CDCl_3$) spectrum of 3-(4-((1-(2-Chlorobenzyl)-1*H*-1,2,3-triazol-4-yl)methoxy)phenyl)-2*H*-chromen-2-one

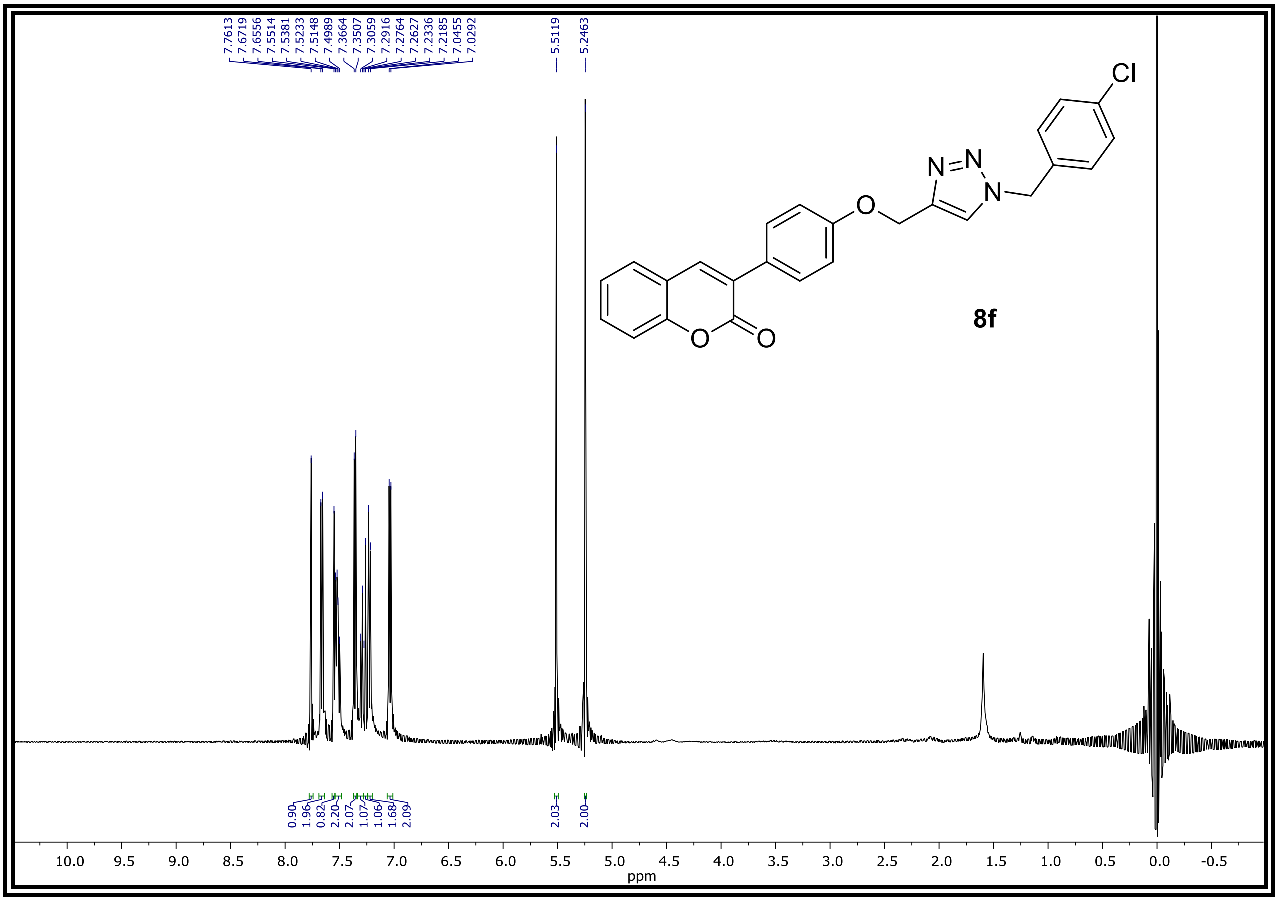

^{1}H NMR (500 MHz, $CDCl_3$) spectrum of 3-(4-((1-(4-Chlorobenzyl)-1*H*-1,2,3-triazol-4-yl)methoxy)phenyl)-2*H*-chromen-2-one

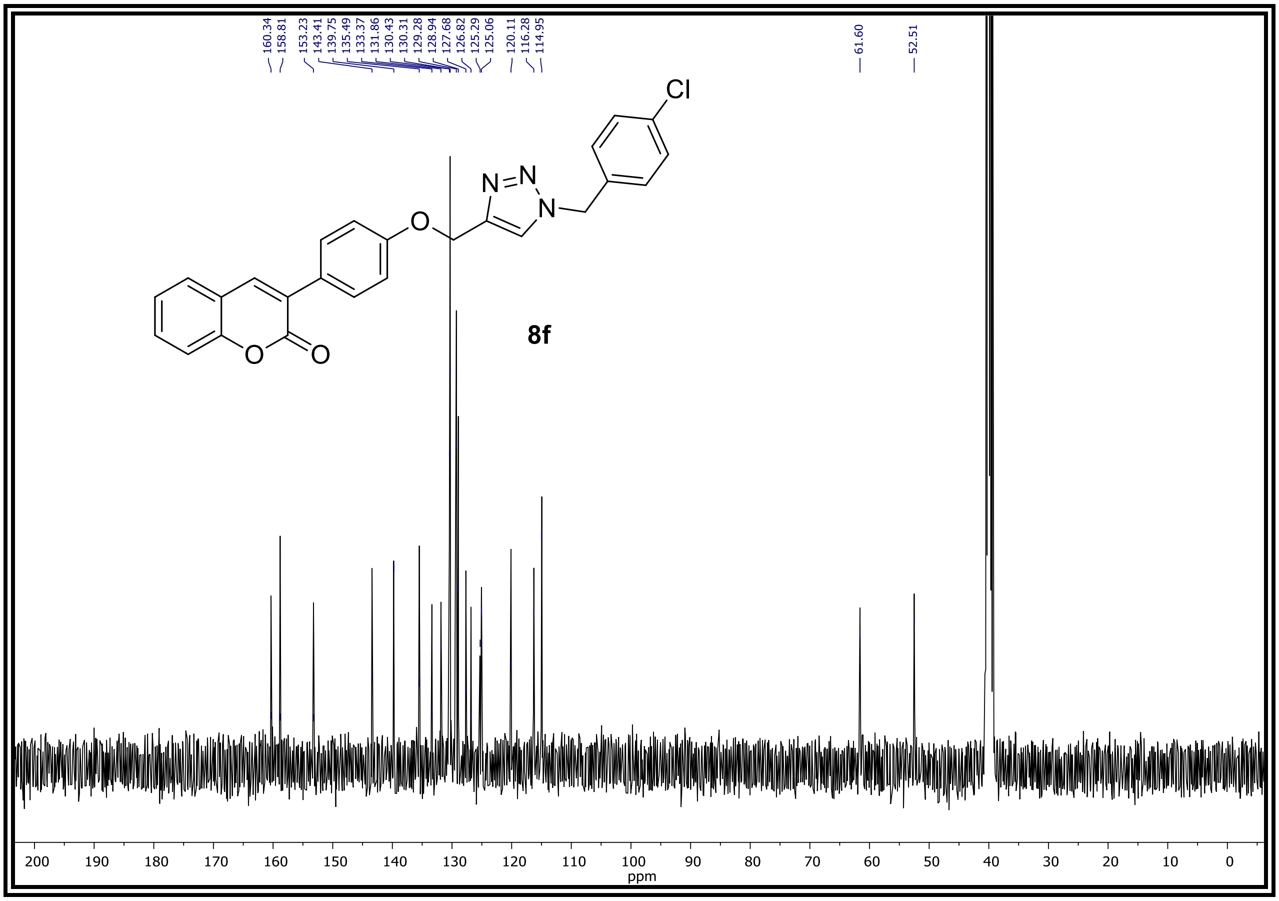

^{13}C NMR (100 MHz, DMSO-d_6) spectrum of 3-(4-((1-(4-Chlorobenzyl)-1*H*-1,2,3-triazol-4-yl)methoxy)phenyl)-2*H*-chromen-2-one

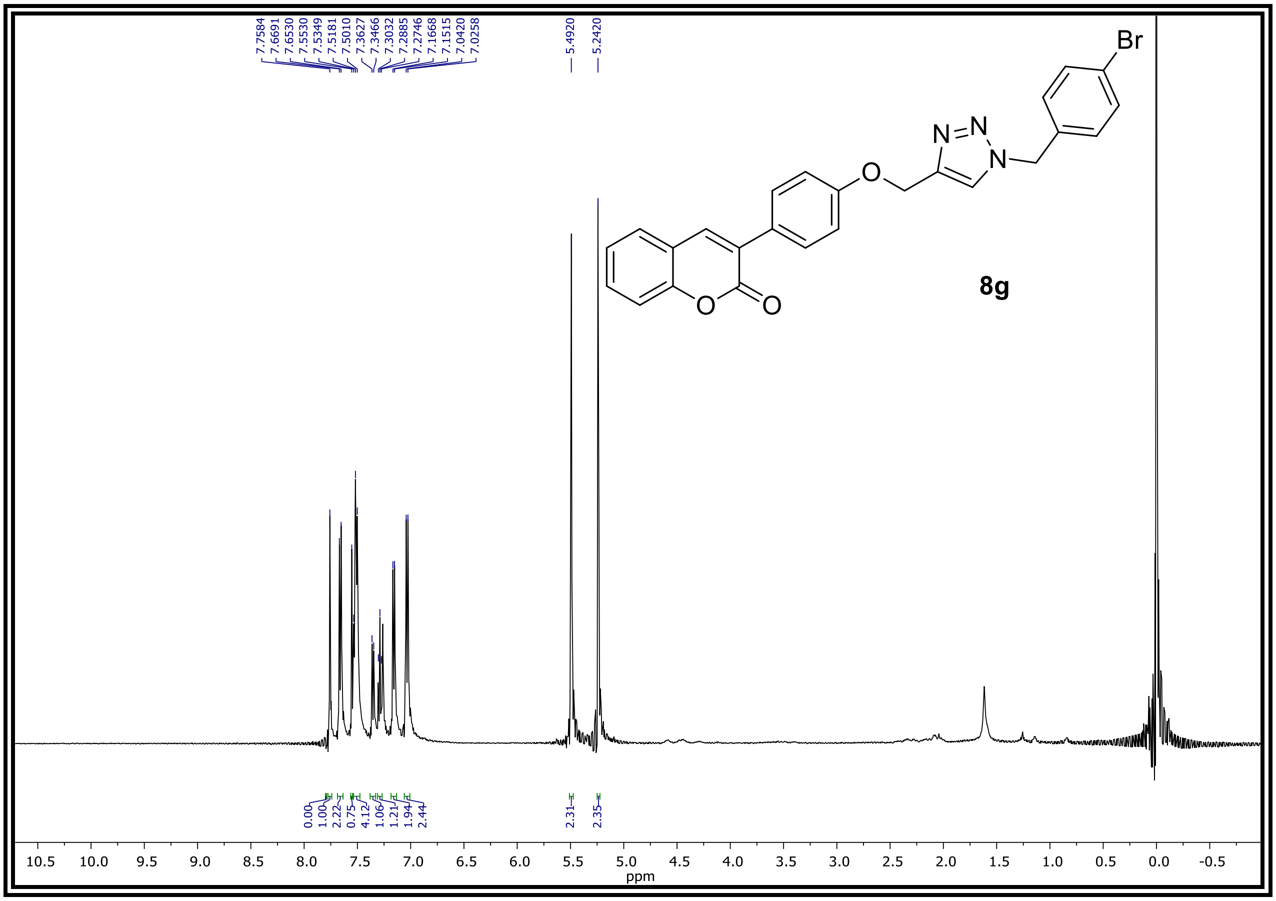

^1H NMR (500 MHz, $CDCl_3$) spectrum of 3-(4-((1-(4-Bromobenzyl)-1*H*-1,2,3-triazol-4-yl)methoxy)phenyl)-2*H*-chromen-2-one

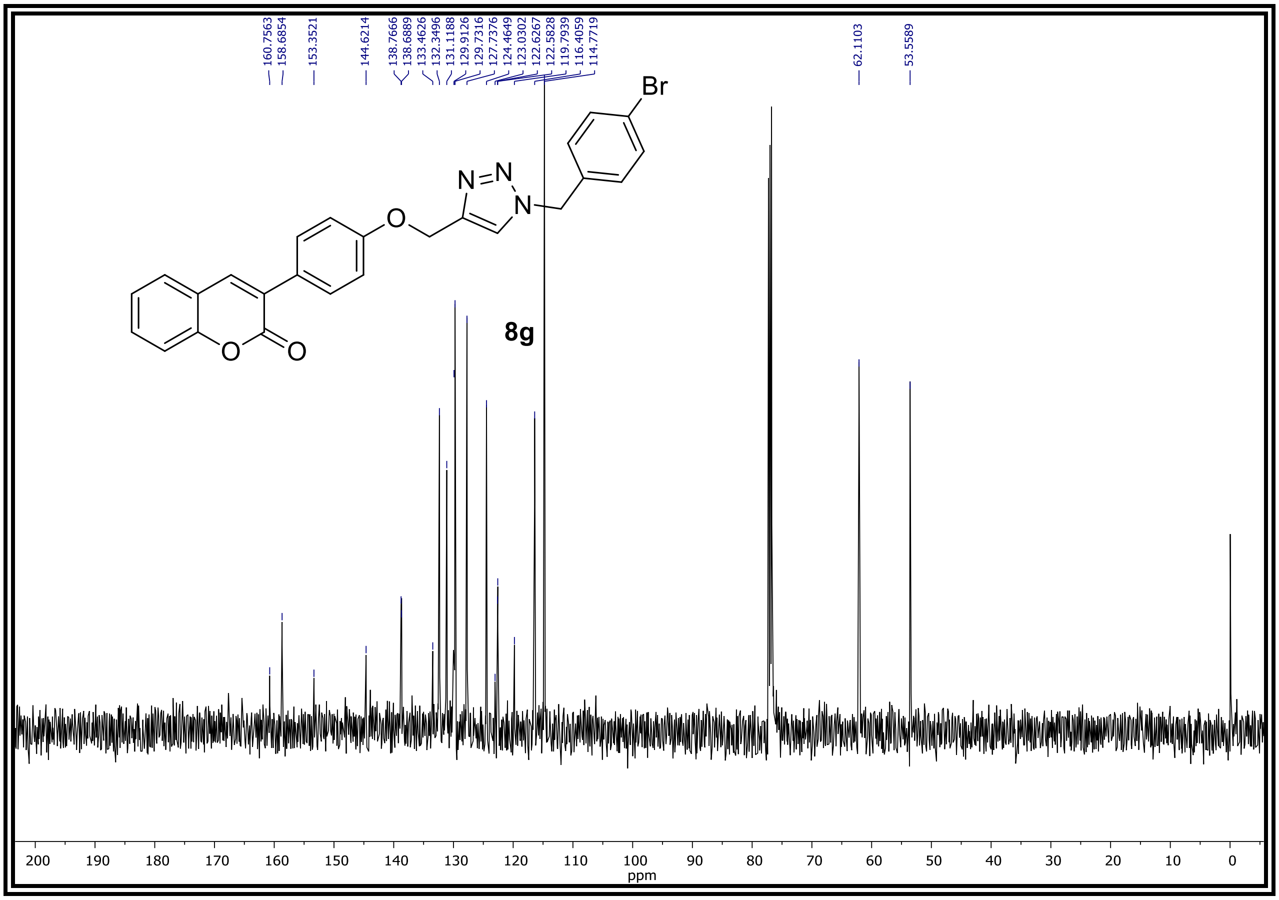

^{13}C NMR (125 MHz, $CDCl_3$) spectrum of 3-(4-((1-(4-Bromobenzyl)-1*H*-1,2,3-triazol-4-yl)methoxy)phenyl)-2*H*-chromen-2-one

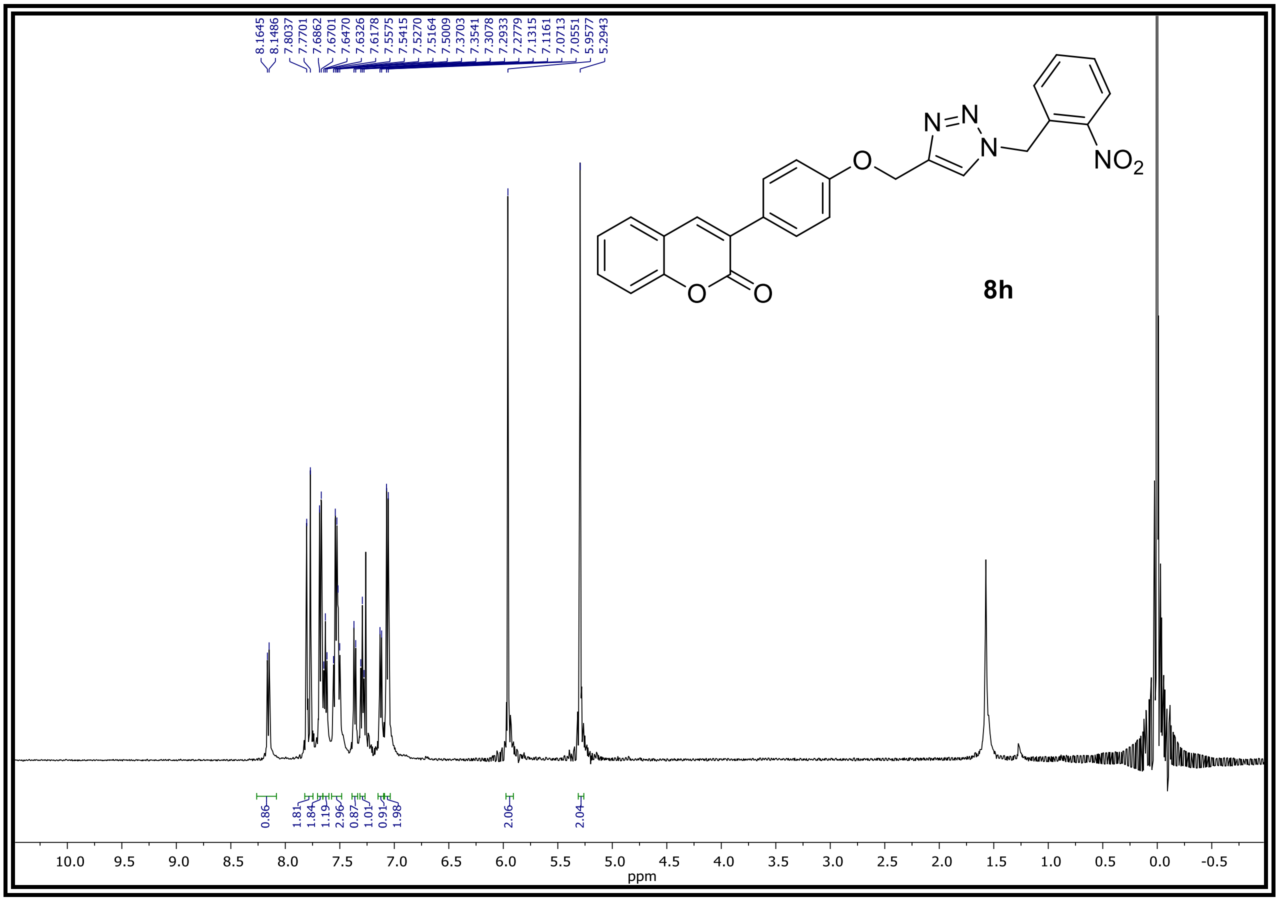

^{1}H NMR (500 MHz, $CDCl_3$) spectrum of 3-(4-((1-(2-Nitrobenzyl)-1*H*-1,2,3-triazol-4-yl)methoxy)phenyl)-2*H*-chromen-2-one

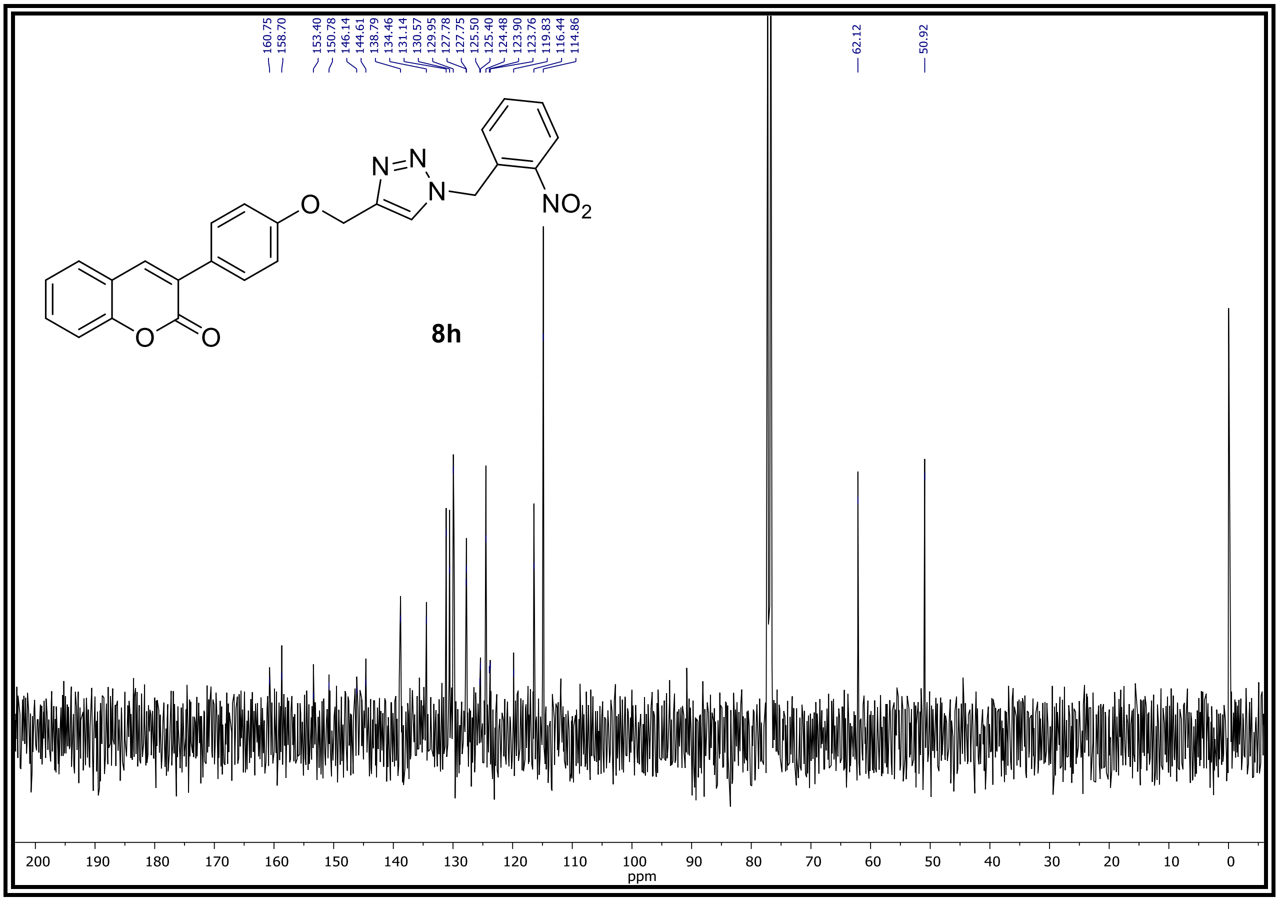

^{13}C NMR (125 MHz, $CDCl_3$) spectrum of 3-(4-((1-(2-Nitrobenzyl)-1*H*-1,2,3-triazol-4-yl)methoxy)phenyl)-2*H*-chromen-2-one

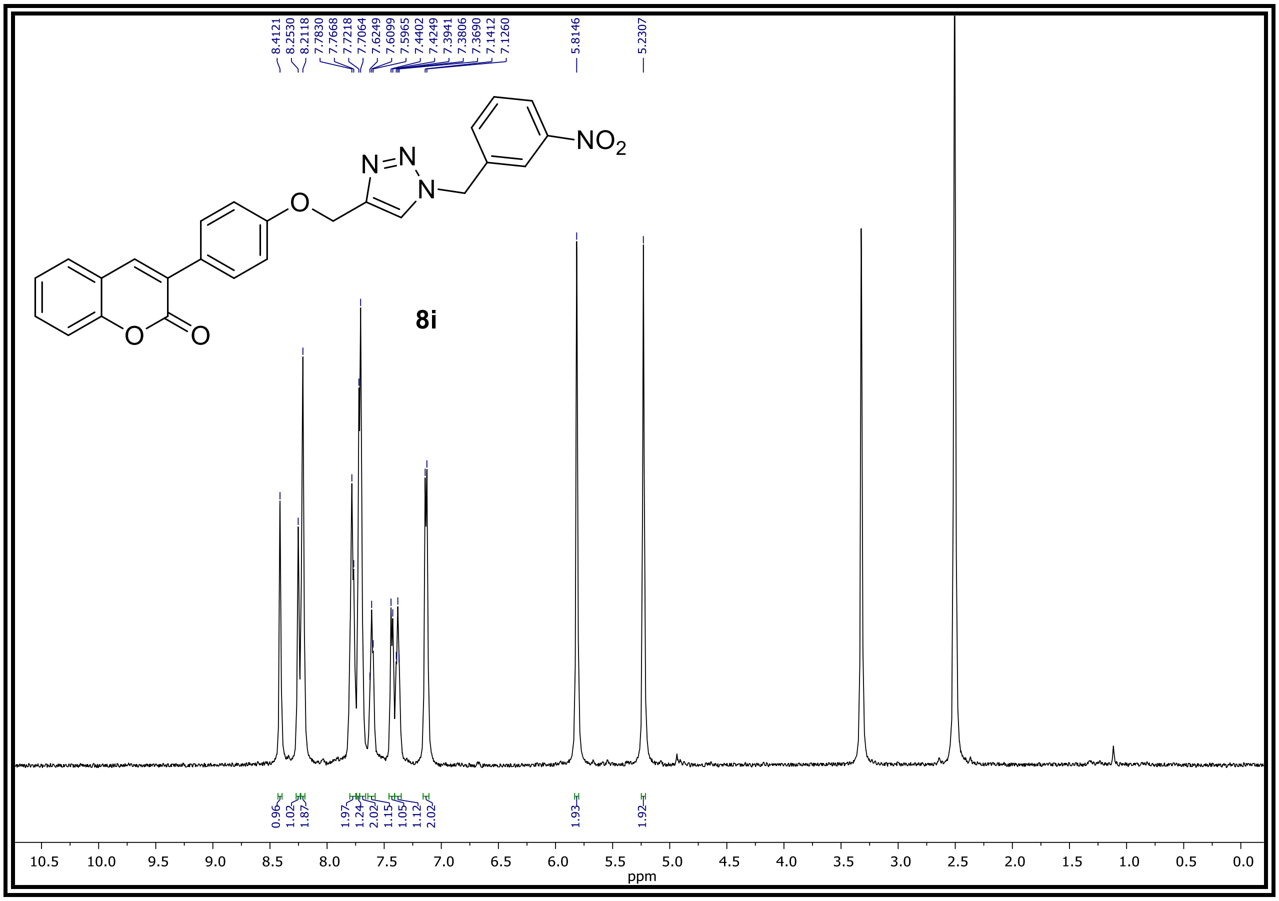

^{1}H NMR (500 MHz, DMSO-d_6) spectrum of 3-(4-((1-(3-Nitrobenzyl)-1*H*-1,2,3-triazol-4-yl)methoxy)phenyl)-2*H*-chromen-2-one

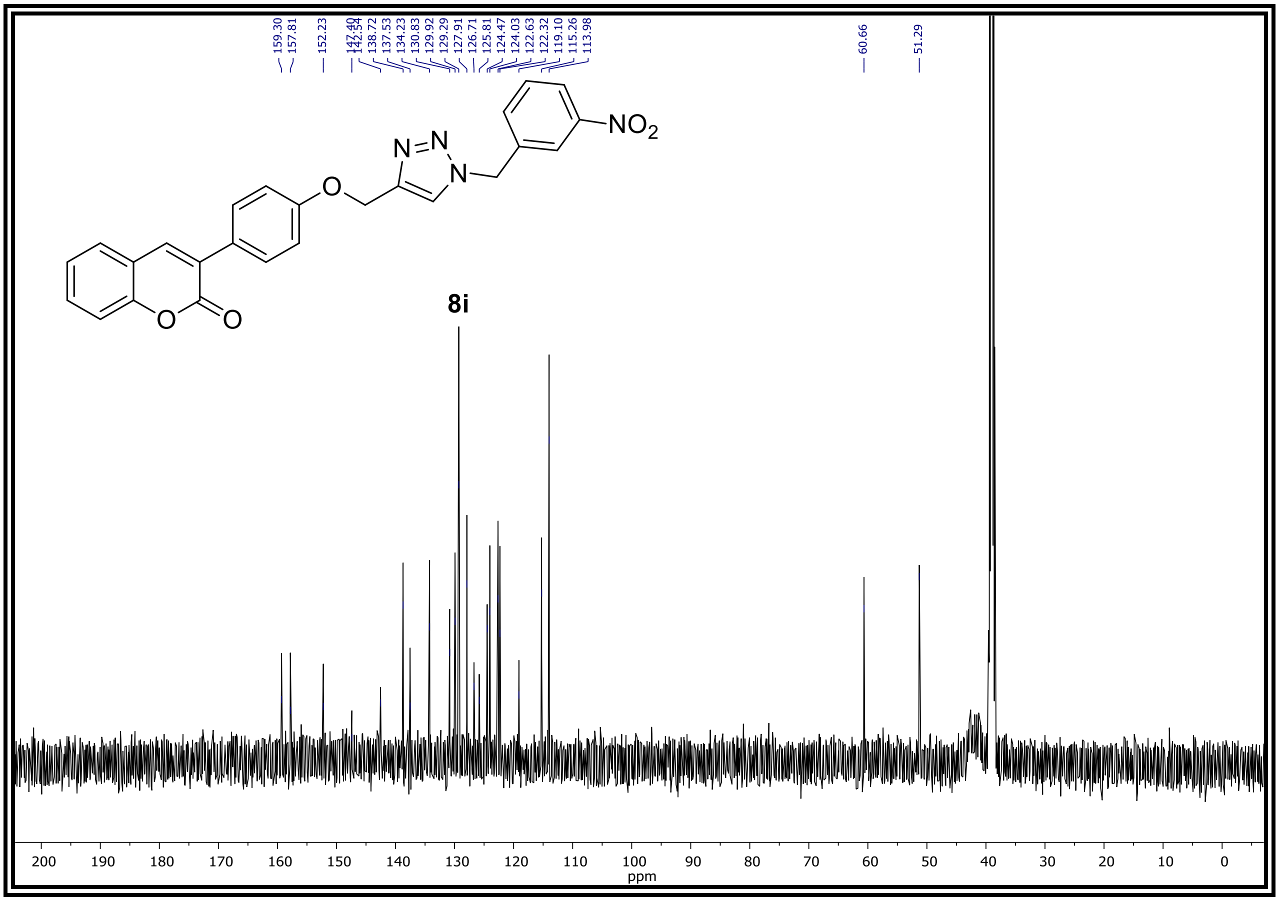

^{13}C NMR (125 MHz, DMSO-d_6) spectrum of 3-(4-((1-(3-Nitrobenzyl)-1*H*-1,2,3-triazol-4-yl)methoxy)phenyl)-2*H*-chromen-2-one

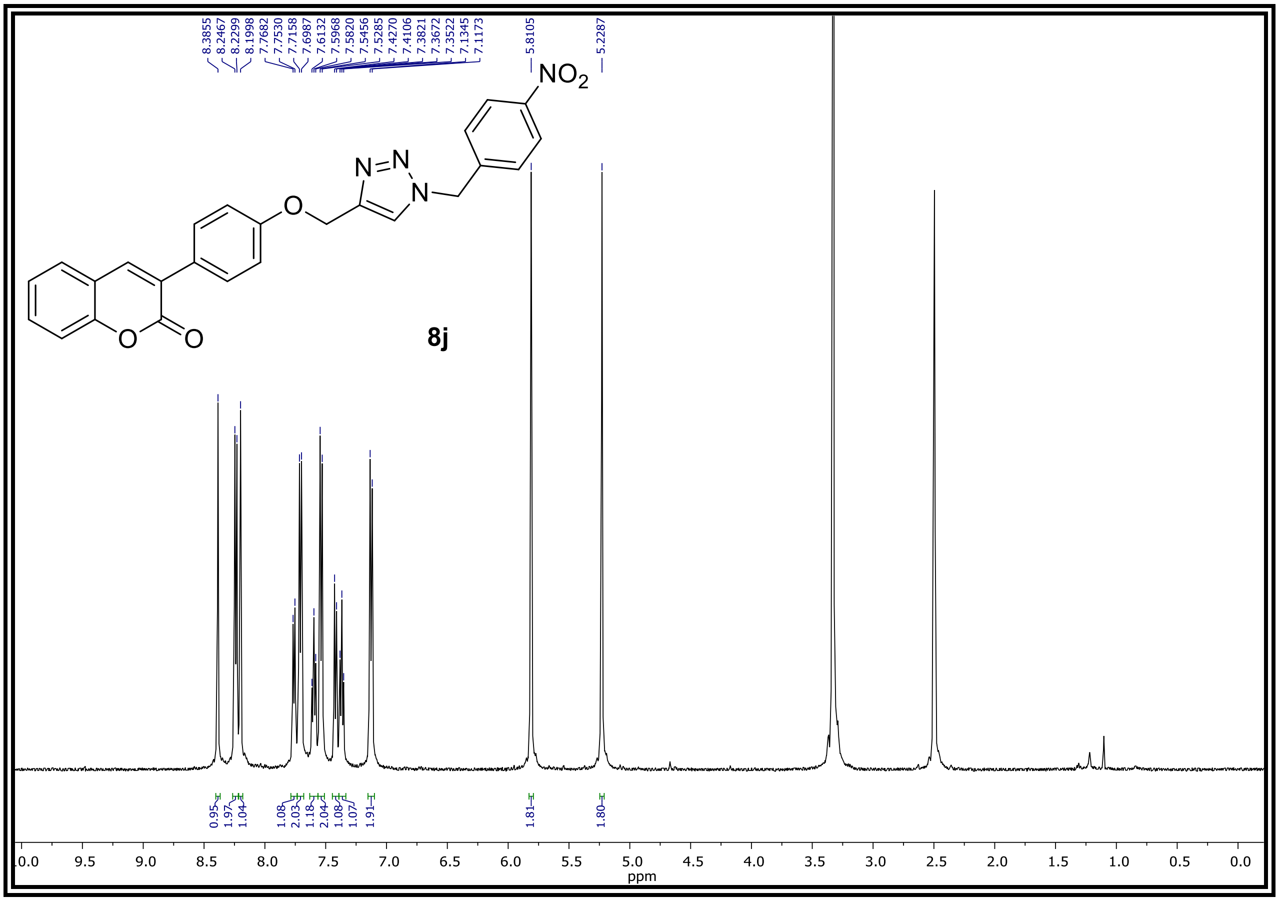

^{1}H NMR (500 MHz, DMSO-d_6) spectrum of 3-(4-((1-(4-Nitrobenzyl)-1*H*-1,2,3-triazol-4-yl)methoxy)phenyl)-2*H*-chromen-2-one

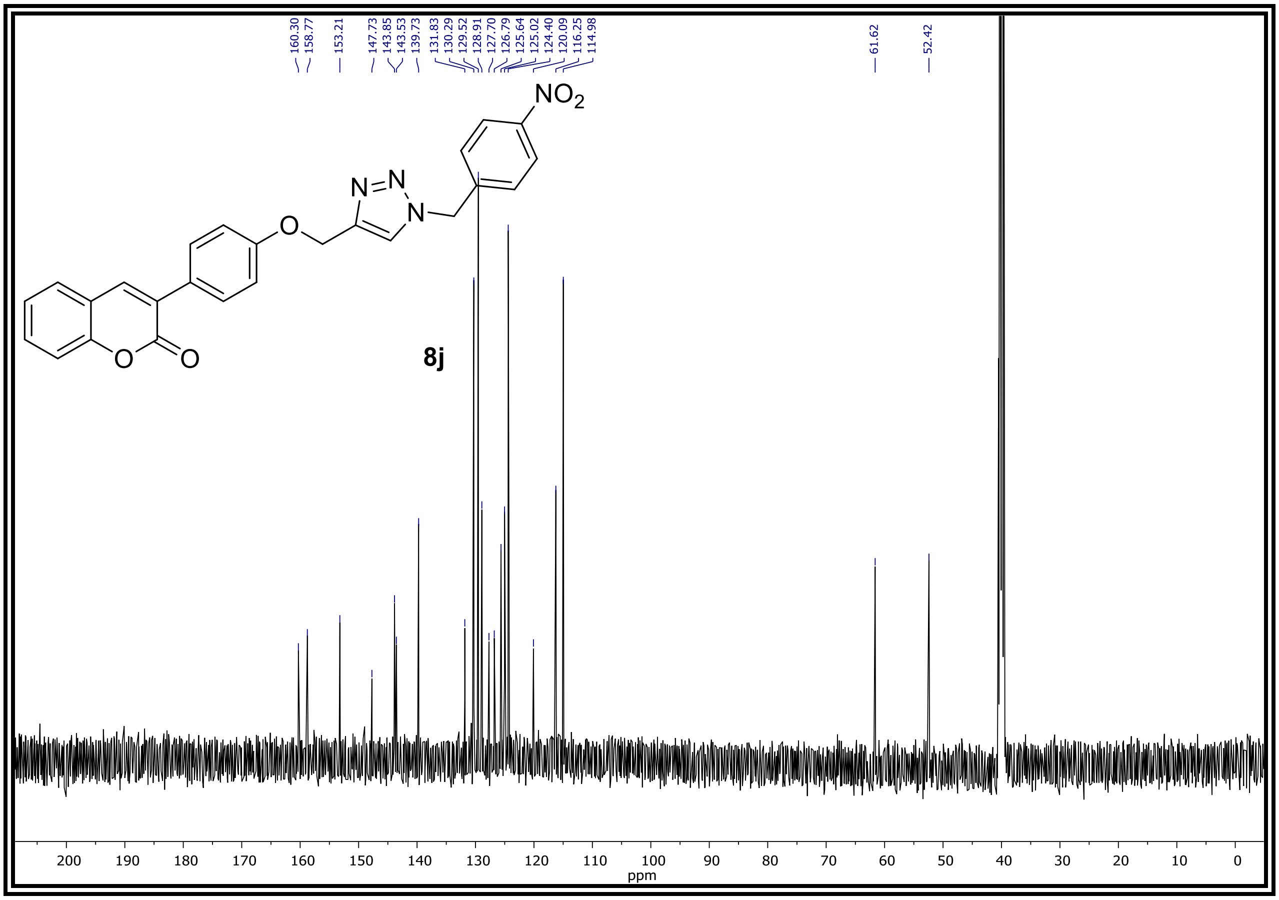

^{13}C NMR (125 MHz, DMSO-d_6) spectrum of 3-(4-((1-(4-Nitrobenzyl)-1*H*-1,2,3-triazol-4-yl)methoxy)phenyl)-2*H*-chromen-2-one

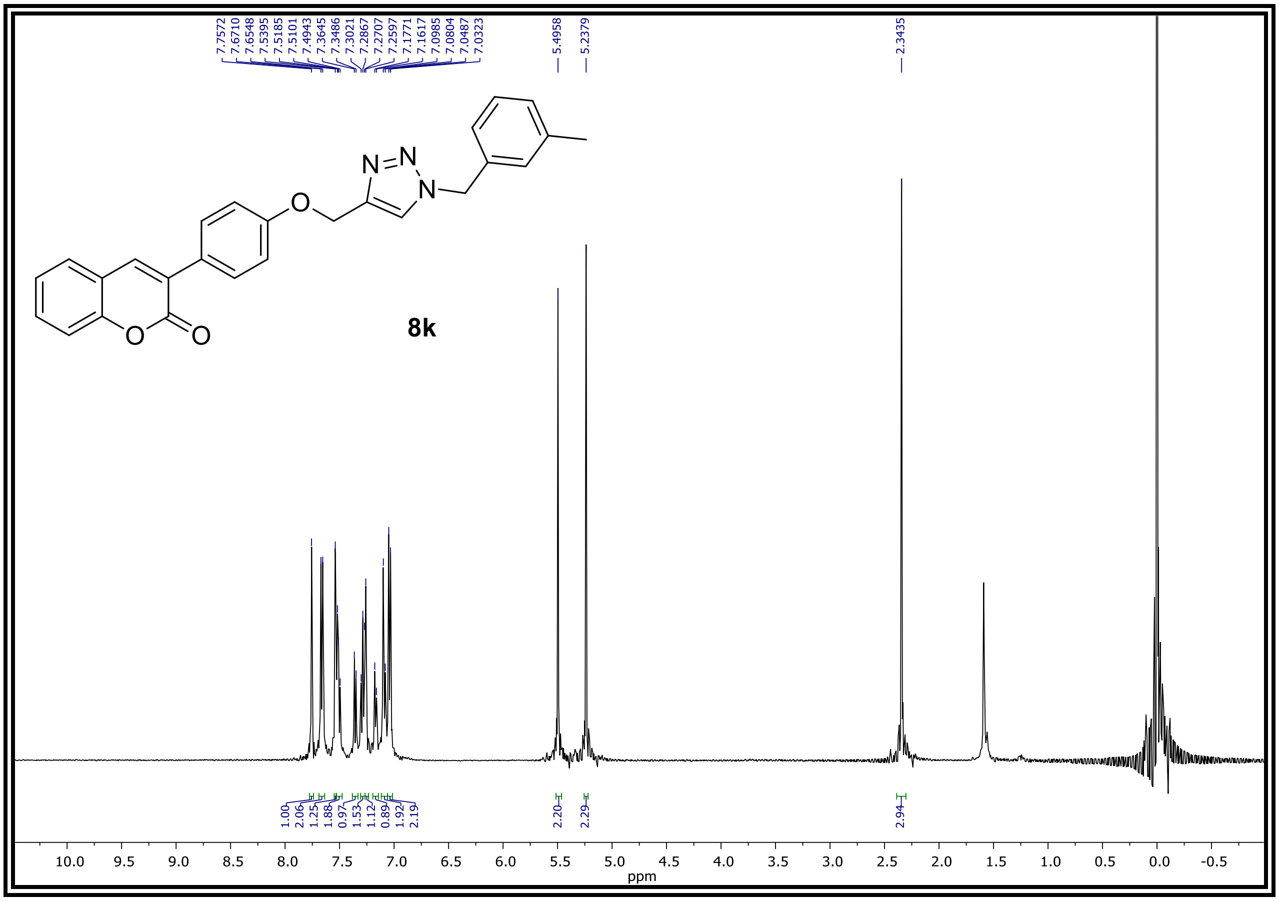

^{1}H NMR (500 MHz, $CDCl_3$) spectrum of 3-(4-((1-(3-Methylbenzyl)-1*H*-1,2,3-triazol-4-yl)methoxy)phenyl)-2*H*-chromen-2-one

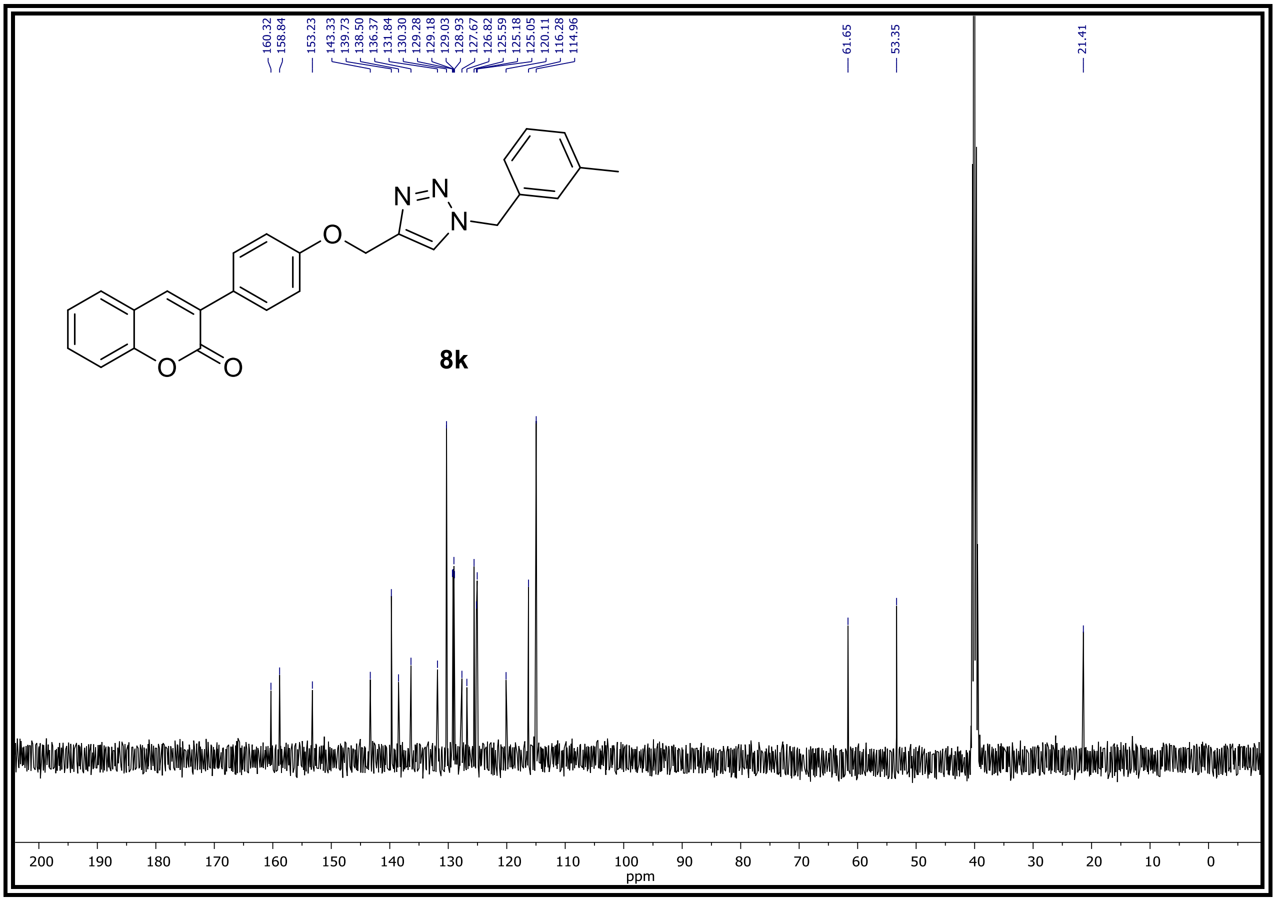

^{13}C NMR (125 MHz, DMSO-d_6) spectrum of 3-(4-((1-(3-Methylbenzyl)-1*H*-1,2,3-triazol-4-yl)methoxy)phenyl)-2*H*-chromen-2-one

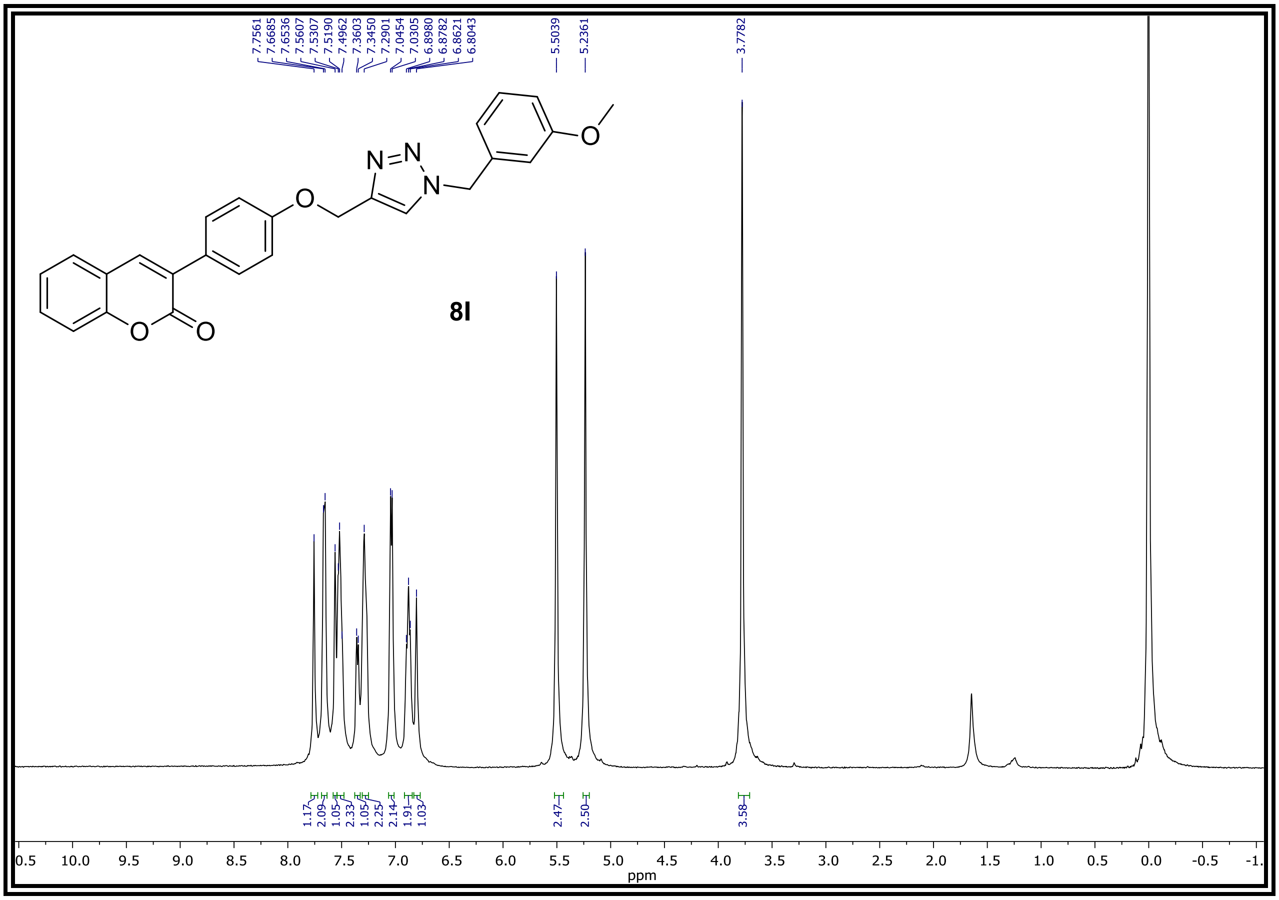

^{1}H NMR (500 MHz, $CDCl_3$) spectrum of 3-(4-((1-(3-Methoxybenzyl)-1*H*-1,2,3-triazol-4-yl)methoxy)phenyl)-2*H*-chromen-2-one

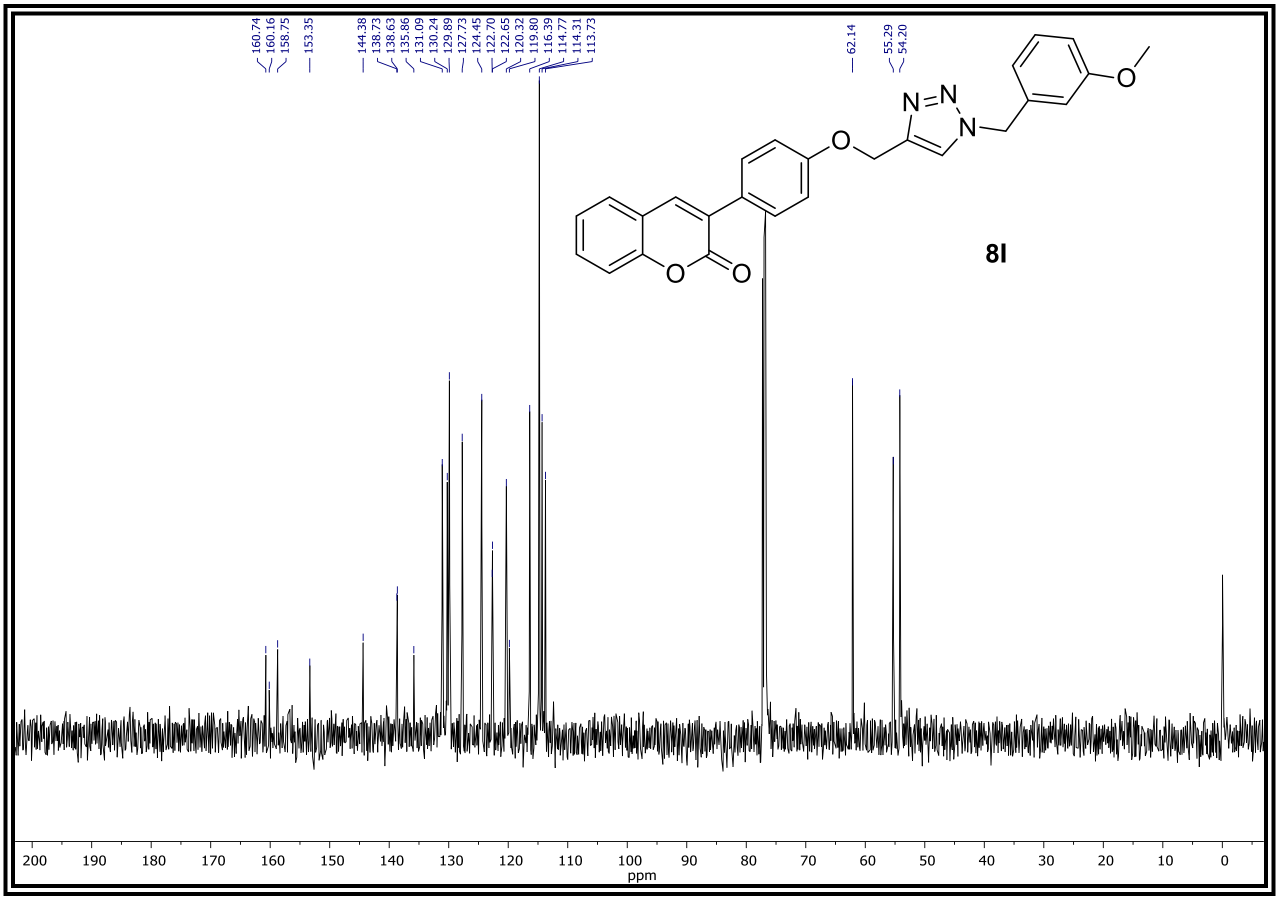

^{13}C NMR (125 MHz, $CDCl_3$) spectrum of 3-(4-((1-(3-Methoxybenzyl)-1*H*-1,2,3-triazol-4-yl)methoxy)phenyl)-2*H*-chromen-2-one

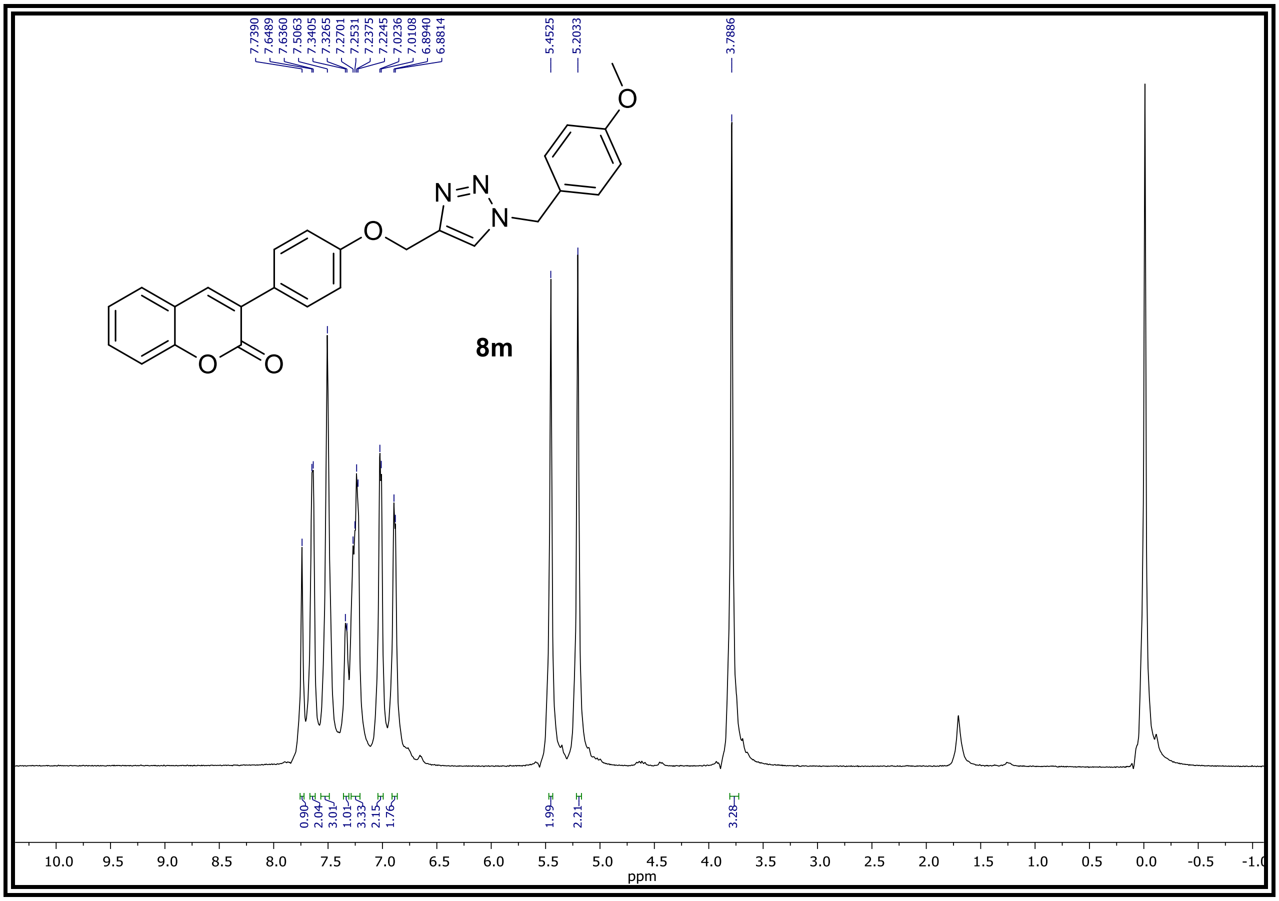

^{1}H NMR (500 MHz, $CDCl_3$) spectrum of 3-(4-((1-(4-Methoxybenzyl)-1*H*-1,2,3-triazol-4-yl)methoxy)phenyl)-2*H*-chromen-2-one

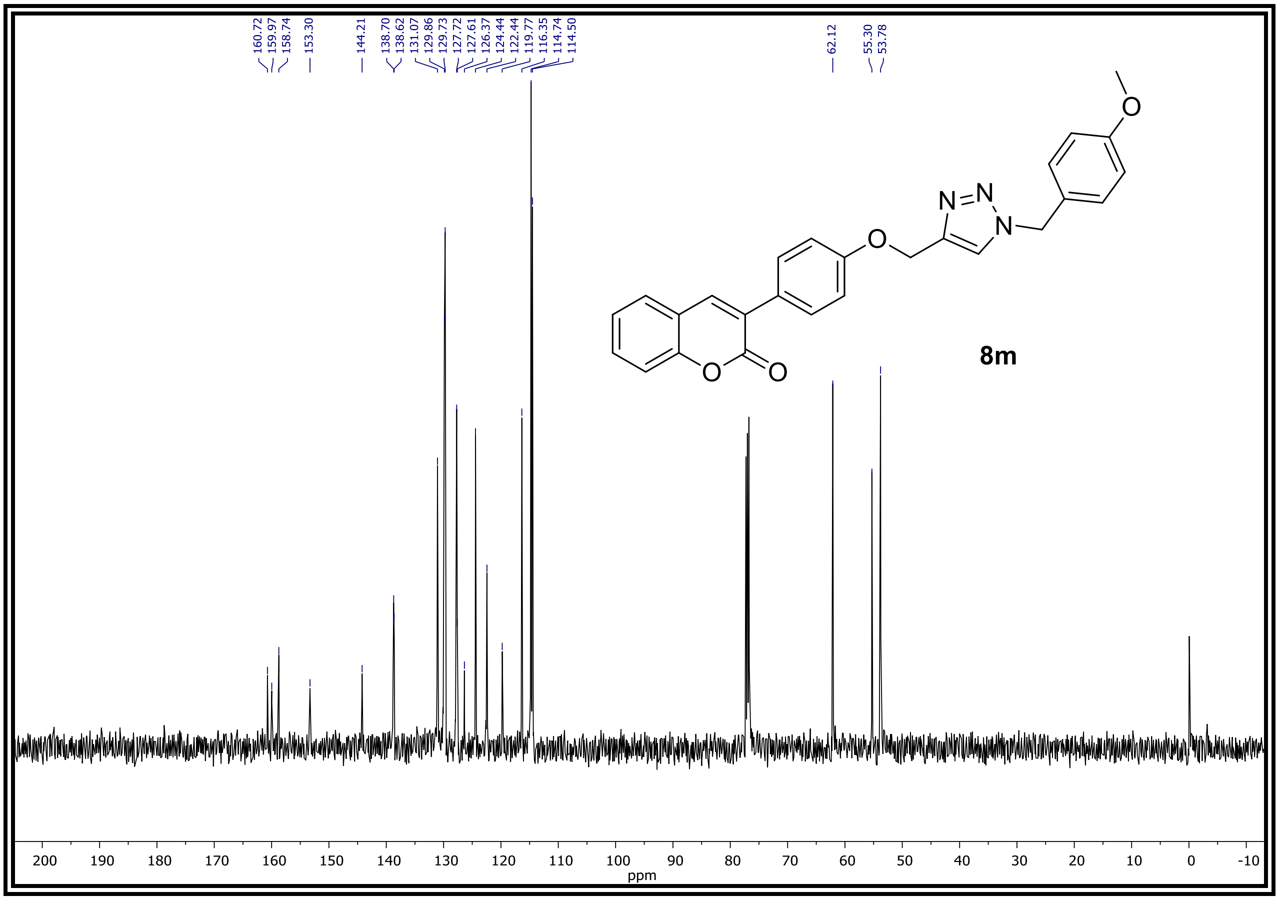

^{13}C NMR (125 MHz, $CDCl_3$) spectrum of 3-(4-((1-(4-Methoxybenzyl)-1*H*-1,2,3-triazol-4-yl)methoxy)phenyl)-2*H*-chromen-2-one

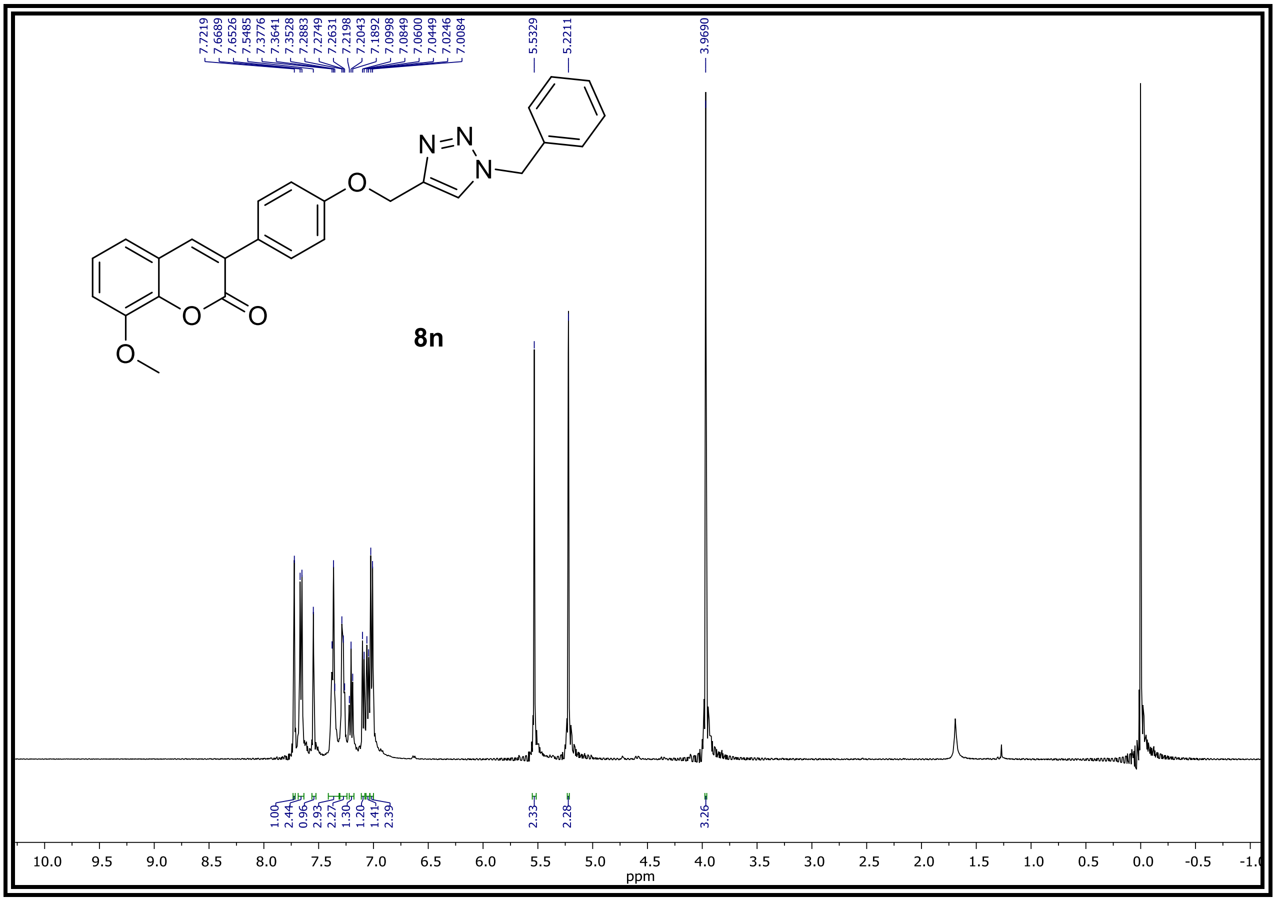

^{1}H NMR (500 MHz, $CDCl_3$) spectrum of 3-(4-((1-Benzyl-1*H*-1,2,3-triazol-4-yl)methoxy)phenyl)-8-methoxy-2*H*-chromen-2-one

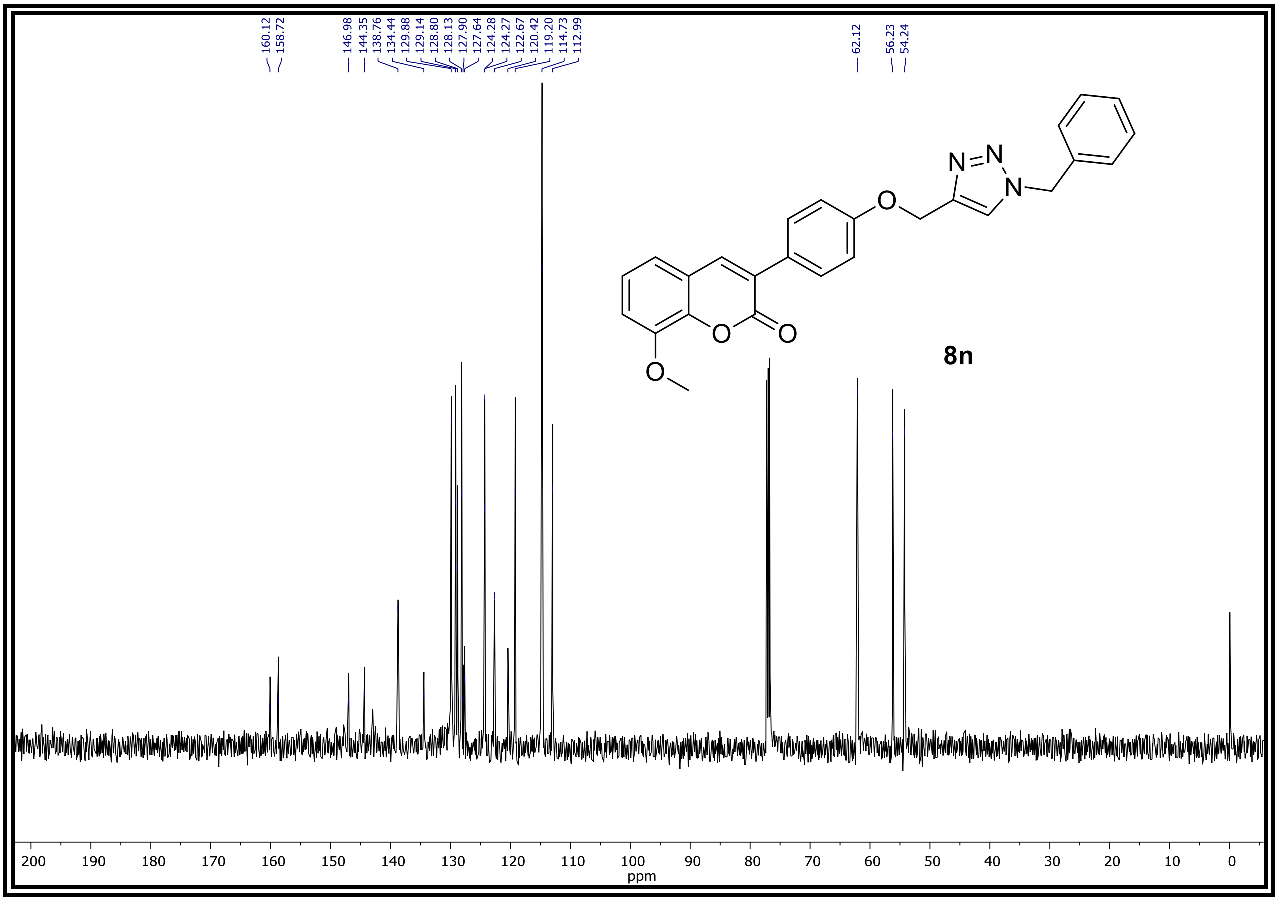

^{13}C NMR (125 MHz, $CDCl_3$) spectrum of 3-(4-((1-Benzyl-1*H*-1,2,3-triazol-4-yl)methoxy)phenyl)-8-methoxy-2*H*-chromen-2-one

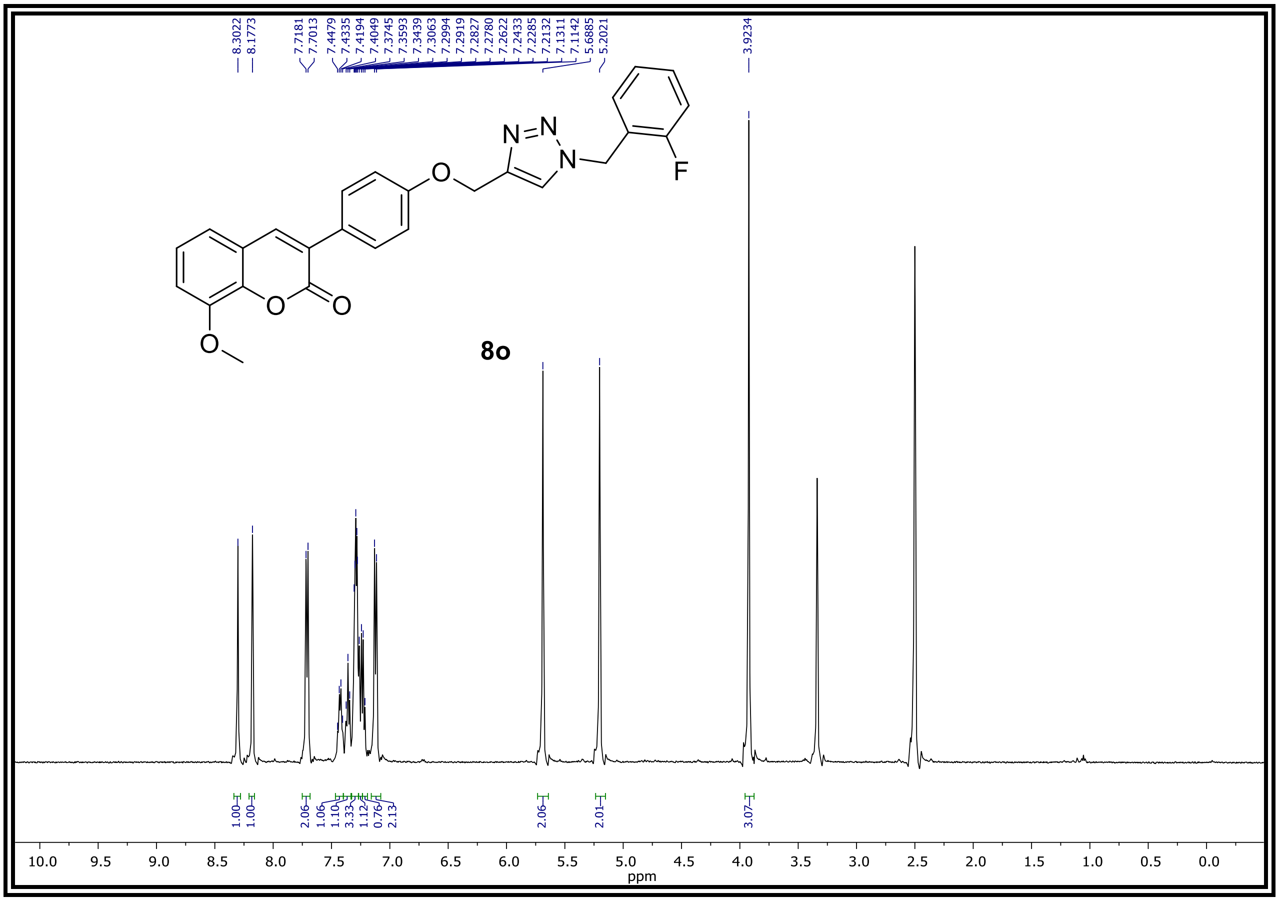

^{1}H NMR (500 MHz, DMSO-d_6) spectrum of 3-(4-((1-(2-Fluorobenzyl)-1*H*-1,2,3-triazol-4-yl)methoxy)phenyl)-8-methoxy-2*H*-chromen-2-one

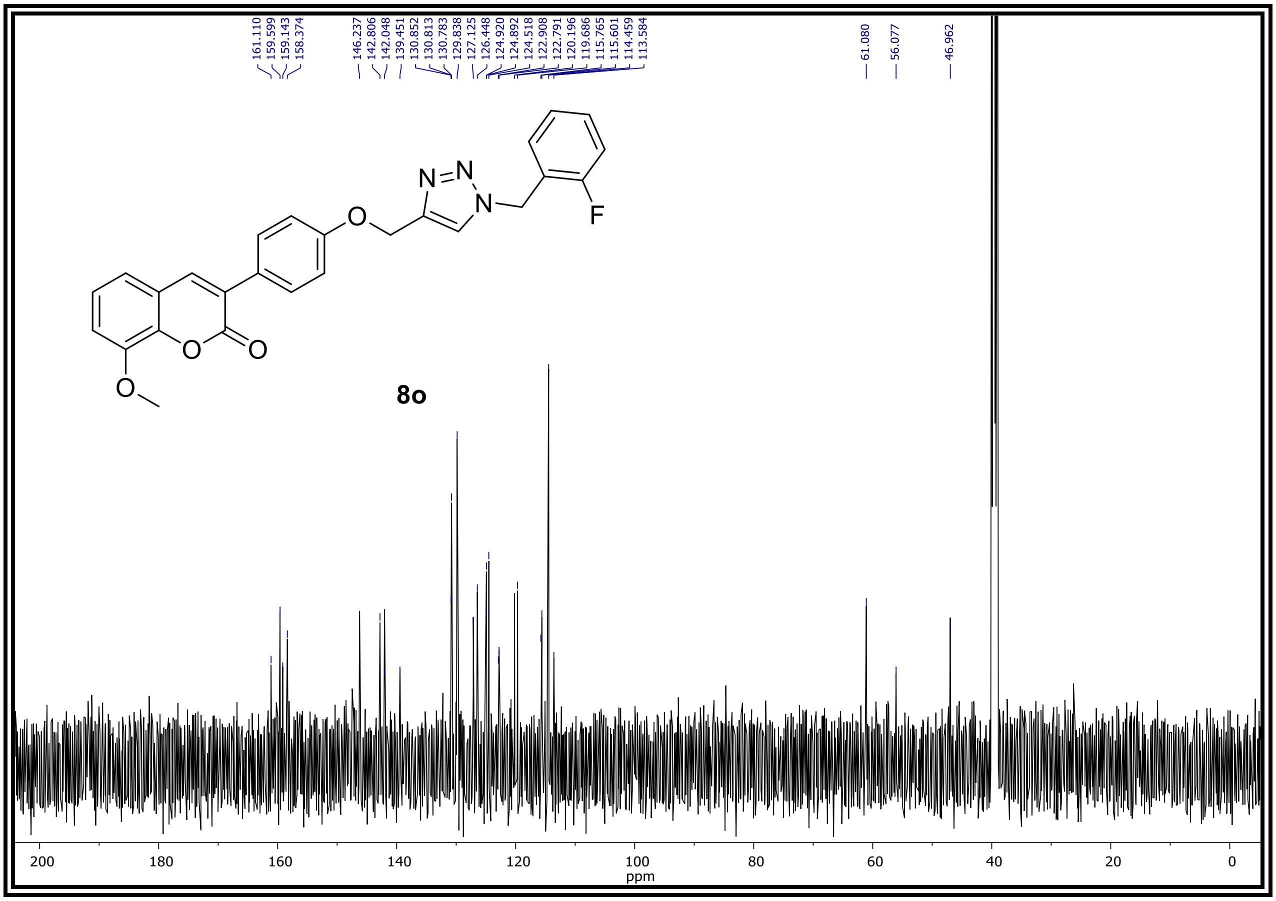

^{13}C NMR (125 MHz, DMSO-d_6) spectrum of 3-(4-((1-(2-Fluorobenzyl)-1*H*-1,2,3-triazol-4-yl)methoxy)phenyl)-8-methoxy-2*H*-chromen-2-one

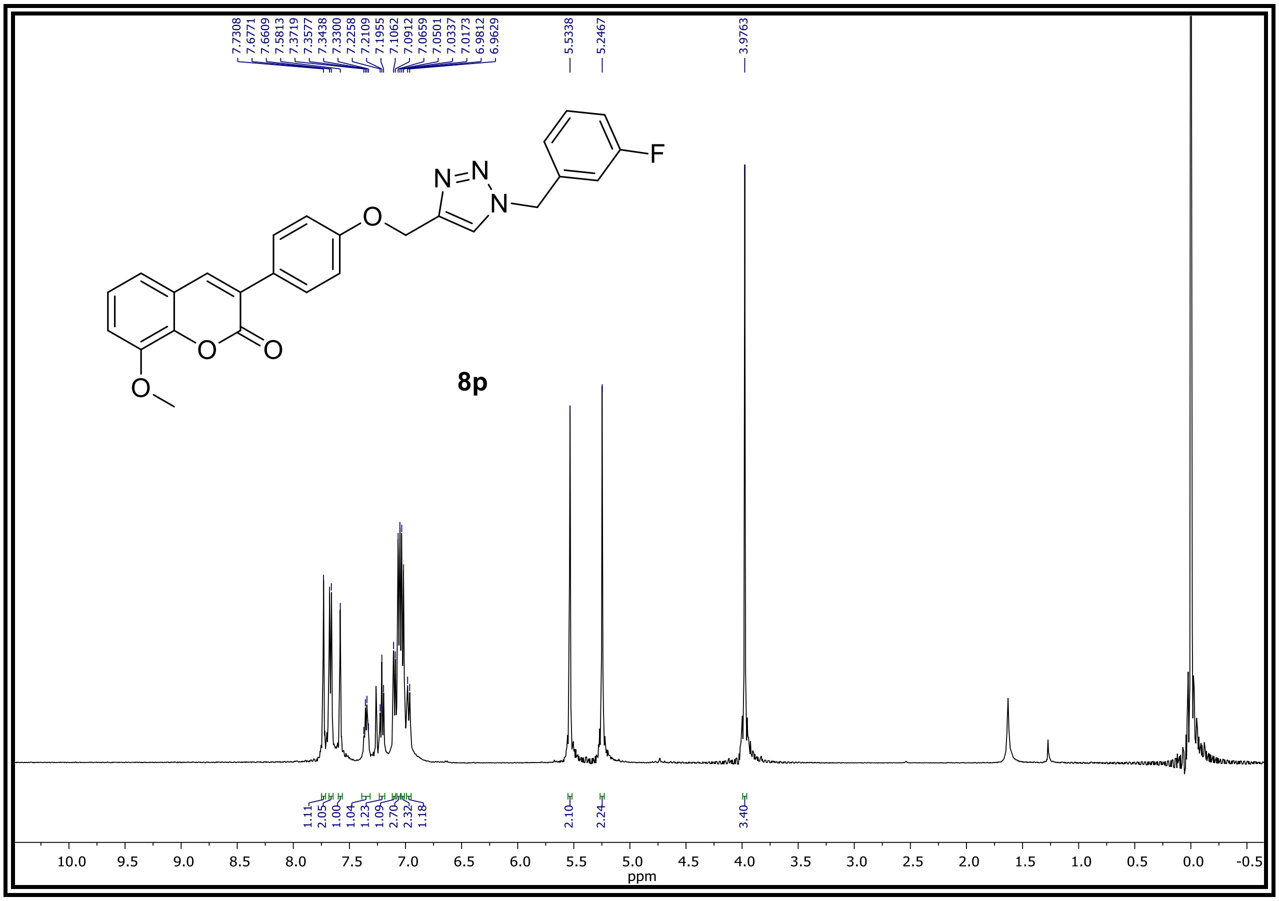

^{1}H NMR (500 MHz, $CDCl_3$) spectrum of 3-(4-((1-(3-Fluorobenzyl)-1*H*-1,2,3-triazol-4-yl)methoxy)phenyl)-8-methoxy-2*H*-chromen-2-one

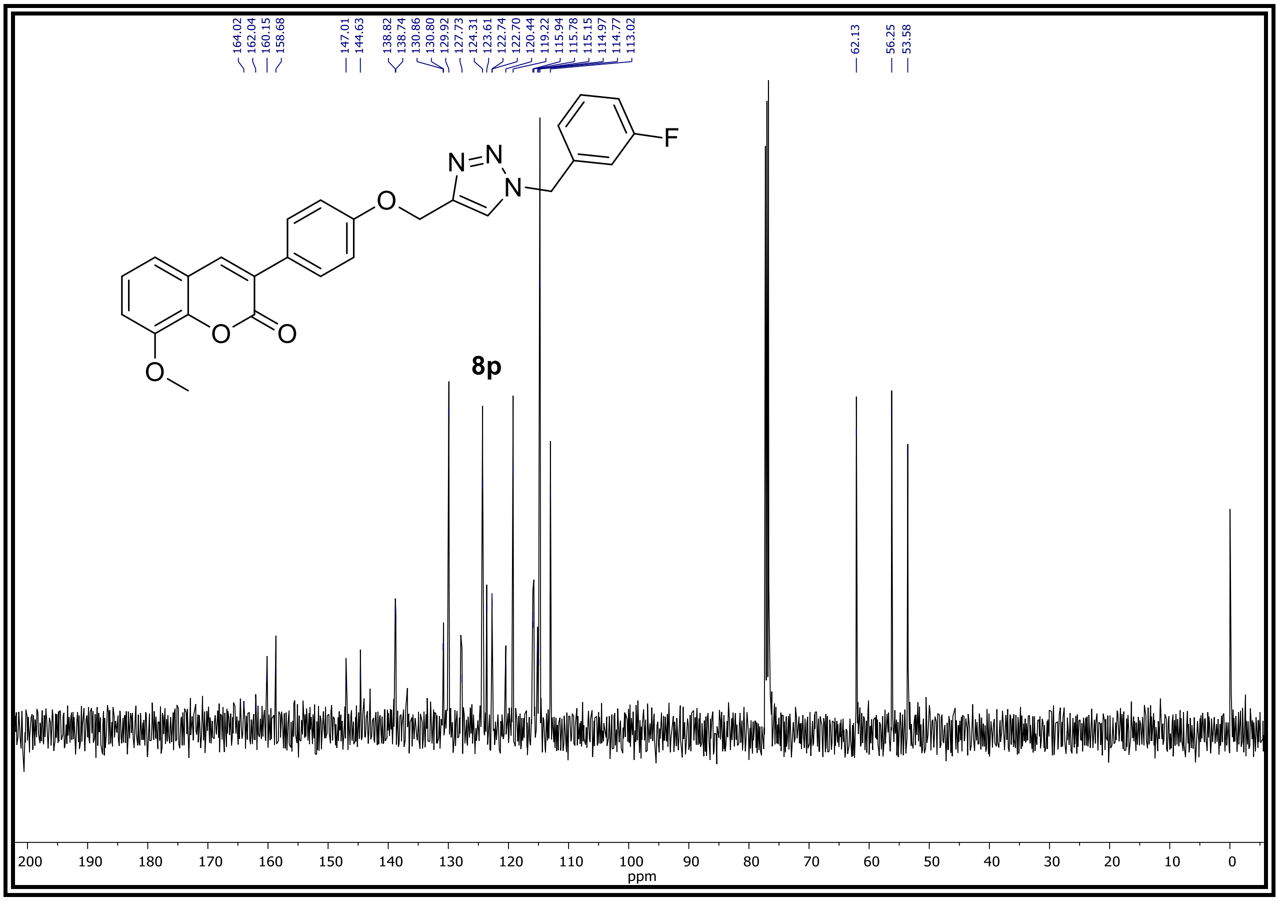

^{13}C NMR (125 MHz, $CDCl_3$) spectrum of 3-(4-((1-(3-Fluorobenzyl)-1*H*-1,2,3-triazol-4-yl)methoxy)phenyl)-8-methoxy-2*H*-chromen-2-one

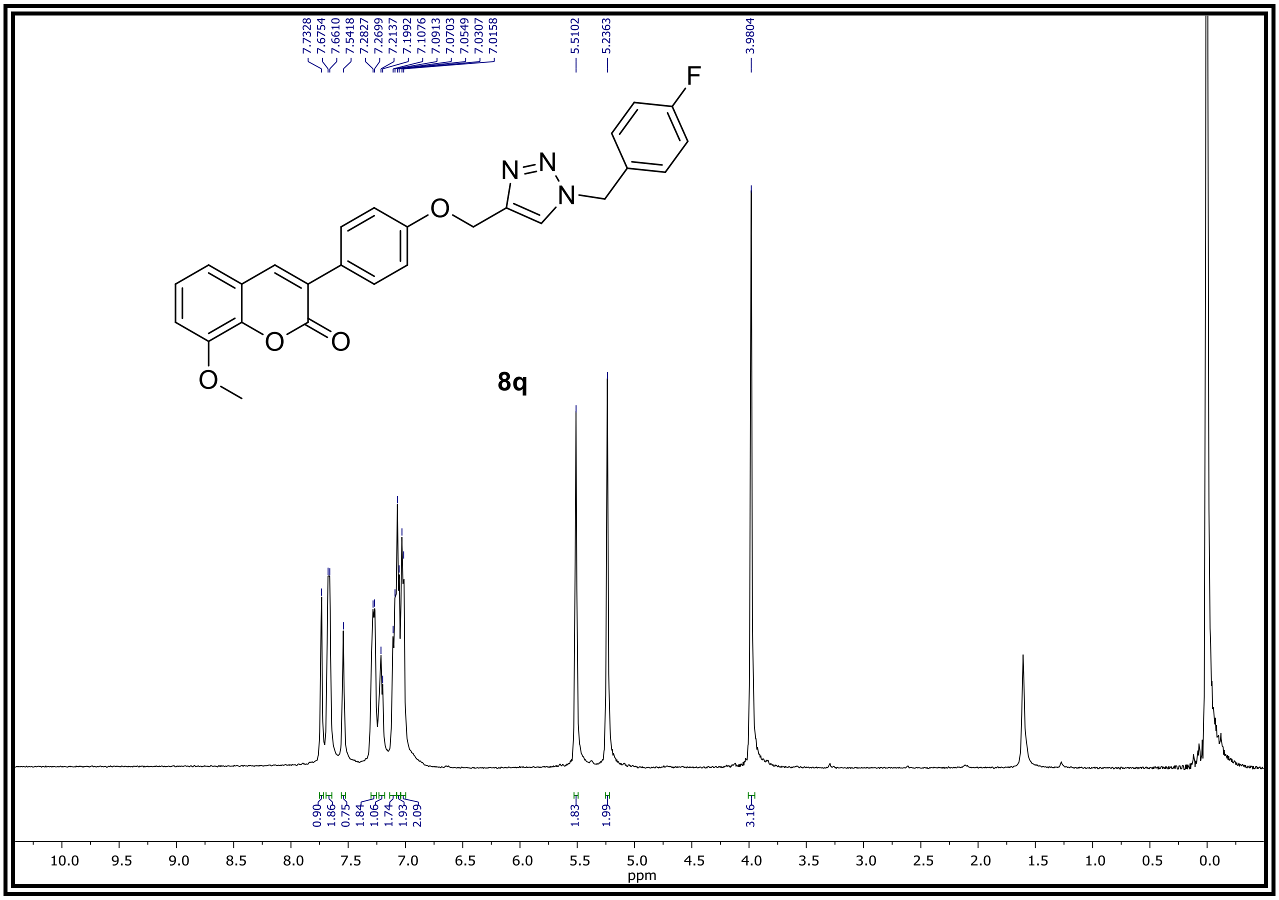

^{1}H NMR (500 MHz, $CDCl_3$) spectrum of 3-(4-((1-(4-Fluorobenzyl)-1*H*-1,2,3-triazol-4-yl)methoxy)phenyl)-8-methoxy-2*H*-chromen-2-one

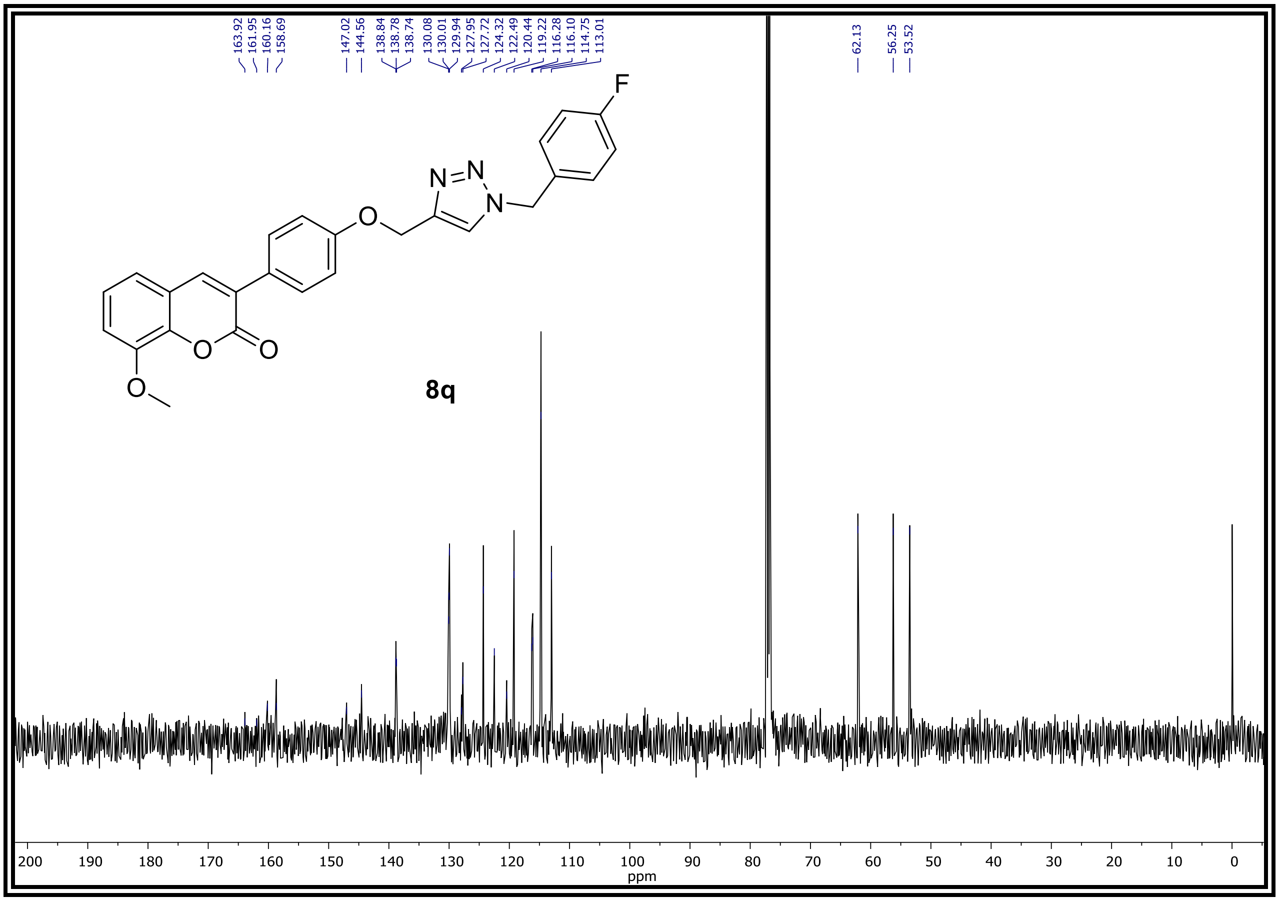

^{13}C NMR (125 MHz, $CDCl_3$) spectrum of 3-(4-((1-(4-Fluorobenzyl)-1*H*-1,2,3-triazol-4-yl)methoxy)phenyl)-8-methoxy-2*H*-chromen-2-one

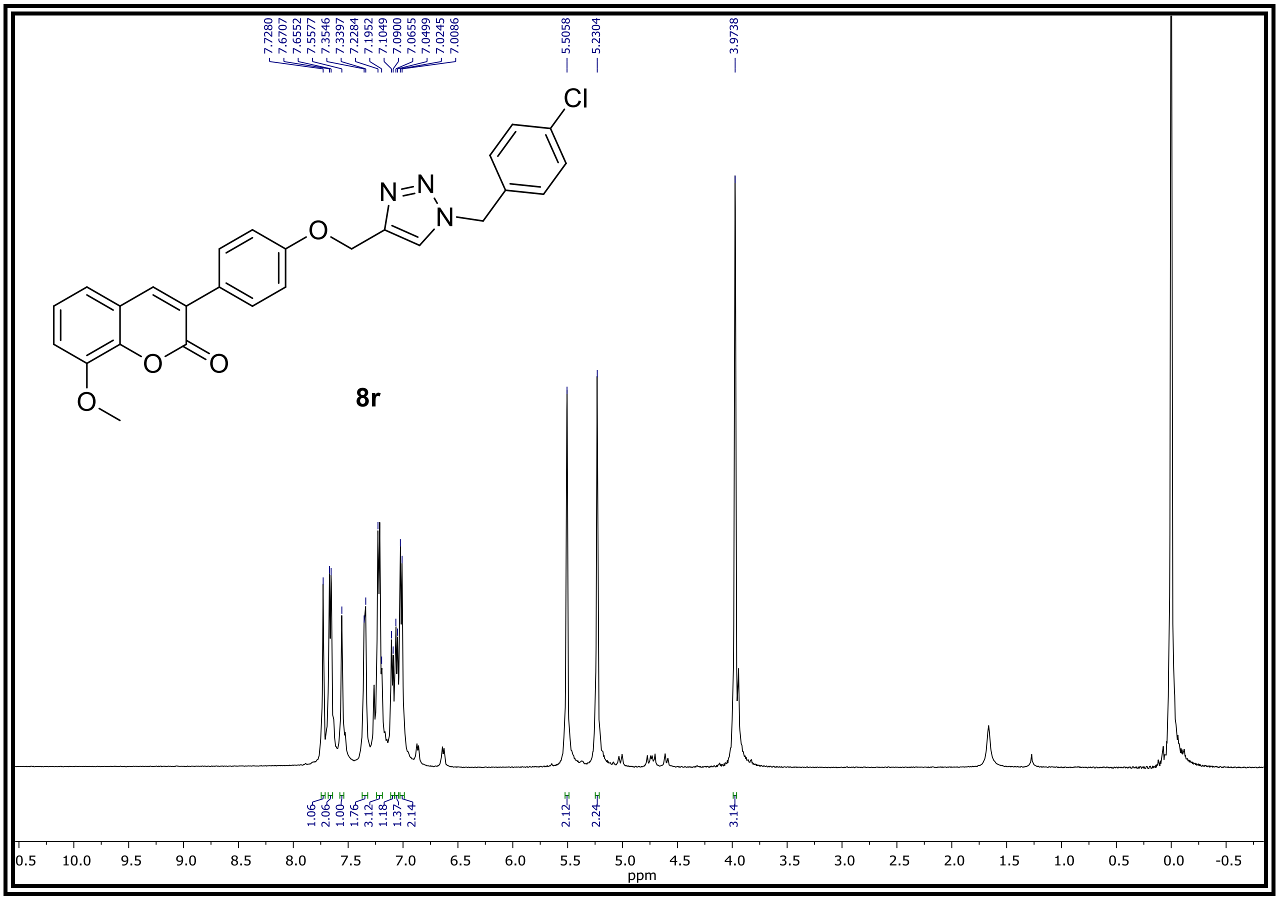

^{1}H NMR (500 MHz, $CDCl_3$) spectrum of 3-(4-((1-(4-Chlorobenzyl)-1*H*-1,2,3-triazol-4-yl)methoxy)phenyl)-8-methoxy-2*H*-chromen-2-one

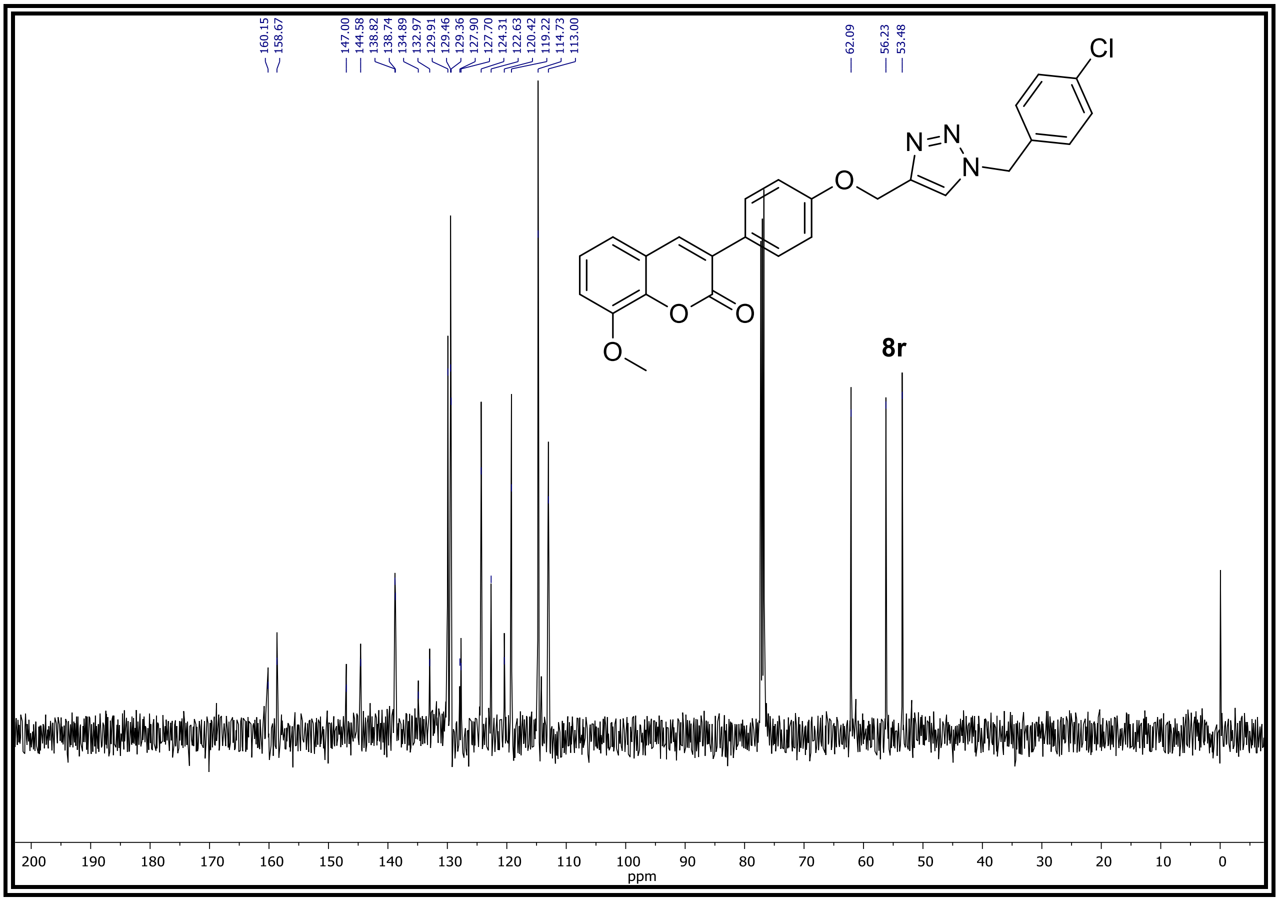

^{13}C NMR (125 MHz, $CDCl_3$) spectrum of 3-(4-((1-(4-Chlorobenzyl)-1*H*-1,2,3-triazol-4-yl)methoxy)phenyl)-8-methoxy-2*H*-chromen-2-one

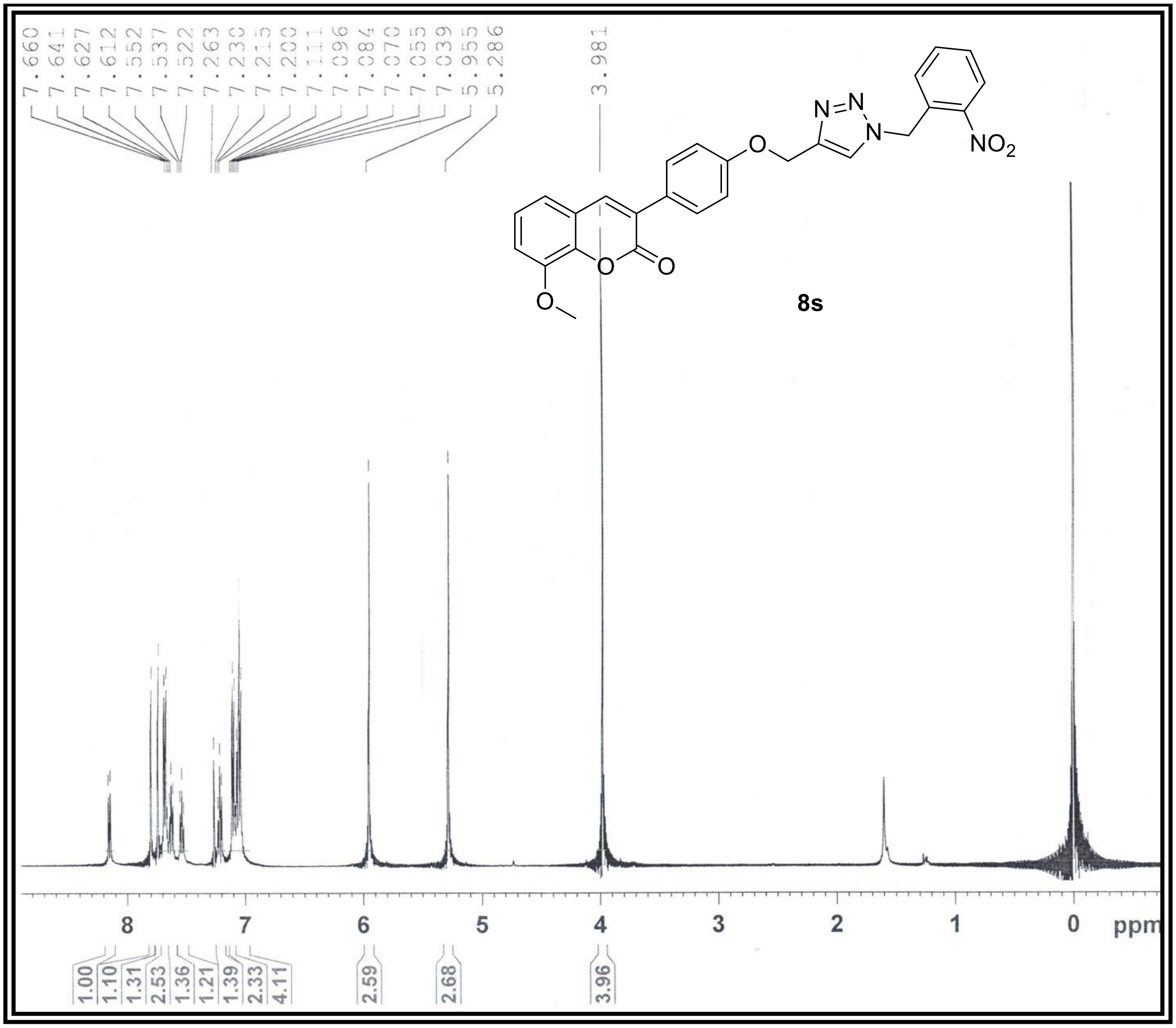

^{1}H NMR (500 MHz, $CDCl_3$) spectrum of 8-Methoxy-3-(4-((1-(2-nitrobenzyl)-1*H*-1,2,3-triazol-4-yl)methoxy)phenyl)-2*H*-chromen-2-one

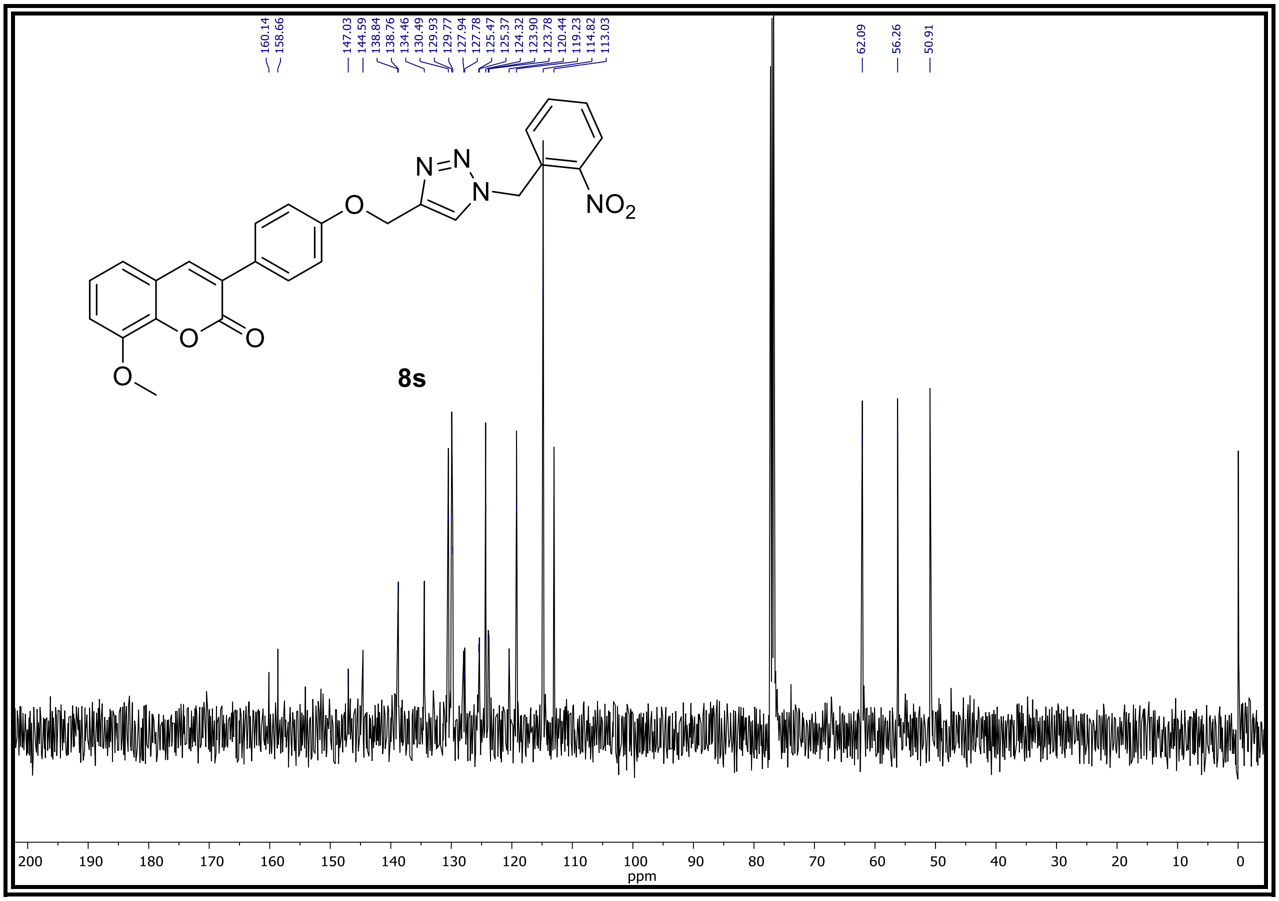

^{13}C NMR (125 MHz, $CDCl_3$) spectrum of 8-Methoxy-3-(4-((1-(2-nitrobenzyl)-1*H*-1,2,3-triazol-4-yl)methoxy)phenyl)-2*H*-chromen-2-one

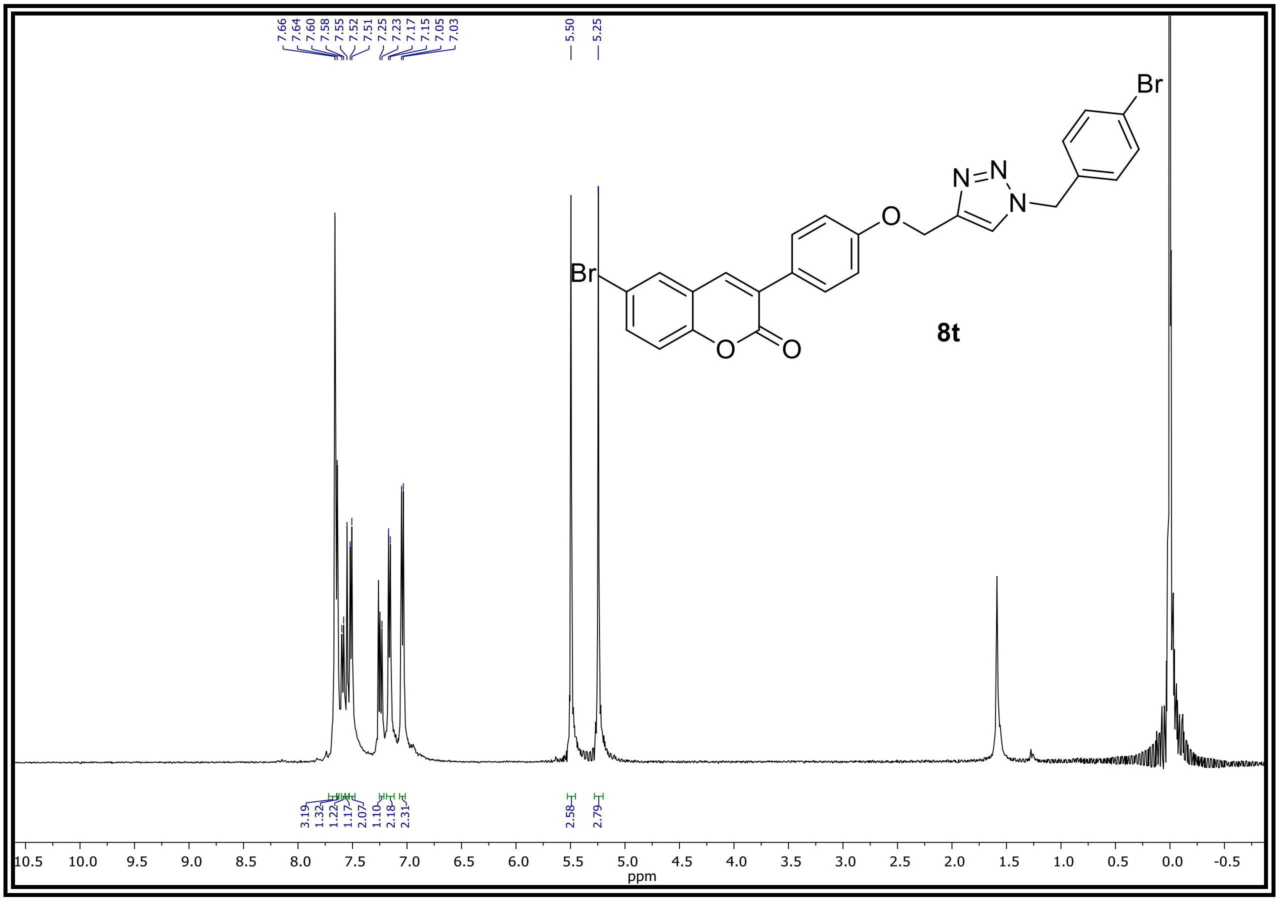

^{1}H NMR (500 MHz, $CDCl_3$) spectrum of 6-Bromo-3-(4-((1-(4-bromobenzyl)-1*H*-1,2,3-triazol-4-yl)methoxy)phenyl)-2*H*-chromen-2-one

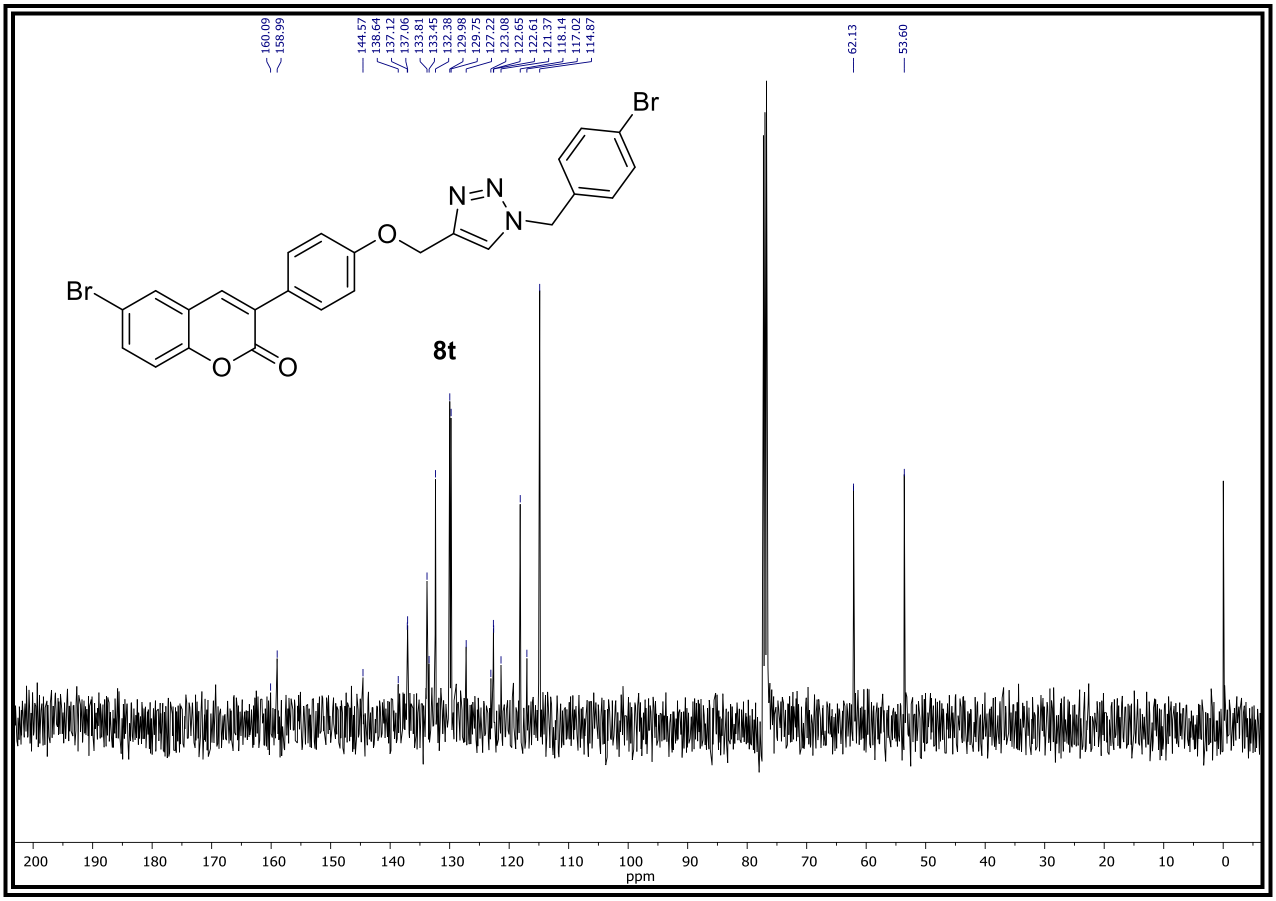

^{13}C NMR (125 MHz, $CDCl_3$) spectrum of 6-Bromo-3-(4-((1-(4-bromobenzyl)-1*H*-1,2,3-triazol-4-yl)methoxy)phenyl)-2*H*-chromen-2-one

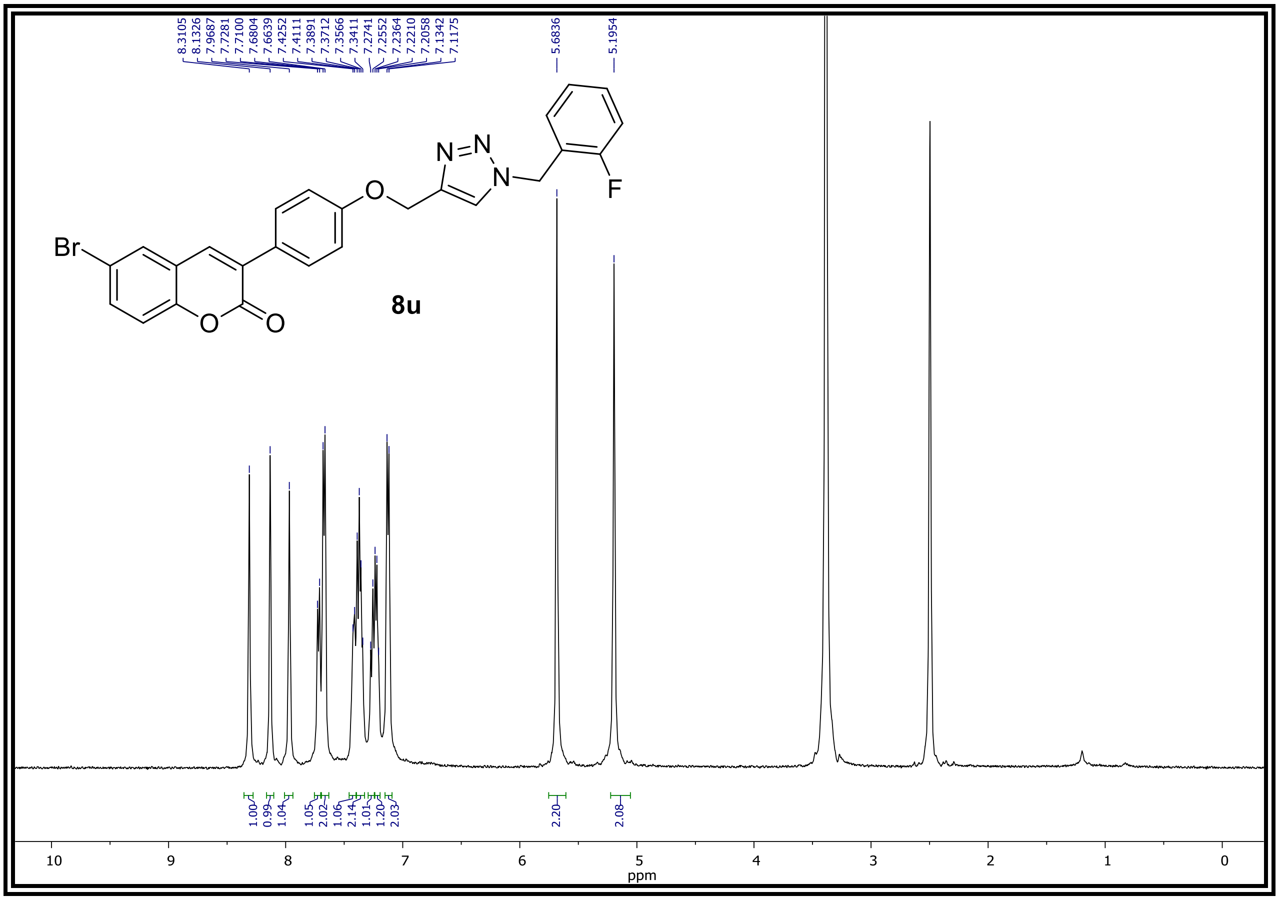

^{1}H NMR (500 MHz, DMSO-d_6) spectrum of 6-Bromo-3-(4-((1-(2-fluorobenzyl)-1*H*-1,2,3-triazol-4-yl)methoxy)phenyl)-2*H*-chromen-2-one

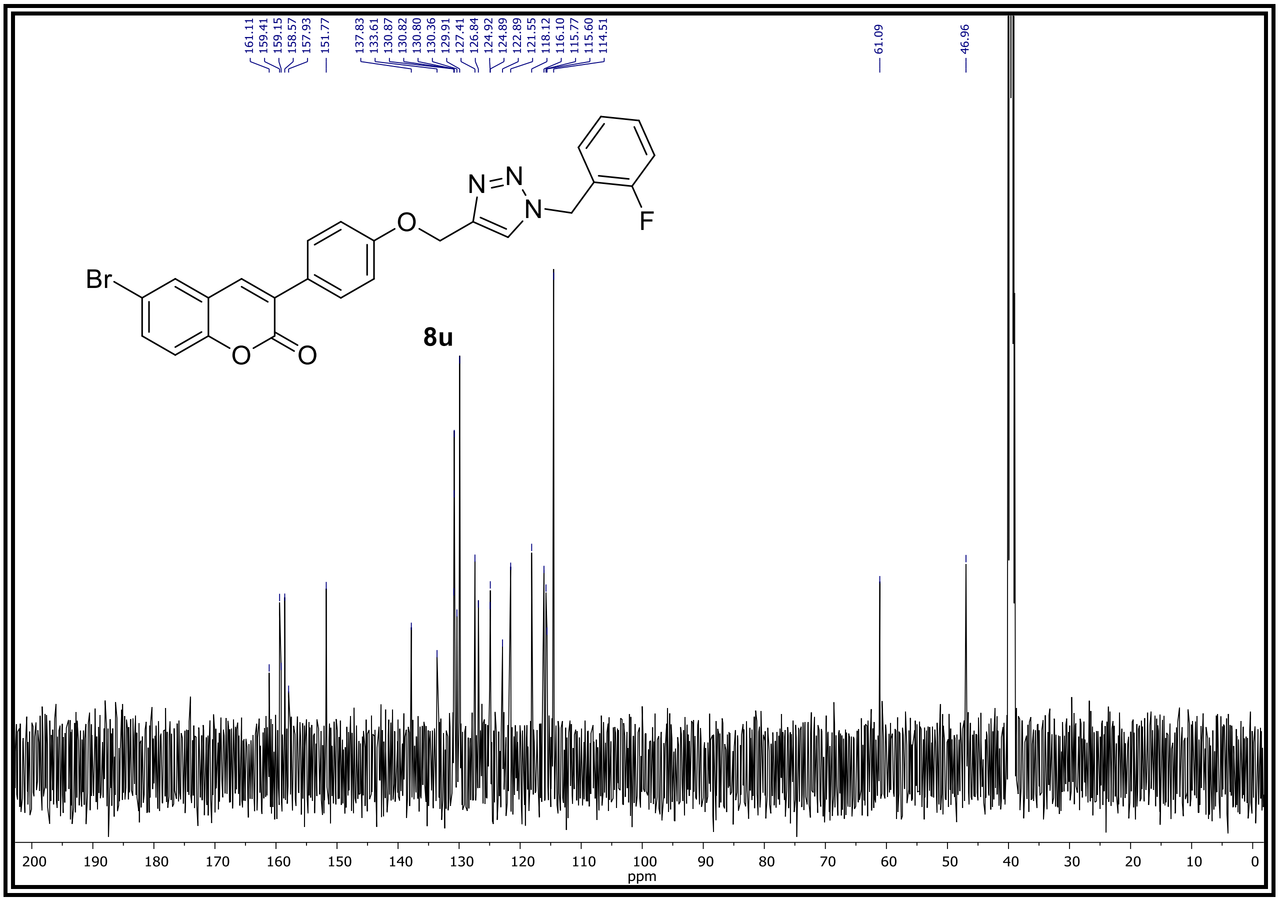

^{13}C NMR (125 MHz, DMSO-d_6) spectrum of 6-Bromo-3-(4-((1-(2-fluorobenzyl)-1*H*-1,2,3-triazol-4-yl)methoxy)phenyl)-2*H*-chromen-2-one

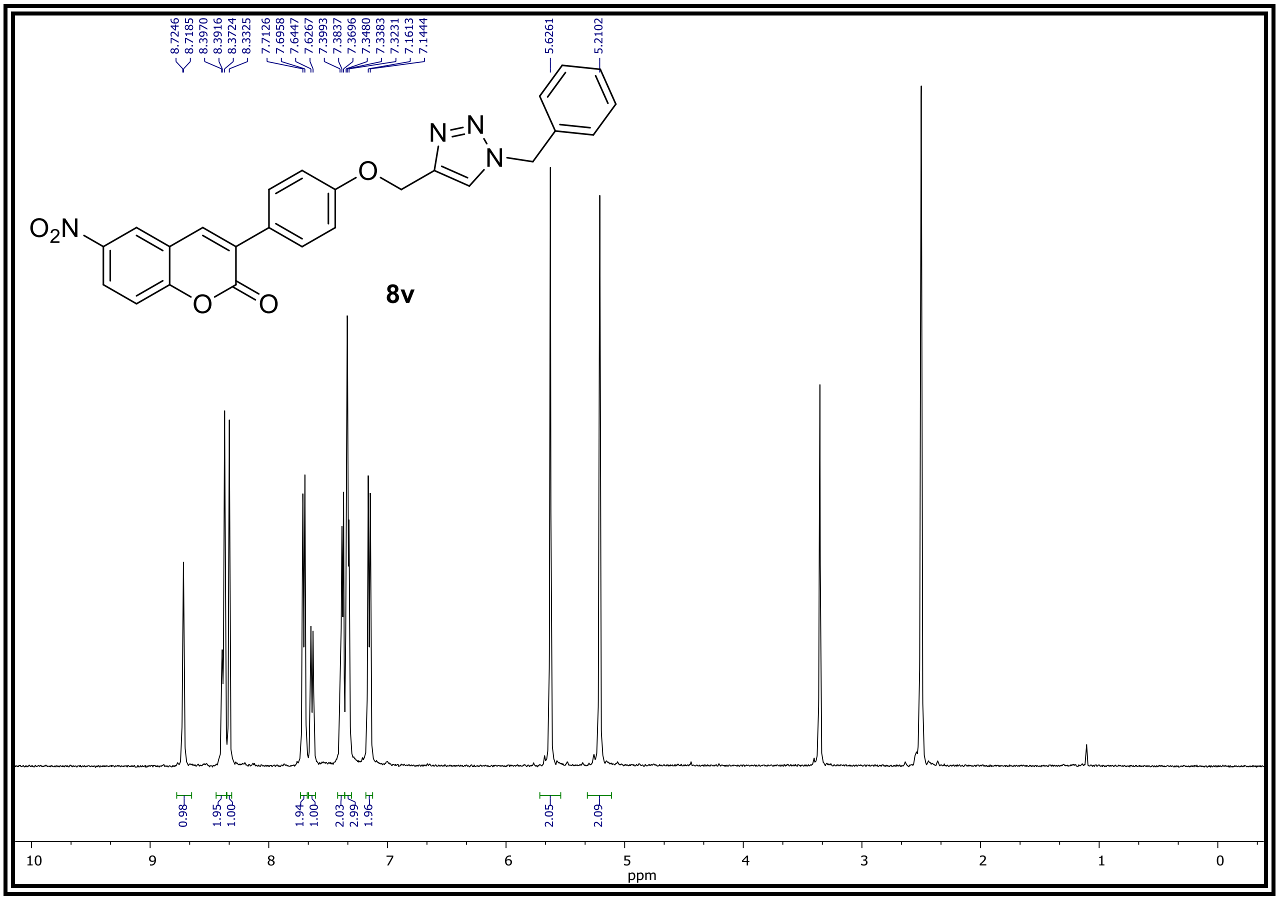

^{1}H NMR (500 MHz, DMSO-d_6) spectrum of 3-(4-((1-Benzyl-1*H*-1,2,3-triazol-4-yl)methoxy)phenyl)-6-nitro-2*H*-chromen-2-one

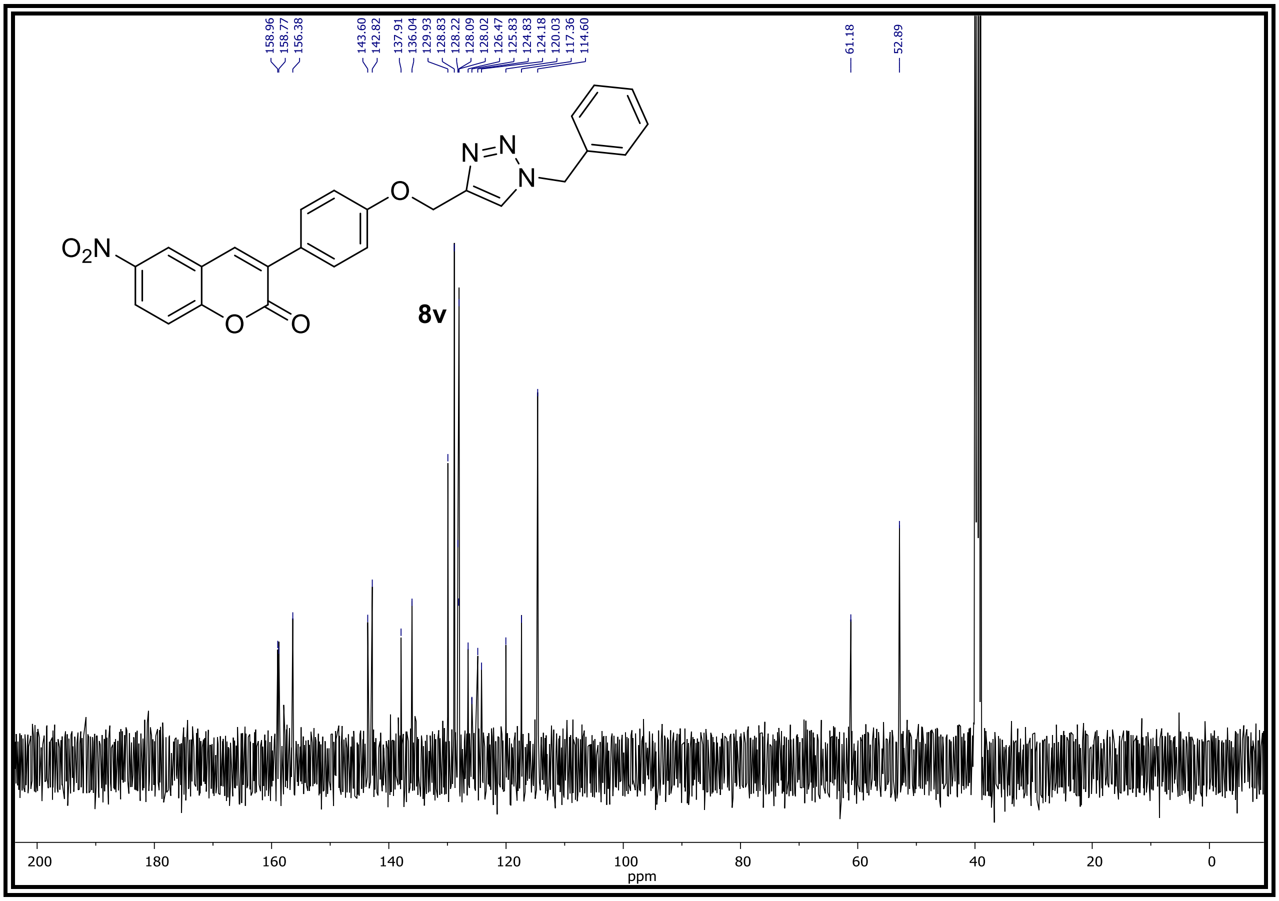

^{13}C NMR (125 MHz, DMSO-d_6) spectrum of 3-(4-((1-Benzyl-1*H*-1,2,3-triazol-4-yl)methoxy)phenyl)-6-nitro-2*H*-chromen-2-one

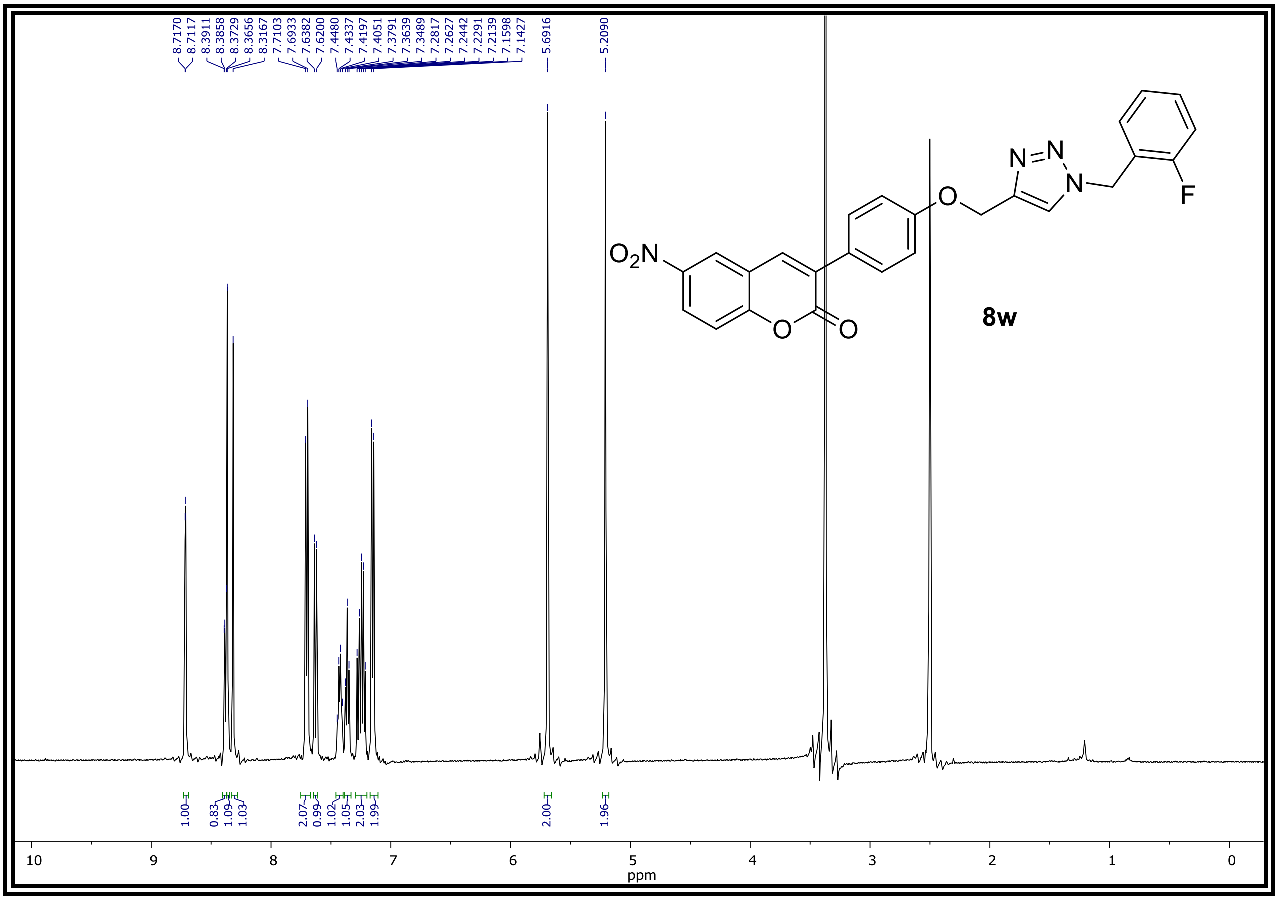

^{1}H NMR (500 MHz, DMSO-d_6) spectrum of 3-(4-((1-(2-Fluorobenzyl)-1*H*-1,2,3-triazol-4-yl)methoxy)phenyl)-6-nitro-2*H*-chromen-2-one

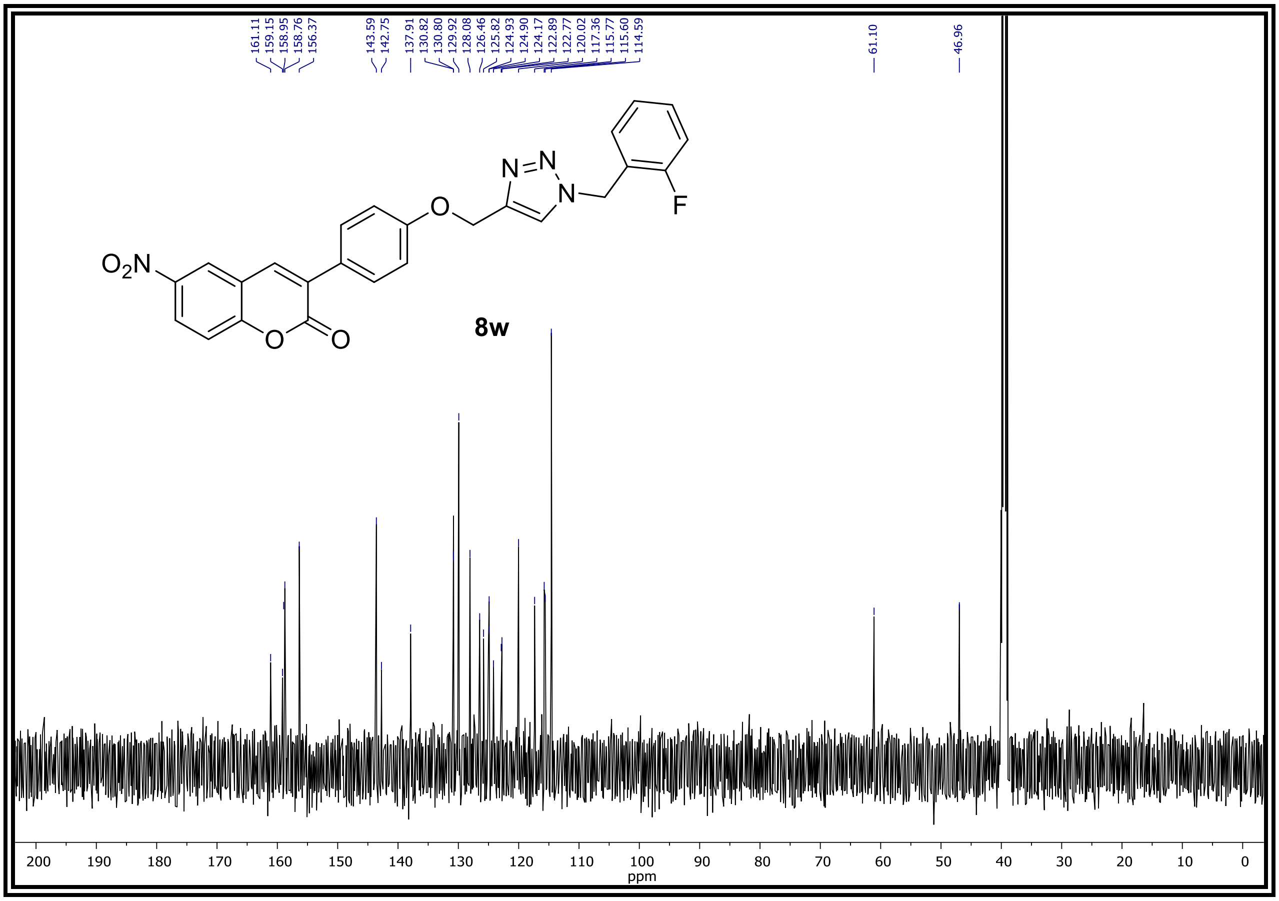

^{13}C NMR (125 MHz, DMSO-d_6) spectrum of 3-(4-((1-(2-Fluorobenzyl)-1*H*-1,2,3-triazol-4-yl)methoxy)phenyl)-6-nitro-2*H*-chromen-2-one
